# Supplementary material for: A gene cluster in Ginkgo biloba encodes unique multifunctional cytochrome P450s that initiate ginkgolide biosynthesis
Source: Nat Commun. 2022 Sep 1;13:5143. doi: 10.1038/s41467-022-32879-9 (PMC9436924; doi:10.1038/s41467-022-32879-9)
Supplement: Supplementary file 1 — Supplementary Information [file 41467_2022_32879_MOESM1_ESM.pdf]

A gene cluster in *Ginkgo biloba* encodes unique multifunctional cytochrome P450s that initiate ginkgolide biosynthesis

Forman *et al.*

Supplementary Method 1. Mining P450s from transcriptomes

Ginkgo cytochrome P450s were found by batch blast search of a ginkgo leaf transcriptome file G*GB*W01.1.fsa_nt.gz obtained from Bioproject PRJNA411881 (https://www.ncbi.nlm.nih.gov/Traces/wgs/?val=G*GB*W01)[1] with a Gymnosperm query set of 62 P450 sequences (Gymnosperm.queryset.txt). This query set has members of all known CYP families from gymnosperms. The search was done using NCBI stand-alone BLAST software. The top 100 hits for each query were retained (6895 hits) and filtered to remove duplicate accessions. 322 hits were extracted from the original G*GB*W01.1.fsa_nt file by the fastacmd command. The DNA sequences were translated to protein sequences using the Virtual Ribosome (https://services.healthtech.dtu.dk/service.php?VirtualRibosome-2.0). The protein sequences were batch BLASTP searched against all named plant P450s to identify CYP family and subfamily.

A second search was carried out against the TSA section at NCBI using a 194 amino acid C-terminal fragment of CYP716B17 (ginkgo) with an expect value of 10 and max 500 hits. This should pull out all ginkgo P450 sequences that include the C-term from the I-helix to the end. This search will not find N-term fragments. 370 different accession IDs were recovered. These sequences were batch BLASTX searched against all named plant P450s to identify CYP family and subfamily. The TSA data included the G*GB*W01 sequences from the earlier search and *GB*YR01 sequences (Bioproject PRJNA270069 from sterile seedlings)[2]. New sequences were found this way and the two sets were combined. A total of 411 sequences were compared against each other to identify duplicates. After excluding Pseudogenes, fragments and duplicates or near duplicates and 11 partial sequences, 161 sequences remained.

Proteins from the annotated Ginkgo genome were downloaded from http://gigadb.org/dataset/view/id/100613/Sample_sort/genbank_name[3]. The file Ginkgo_biloba.HiC.protein.fasta was formatted for Blast searching and searched with the same gymnosperm query set as the leaf transcriptome. 398 unique hits were found and recovered from the fasta file with the fastacmd command. Six sequences contained protein fusions that had to be split, resulting in 404 gene models. These sequences were searched against all named plant P450s which included the previously named ginkgo P450 set. A reduced set of 254 gene models was selected by removing predicted pseudogenes and or fragments. The reduced transcript set was blast searched against the gene model set to find equivalent sequences. 71 transcript sequences did not match the gene models, giving a total of 325 unique sequences. It was possible that the genome contained the missing sequences. The genome Ginkgo_biloba.HiC.genome.fasta was downloaded and split into 12 files equivalent to the 12 chromosomes using the csplit command csplit -k Ginkgo_biloba.HiC.genome.fasta.txt '/>Chr/' . The individual chromosomes were formatted for blast searching

and searched with the 71 transcripts that did not have a matching gene model. 28 transcripts did have a match to the genome leaving 43 without an exact match. Some of these were apparent chimeric sequences that partially matched two gene models. 24 unmatched transcripts were from large ginkgo gene sets CYP76AA, CYP720B, CYP736. If these 43 transcripts are excluded 282 unique P450 sequences remain in the ginkgo genome.

After further refinement of the protein set to remove probable duplicates and short sequences, 318 P450 sequences were used to make two maximum likelihood trees, one for the large CYP71 clan with 145 sequences (Supplementary Figure 1) and one for the nine other CYP clans with 173 sequences (Supplementary Figure 2). The amino acid sequences used for the trees were deduced from genes and transcript sequences identified in genome and transcriptomes of *Ginkgo biloba* respectively. Sequences have been analyzed phylogenetically using Maximum Likelihood method. The evolutionary history was inferred by using the Maximum Likelihood method and JTT matrix-based model[4] . The trees with the highest log likelihood (-81156.42) are shown. The percentage of trees in which the associated taxa clustered together is shown next to the branches. Initial tree(s) for the heuristic search were obtained automatically by applying Neighbor-Join and BioNJ algorithms to a matrix of pairwise distances estimated using a JTT model, and then selecting the topology with superior log likelihood value. The tree is drawn to scale, with branch lengths measured in the number of substitutions per site. Evolutionary analyses for both trees were conducted in MEGA X [5]

Supplementary Method 2. Transient expression in *Nicotiana benthamiana* using pLIFE33

Genes to be transiently expressed in *N. benthamiana* were cloned into the pLIFE33 vector[6] using primers presented in Supplementary Table 16 while cloned pLIFE33 constructs for transient expression in *Nicotiana benthamiana* are presented in Supplementary Table 15.

Supplementary Method 3. Construction of yeast Assembler plasmids for *Saccharomyces cerevisiae*

In this work, we reconstructed plasmids for yeast genomic integration based on previously reported plasmids[7,8]. Generally, each site for genomic integration (such as X-3) can be targeted for genomic integration by combining an assembler 1 and assembler 3 plasmid, each with flanking homologous regions (DW and UP) to the site of choice, and the universal connecting plasmids Assembler 2[8] or the expanded Assembler 2A-2C[9].

Each plasmid contains an uracil-specific-excision-reagent (USER) cassette allowing 1 or 2 genes to be inserted with separate promoters based on the USER overhangs of the amplification primers[10].The single integration pX plasmids[7] (Supplementary Table 17) – allowing up to two genes to be integrated in predefined loci - were used as templates to generate the Assembler 1-3 plasmids by USER fusion cloning[11]. Primers (Supplementary Table 19) were designed using the online AMUSER tool[12]. All generated plasmids were cloned by fusing PCR fragments by USER fusion cloning and plasmids were recovered after transformation into *E. coli* and verified by Sanger sequencing (Macrogen, The Netherlands).

Assembler 1 vectors were generated by amplifying the pX plasmids with primers VEC9+VEC22 and VEC21+VEC10 while amplifying terminator parts tPGI1/tFBA1 from genomic DNA (gDNA) isolated from S288C with primer pairs VEC11+VEC12 and VEC13+VEC14. Assembler 3 vectors were generated by amplifying the pX plasmids with primers VEC15+VEC22 and VEC16+VEC21 while amplifying terminator parts tENO2/tTDH2 from gDNA with primer pairs VEC19+VEC20 and VEC17+VEC18. The Assembler 2 vector was generated by amplifying the pX-2 plasmid with primers VEC1+VEC2 while amplifying terminator parts tFBA1/tTDH2 from gDNA with primer pairs VEC3+VEC4 and VEC7+VEC8 and terminators tPGI1/tENO2 and USER cassette from a *Gb*lock synthetic DNA (Integrated DNA Technologies, USA) with primer pairs VEC5+VEC6. The Assembler 2A was generated by amplifying the pX-2 plasmid with primers VEC23+VEC24 while amplifying terminator parts tSPG5/tPRM9 from gDNA with primer pairs VEC25+VEC26 and VEC27+VEC28 while the USER cassette and terminator pairs tFBA1/tPGI1 were amplified from Assembler 2 using pair VEC29+VEC30. Assembler 2C was generated by amplifying the pX-2 plasmid with primers VEC31+VEC32 while amplifying terminator parts tPRM5/tCPS1 from gDNA with primer pairs VEC35+VEC36 and VEC37+VEC38 while the USER cassette and terminator pairs tENO2/tTDH2 were amplified from Assembler 2 using pair VEC33+VEC34. Assembler 2B was generated by amplifying the pX-2 plasmid with primers VEC39+VEC40 while amplifying terminator parts tPRM5/tCPS1 from Assembler 2C with primer pairs VEC41+VEC42 and USER cassette and terminator pairs tSPG5/tPRM5 were amplified from Assembler 2A using pair VEC43+VEC44.

Supplementary Method 4. *Saccharomyces cerevisiae* related information: primers, codon optimized sequences, constructs, yeast strains

Primers used to clone cDNAs in Assembler plasmids are presented in Supplementary Table 20, primers for promoters, sequencing and genotyping in Supplementary Table 21 while cloned constructs are presented in Supplementary Table 18. Synthetic gene sequences ordered (Integrated DNA Technologies, USA) are

presented in Supplementary Table 22. The genotype of yeast strains generated in this study are presented in Supplementary Table 3.

Promoter sequences for *Saccharomyces cerevisiae* were amplified from S288C gDNA. Generally, the orientation of genes and promoters were termed either PGK or TEF depending on what USER overhangs were used: genes in the PGK direction contained the forward overhang 5´-ATCAACGGGU-3´ and reverse 5´ CGTGCGAU-3´ matching a promoter in the PGK direction with reverse overhangs 5´-ACCCGTTGAU- 3´. Genes in the TEF direction had a forward 5´-AGCGATACGU-3´ and reverse 5´- CACGCGAU-3´ matching a TEF promoter with reverse 5´- ACGTATCGCU-3´. Dual promoters were assembled replacing the forward primer in the PGK direction with overhang 5´-ACCTAAGTCU-3´ and TEF direction forward 5´-AGACTTAGGU- 3´.

*Gb*LPS was fused to a maltose-binding protein (MBP) in the C-terminal end by USER cloning using specific USER primers (Supplementary Table 20) designed using the online AMUSER tool[13]. The stop codon of *Gb*LPS was replaced by a six-glycine linker fusing the *Gb*LPS peptide to the MBP at the C-terminal end.

Supplementary Method 5. Isolation of compounds produced by *Saccharomyces cerevisiae* for NMR analysis

Up to 500 mL of Saccharomyces cerevisiae strain sGIN7 (expressing GbCYP7005C1) and sGIN11 (Expressing GbCYP7005C1, GbCYP7005C3 and GbCYP867E38) were grown in the fed-batch like medium. The cultures were grown in baffled Erlenmeyer shake flasks at 30°C, 180 RPM. After 72 h of growth, yeast pellets and clear medium were separated by centrifugation for 15 min at 3,000 g. Metabolites were extracted from the pellets by adding 50 mL of 100% methanol followed by cold extraction for 3 h at 4 °C. Samples were cleared by centrifugation for 15 min at 3,000 g. After removal of the solvent by rotary evaporation (Buchi, Switzerland), the residue was subjected to flash chromatography using the Buchi Pure Chromatography Systems (PuriFlash® Preparative Purification Systems model: PF-5.250) equipped with a silica gel 60 column (puriFlash®, PF-25SIHC-F0025) at a flow rate of 15 mL min–1. The mobile phases were *n*-hexane (A) and ethyl acetate (EtOAc) (B). Separations were performed using the following gradient profile in column volumes (CV): 2 CV, 0% B; 14 CV, 70% B; 16 CV min, 100% B).

Samples were further separated using preparative HPLC separation. This was performed using an Agilent 1200 series instrument (Santa Clara, CA) consisting of a G1311A quaternary pump, a G1322A degasser, a G1316A thermostatated column compartment, a G1315C photodiode-array detector, a G1367C high-performance auto sampler, and a G1364C fraction collector, all controlled by Agilent ChemStation version B.03.02 software.

The fraction containing compounds was separated on a Phenomenex Kinetex XB-C18 column (150 mm × 4.6 mm i.d., 3 µm particle size, 100 Å pore size) (Phenomenex, Inc., Torrance, CA, USA) at a flow rate of 0.5 mL min–1 with column temperature held at 40 °C. The mobile phases were 5% acetonitrile (A) and 95% acetonitrile (B), both acidified with 0.5% formic acid. Separations were performed using the following gradient profile: 0 min, 50% B; 18 min, 95% B; 19min, 100% B; 29 min, 100% B; 30 min, 50% B; 38 min, 50% B. A total of 20 cumulative injections were performed using 10 µL injection volumes.

Supplementary Method 6. Isolation of compounds produced by *Nicotiana benthamiana* for NMR analysis

Up to 40 individual *N. benthamiana* plants (4-6 weeks old) were infiltrated with agrobacteria culture containing cDNA encoding SpGGPPS7, SctHMGR, tGbLPS, GbCYP7005C1, GbCYP867E38, GbCYP867K1 and GbCYP720B31. After 7 d of growth, the filtrated plants were extracted with 500 mL methanol. The methanol extract was washed with *n*-hexane (3 × 500 mL) and dried over anhydrous magnesium sulphate (MgSO4). After removal of the solvent by rotor evaporation, the residue (5.0 g) was partitioned on Supelclean™ LC-18 SPE Cartridges (Sigma-Aldrich St. Louis, MO, USA) eluting sequentially with water-acetonitrile (v/v) 9:1, 5:1, 3:1, 2:1 and 1:1, yielding five subfractions (Fractions 1–5). Fractions 3 and 4 were combined (33.1 mg) and further purified by the preparative HPLC using the same equipment and setup as described above. Separations were performed using the following gradient profile: 0 min, 25% B; 18 min, 60% B; 19 min, 100% B; 34 min, 100% B; 35 min, 25% B; 46 min, 25% B. A total of 40 cumulative injections were performed using 5 µL injection volumes.

Supplementary Method 7. NMR analysis

NMR experiments were performed with a Bruker Avance III NMR system (operating at ^{1}H frequency of 600.13 MHz, ^{13}C 150.90 MHz) equipped with a Bruker SampleJet sample changer and a cryogenically cooled gradient inverse triple-resonance 1.7-mm TCI probe-head (Bruker Biospin, Karlsruhe, Germany) optimized for ^{1}H and ^{13}C observation. Spectra of ginkgosinoic acid A (**7**), ginkgosinoic acid B (**11**), 2-

hydroxylevopimaradiene (**9**), and 2-hydroxydehydroabietadiene (**8**) were acquired in chloroform-*d* whereas spectra of ginkgosinoic acid C glucoside (**13d**), ginkgolactone C glucoside (**18c**), ginkgolactone D glucoside (**19**), ginkgosinoic acid C 1,4 benzoquinone (**17**), and ginkgolactone C 1,4 benzoquinone (**23**) were acquired in methanol-d_4. IconNMR ver.4.2 (Bruker Biospin, Karlsruhe, Germany) was used for controlling automated sample change and acquisition whereas Topspin ver. 3.5 (Bruker Biospin, Karlsruhe, Germany) was used for acquisition and processing of NMR data.

One-dimensional ^{1}H spectra were acquired in automation (temperature equilibration to 300K, optimization of lock parameters, gradient shimming, and setting of receiver gain) using 30° pulses, a spectral width of 20 ppm, acquisition time of 2.72 s, relaxation delay of 1.0 s, and 64 k data points; whereas ^{13}C spectra were acquired with 30° pulses with spectral width 240 ppm, acquisition time 0.90 s and relaxation delay 2.0 s and 64 k data points. DQF-COSY and ROESY spectra were acquired using a gradient-based pulse sequence with spectral width 20 ppm and 2 k×512 data points (processed with forward linear prediction to 1 k data points). HSQC spectra were acquired with spectral width 12 ppm for ^{1}H and 170 ppm for ^{13}C, 2 k×256 data points (processed with forward linear prediction to 1 k data points), and relaxation delay 1.0 s. HMBC spectra were acquired with spectral width 12 ppm for ^{1}H and 240 ppm for ^{13}C, 2 k×128 data points (processed with forward linear prediction to 1 k data points), and relaxation delay 1.0 s.

Supplementary Method 8. Viscozyme treatment of ginkgosinoic acid C glucoside and ginkgolactone C glucoside

Viscozyme treatment was conducted with 10 mL Viscozyme® L cellulolytic enzyme mixture (Novozyme Corp.) and 100 µL of ginkosic acid C glucoside and ginkgolactone C glucoside in a methanol solution (1 mg/mL) in a total volume of 12 mL of 100 mM citrate phosphate buffer (pH 6.0). The reactions mixtures were incubated at 37°C over-night products formed extracted into ethyl acetate. After removal of the solvent, the residues were resuspended in methanol and further purified by preparative HPLC using the same equipment and setup as described above. Separations were performed using the following gradient profile: 0 min, 50% B; 18 min, 95% B; 19min, 100% B; 29 min, 100% B; 30 min, 50% B; 38 min, 50% B.

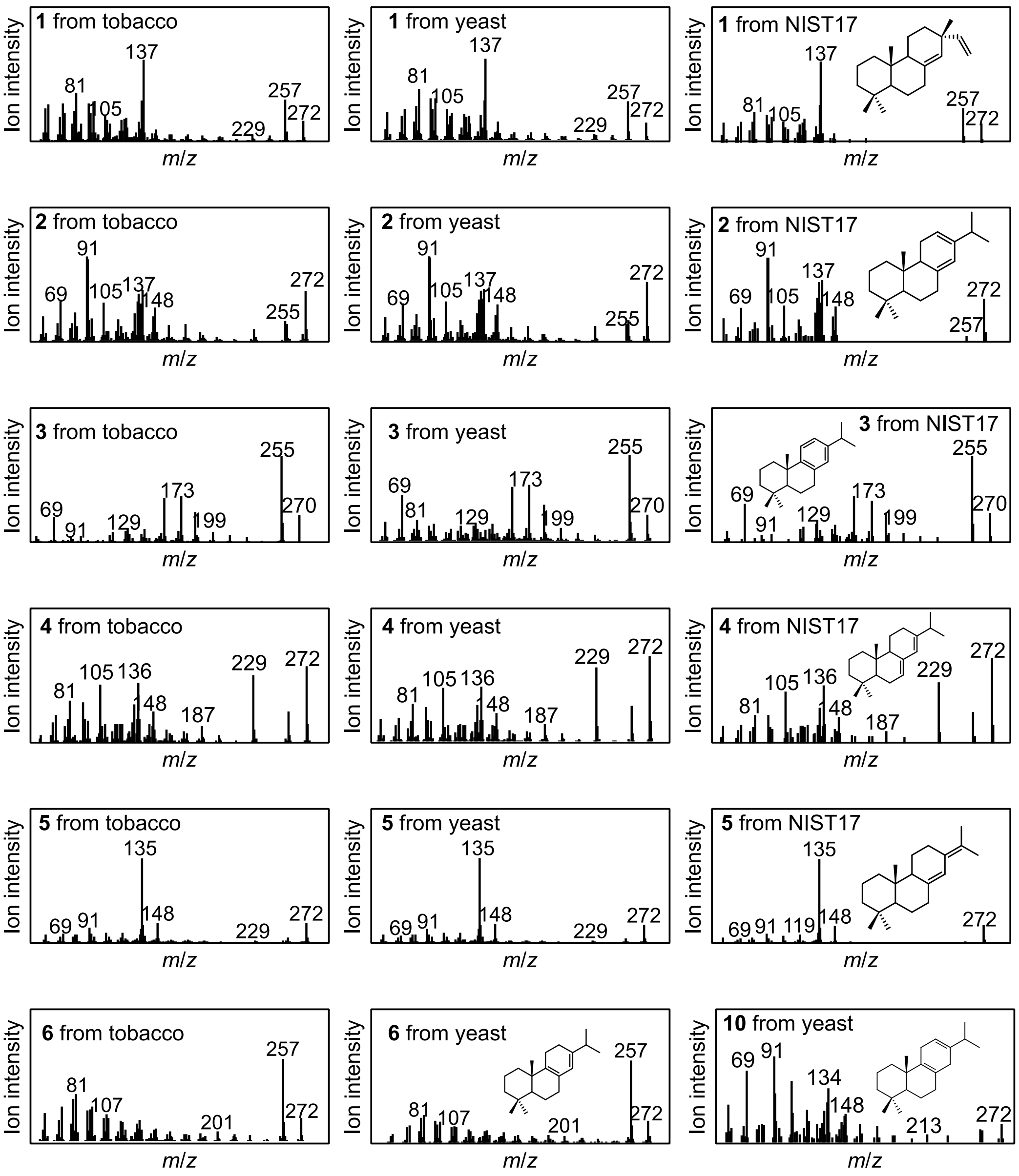

Supplementary Figure 1. GC-MS fragmentation of reference spectra from the National Institute of Standards and Technology 2017 library (NIST17) for compounds produced by *Gb*LPS and other diterpene synthases, in tobacco and yeast. Compound **6** was identified by comparing with the spectra in Liu *et al*.[13] while **10** was indentified by comparing with reference from Andersen-Ranberg *et al.*[14].

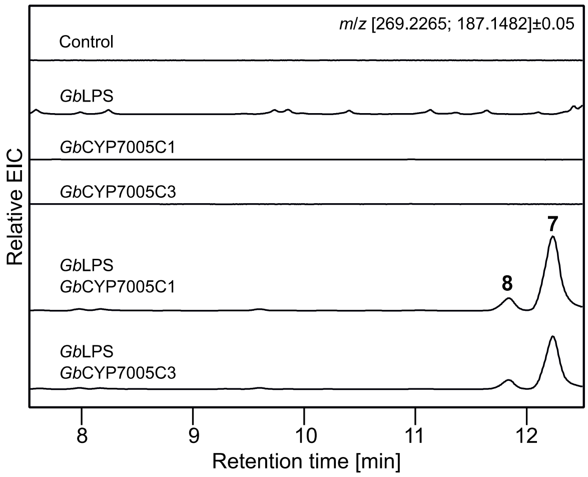

Supplementary Figure 2. Transient expression of *Gb*CYP7005C1 and *Gb*CYP7005C3 alone and with *Gb*LPS in *Nicotiana benthamiana*. Liquid-chromatography-high-resolution-mass-spectrometry (LC-HRMS) analysis of selected *m*/*z* signals in positive mode of methanol extracts of tobacco leaves transiently expressing individual candidate *Gb*CYPs alone and in combination with *Gb*LPS. EIC: Extracted-ion-chromatogram.

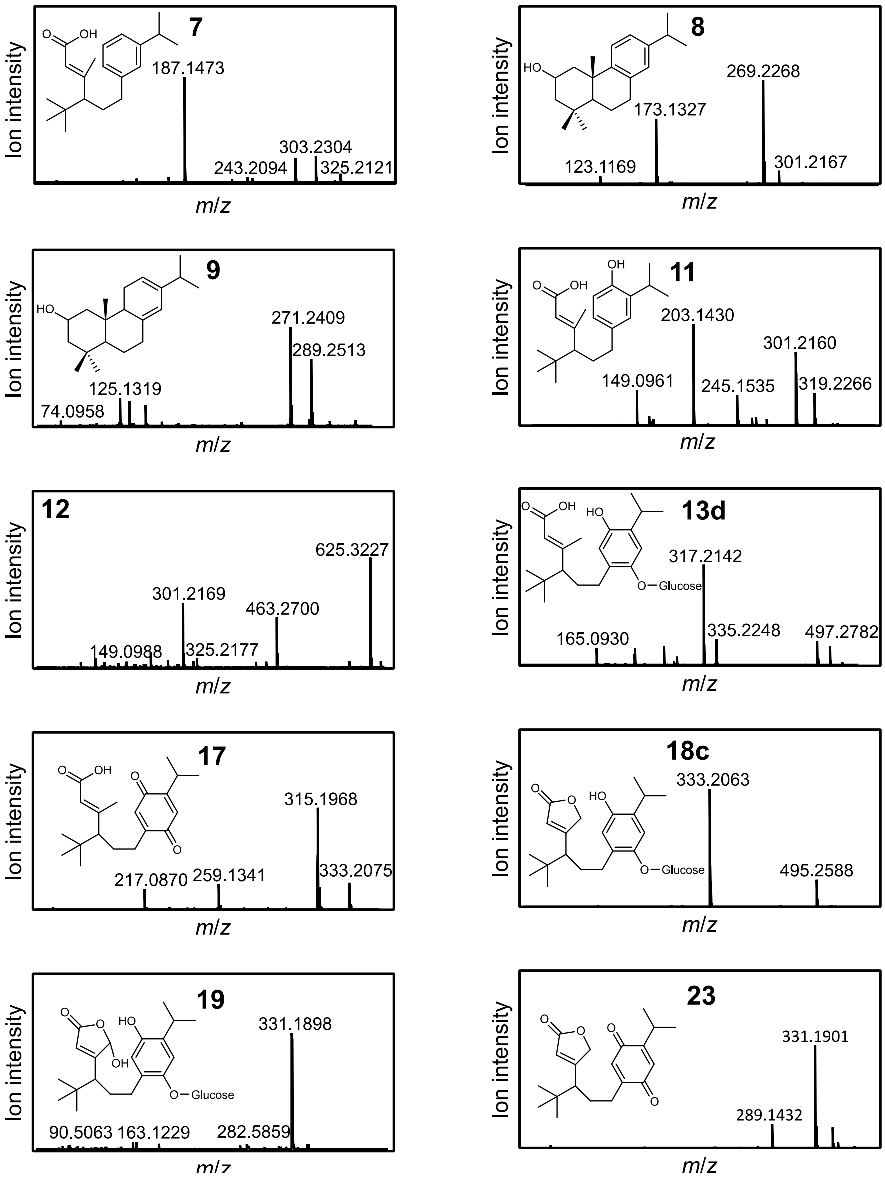

Supplementary Figure 3. **MS spectra of the main molecules described in this work and corresponding elucidated structures.** Recorded MS spectra obtained from the liquid-chromatography-high-resolution-mass-spectrometry (LC-HRMS) analysis of the relevant molecules produced in tobacco and yeast.

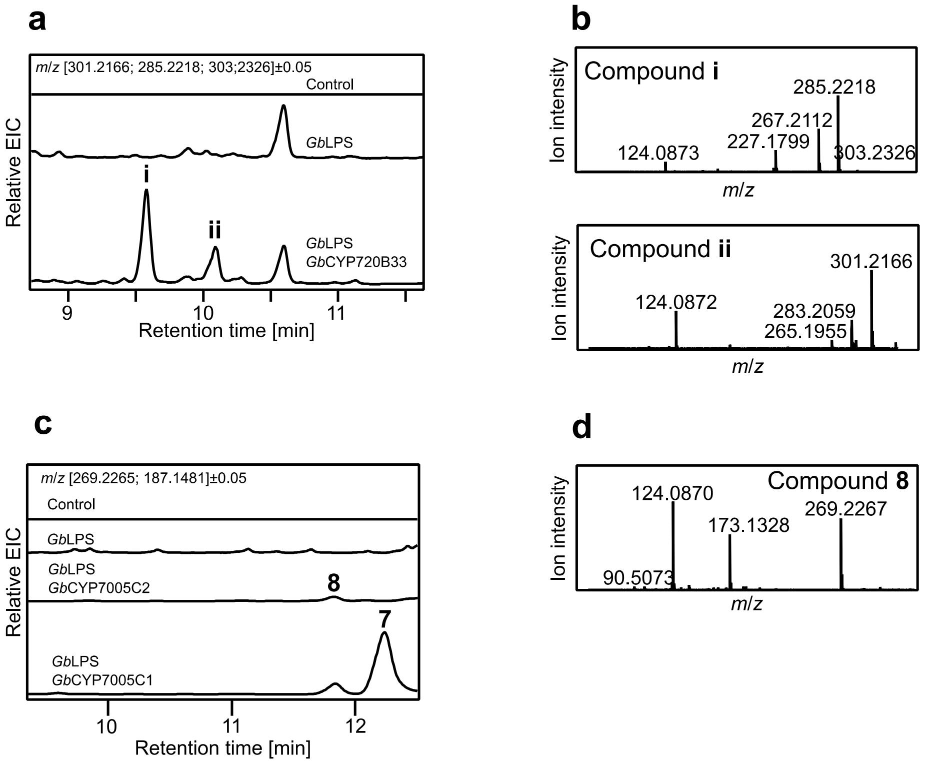

Supplementary Figure 4. Transient expression of *Gb*LPS, *Gb*CYP720B33 and *Gb*CYP7005C2 in *Nicotiana benthamiana*. *Gb*CYP7005C2 is an enzyme encoded in the identified BGC, while *Gb*CYP720B33 was identified from the *Gb*LPS co-expression module. **a,** LC-HRMS analysis of methanol extracts of tobacco leaves transiently expressing *Gb*LPS alone or in combination with *Gb*CYP720B33. As controls, we expressed suppressor of silencing protein P19, *Cf*DXS and *Cf*GGPPS. The chromatograms shown are extracted ion chromatograms (EICs) of the indicated *m/z* values. Two new main peaks were observed (**i** and **ii**). **b**, MS spectra of compounds **i** and **ii**. Compound **i** was predicted to have the formula $C_{20}H_{30}O_2$ while compound **ii** was predicted to have formula $C_{20}H_{28}O_2$. **c,** LC-HRMS analysis of methanol extract of tobacco leaves transiently expressing *Gb*LPS alone or in combination with *Gb*CYP7005C2. As controls, we expressed suppressor of silencing protein P19, *Cf*DXS and *Cf*GGPPS, as well as a combination of *Gb*LPS and *Gb*CYP7005C1. The chromatograms shown are EICs of the indicated *m/z* values. *Gb*CYP7005C2 produced small amounts of compound **8**, which is also produced by *Gb*CYP7005C1/C3. **d,** MS spectum of compound 8.

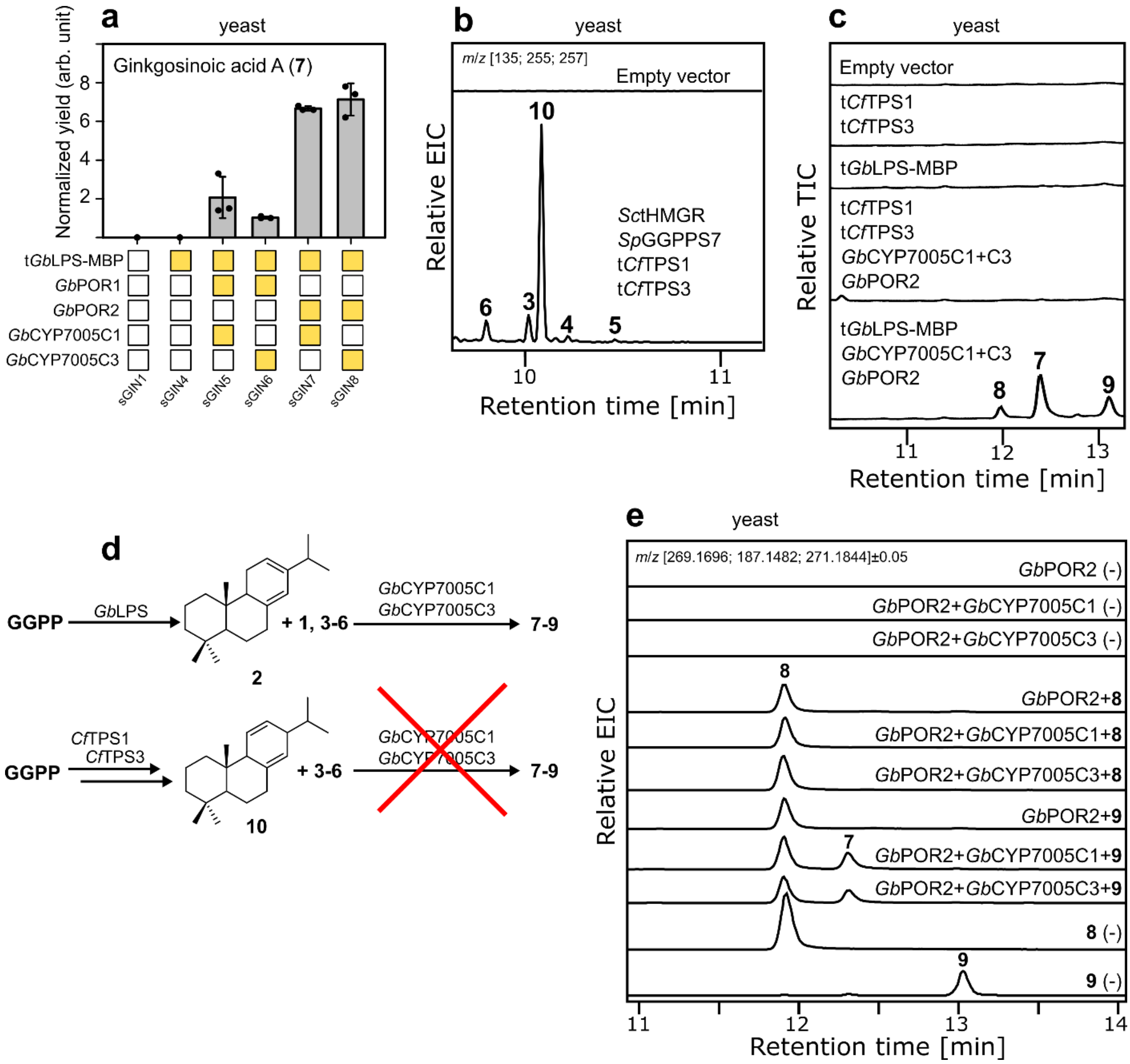

Supplementary Figure 5. Production of ginkgosinoic acid (7) in yeast. a, Optimization of the production of **7** using *Gb*POR1 or *Gb*POR2 in yeast strains, as monitored by LC-HRMS analysis of methanolic extracts. Values represent normalized yield (the peak area of **7** divided by the area of the internal standard) in arbitrary units (AU). Error bars indicate standard deviation of n=3 biological independent replicates. **b**, GC-MS analysis of hexane extracts from yeast strains expressing the truncated diterpene synthase pair *Cf*TPS1 and *Cf*TPS3. The chromatograms show extracted ion chromatograms (EICs) for the *m*/*z* values of 135; 255; and 257. The major peaks were identified as compounds **3-6** and **10**. **c**, production of **7-9** in yeast strains expressing *Gb*CYP7005C1 and *Gb*CYP7005C3, with *Gb*LPS or *Cf*TPS1 and *Cf*TPS3, as identified by LC-HRMS analysis of methanolic yeast extracts. Chromatograms show total ion chromatograms (TIC). **d**, Schematic summary of observations regarding the conversion of GGPP to compounds **7-9** in yeast strains expressing variable diterpene synthases as well as *Gb*CYP7005C1 and *Gb*CYP7005C3. **e**, LC-HRMS analysis of methanolic extracts of yeast strains fed with compounds **8** or **9**. Chromatograms show EICs for the *m*/*z* values of 269.1696; 187.1072; and 271.1844, each with a window of ± 0.05. **7** was only formed when **9** (and not **8**) was fed to yeast strains expressing *Gb*POR2 with either *Gb*CYP7005C1 or *Gb*CYP7005C3. Source data are provided as a Source Data file.

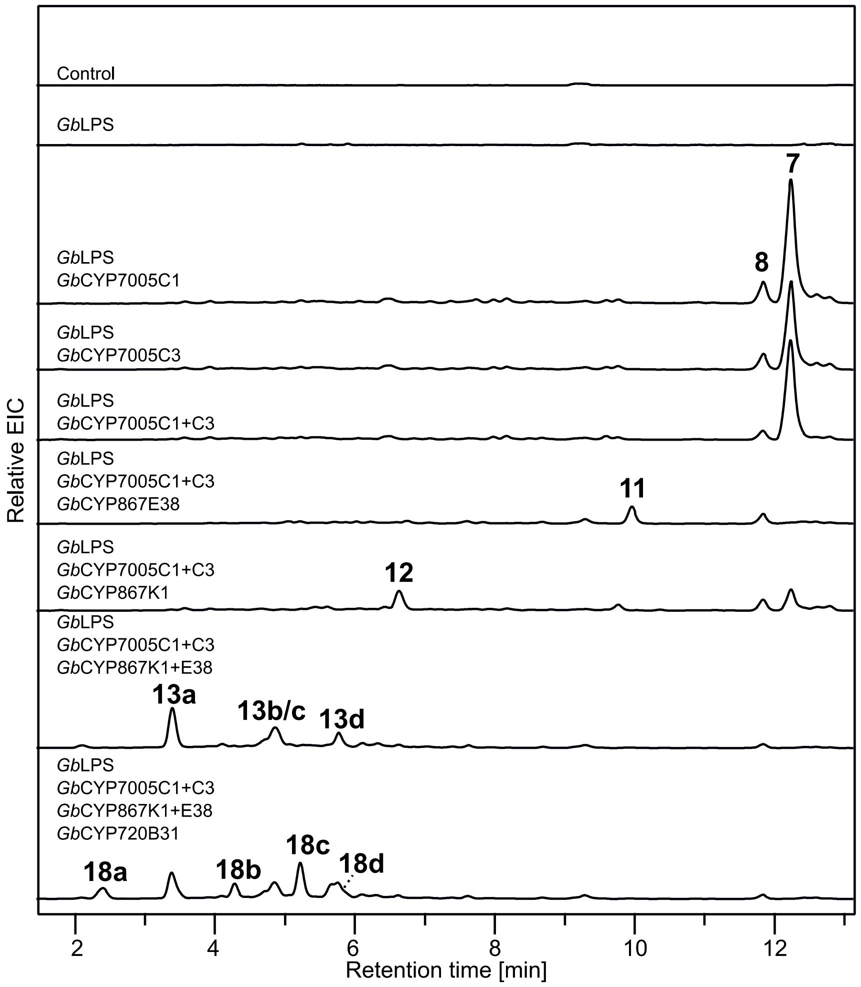

Supplementary Fig 6. Transient expression of *Gb*LPS and candidate *Gb*CYPs in *Nicotiana benthamiana*. Liquid-chromatography high-resolution mass spectrometry (LC-HRMS) analysis (selected *m*/*z* signals in positive mode [187.1482; 203.1425; 269;.2267; 317.2118; 333.2064; 335.2218; 625.3231]±0.05) of methanol extracts of tobacco leaves transiently expressing candidate *Gb*CYPs in combination with *Gb*LPS. EIC: extracted ion chromatograms

a

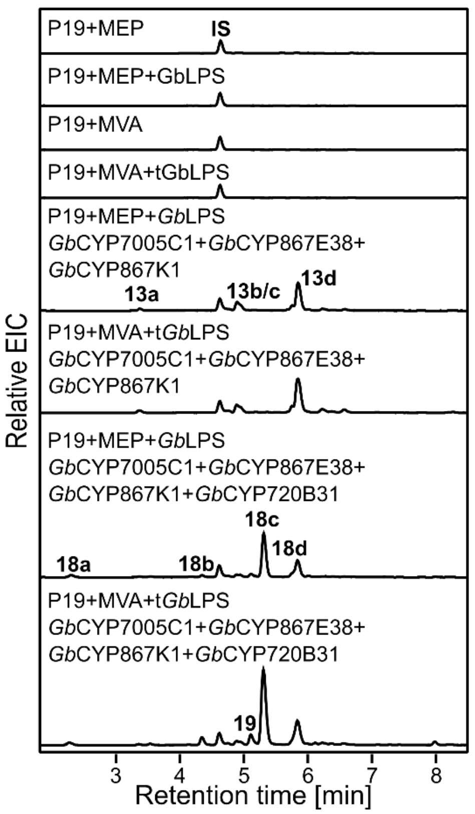

b

Percentage increase using the cytosolic GGPP from the MVA

Combi	13a	13b/c	13d	18a	18b	18c	18d	19
*Gb*CYP7005C1/C3<br>*Gb*CYP867E38<br>*Gb*CYP867K1	127%	75%	82%					
*Gb*CYP7005C1/C3<br>*Gb*CYP867E38<br>*Gb*CYP867K1<br>*Gb*CYP720B31	59%	68%	39%	68%	276%	94%	181%	328%

Supplementary Fig 7. Increased oxygenation of diterpenoid hydrocarbons (*Gb*LPS products) by *Gb*CYP867K1 and *Gb*CYP720B31 when *Gb*LPS in expressed in the cytosol (MVA pathway) of *N. benthamiana* cells in comparison to plastids (MEP pathway). a, LC-HRMS chromatograms of selected ions in positive mode (EIC of *m*/*z* [187.1482; 335.2218; 333.2064; 317.2118; 315.1960; 331.1909] ± 0.05) of methanol-extracted tobacco leaves. **b**, Percentage increase of *Gb*CYP867K1 and *Gb*CYP720B31 products using cytosolic GGPP versus plastidial GGPP. Source data are provided as a Source Data file.

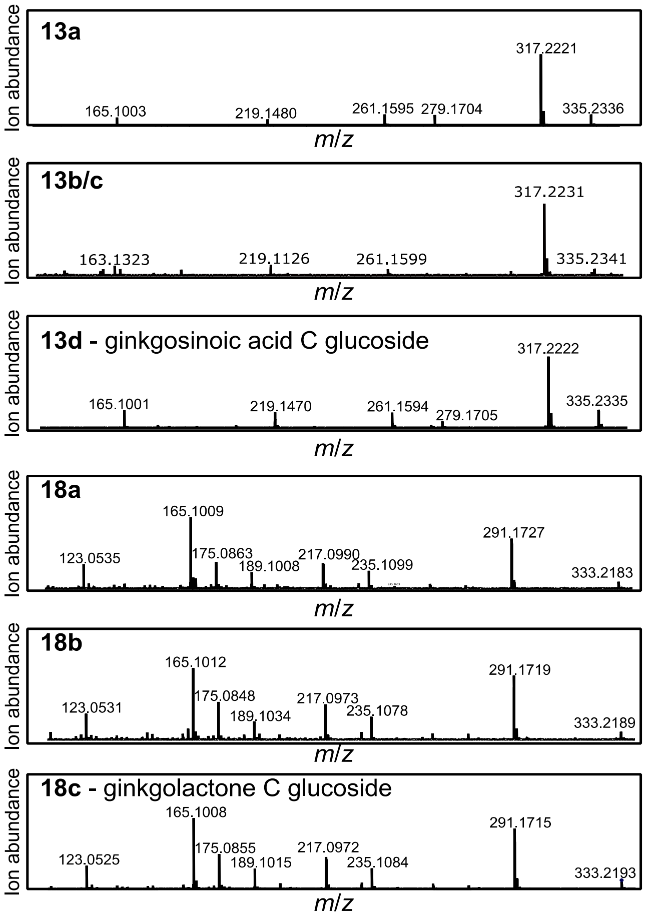

Supplementary Figure 8. MS^2 patterns of diterpenoid glucosides isolated from tobacco expressing *Gb*LPS together with the relevant CYP enzymes. The recorded MS2 spectra obtained from the liquid-chromatography-high-resolution-mass-spectrometry (LC-HRMS) analysis of molecules produced in tobacco. The MS2 patterns showed that the glucosilated **13a-13d** were likely originated from the same aglycone terpenoid, namely ginkgosinoic acid C, and likewise that the glucosilated **18a-18c** were likely originated from the same aglycone terpenoid, namely ginkgolactone C.

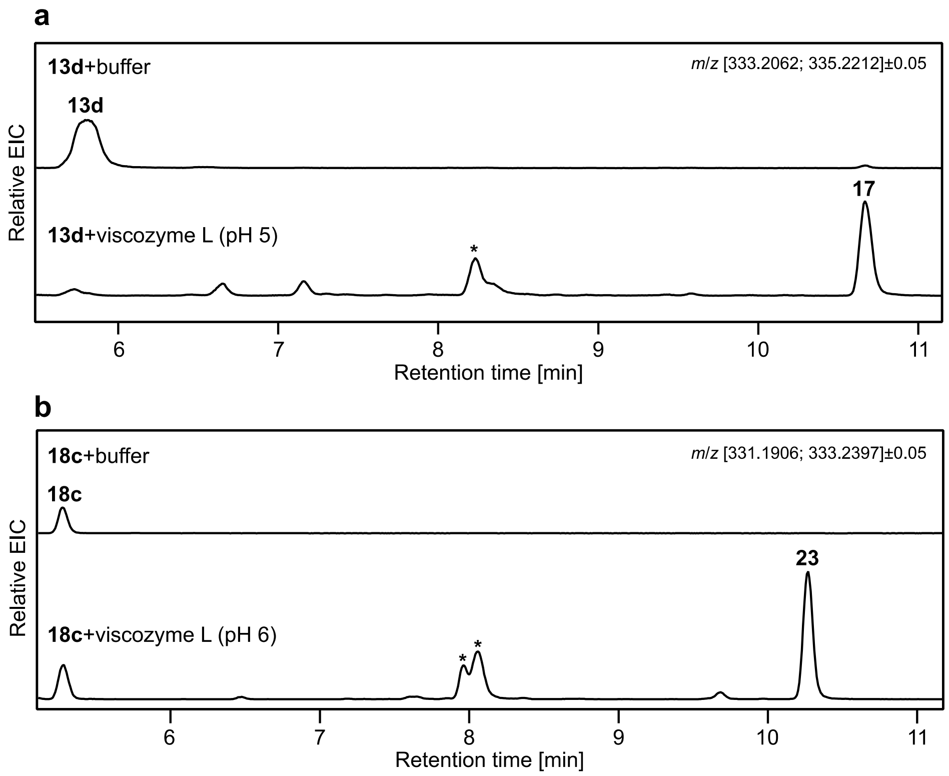

Supplementary Figure 9. Viscozyme L treatment of 13d and 18c. a, LC-HRMS analysis of the treatment of **13d** with Viscozyme L, leading to the formation of **17** along with a molecule with a mass corresponding to free ginkgosinoic acid C (marked with *****, *m/z* 335). The chromatograms shown are EICs of *m/z* values indicated in the top right corner. **b,** LC-HRMS analysis of the treatment of **18c** with Viscozyme L, leading to the formation of **23** along with molecules with masses corresponding to free ginkgolactone C (marked with *****, *m/z* 333). The chromatograms shown are EICs of *m/z* values indicated in the top right corner.

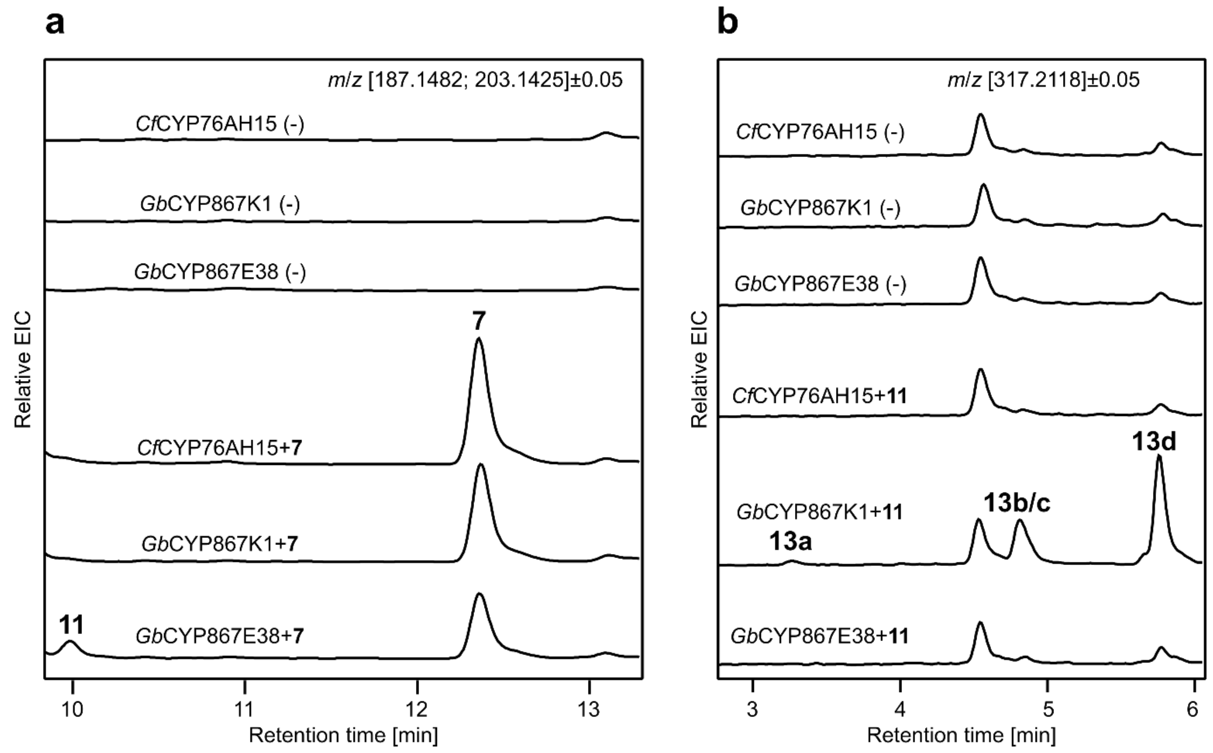

Supplementary Figure 10. Substrate feeding studies in *N. benthamiana* leaves. A, LC-HRMS analysis of methanolic extracts of *N. benthamiana* leaves expressing relevant CYPs and co-infiltrated with ginkgosinoic acid A (compound **7**). *Cf*CYP76AH15 from *Coleus forskohlii* served as negative control. Chromatograms show extracted ion chromatograms (EICs) for the *m*/*z* values of 187.1482; and 203.1425, each with a window of ± 0.05. Only the expression of *Gb*CYP867E38 led to production of ginkgosinoic acid B (compound **11**). **b**, LC-HRMS analysis of methanolic extracts of *N. benthamiana* leaves expressing relevant CYPs and co-infiltrated with ginkgosinoic acid B (compound **11**). *Cf*CYP76AH15 from *Coleus forskohlii* served as negative control. Chromatograms show EIC for the *m*/*z* values of 317.2118 with a window of ± 0.05. Only the expression of *Gb*CYP867K1 led to production of **13a-13d**.

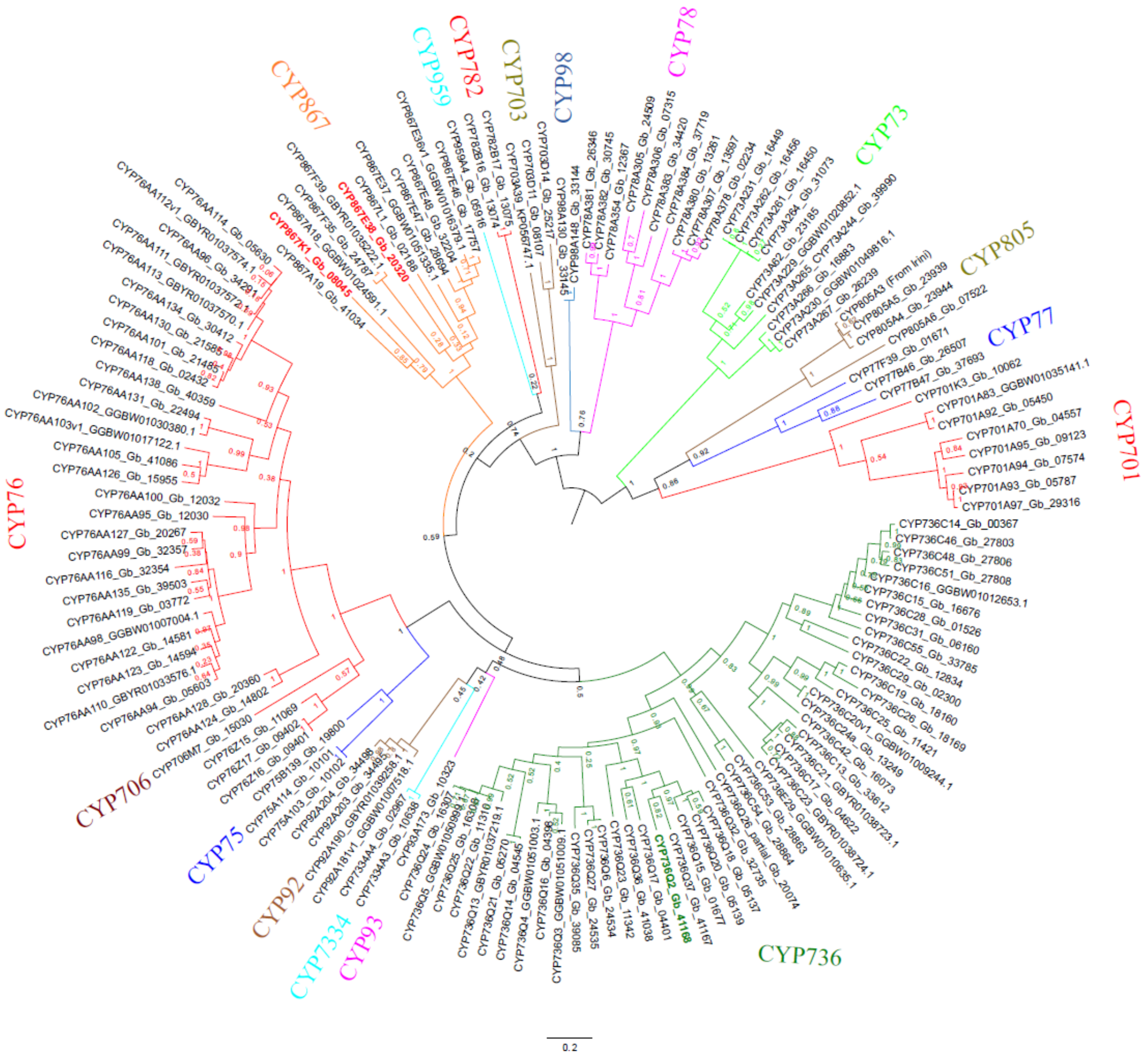

Supplementary Figure 11. Evolutionary relations of the *Ginkgo biloba* CYP enzymes in the CYP71 clan. This analysis involved 145 amino acid sequences. There was a total of 492 positions in the final dataset. The tree is drawn to scale, with branch lengths measured in the number of substitutions per site. In red-bold font are marked the CYPs found in the Biosynthetic Gene Cluster, and in green-bold font are marked the CYPs co-expressed with *Gb*LPS.

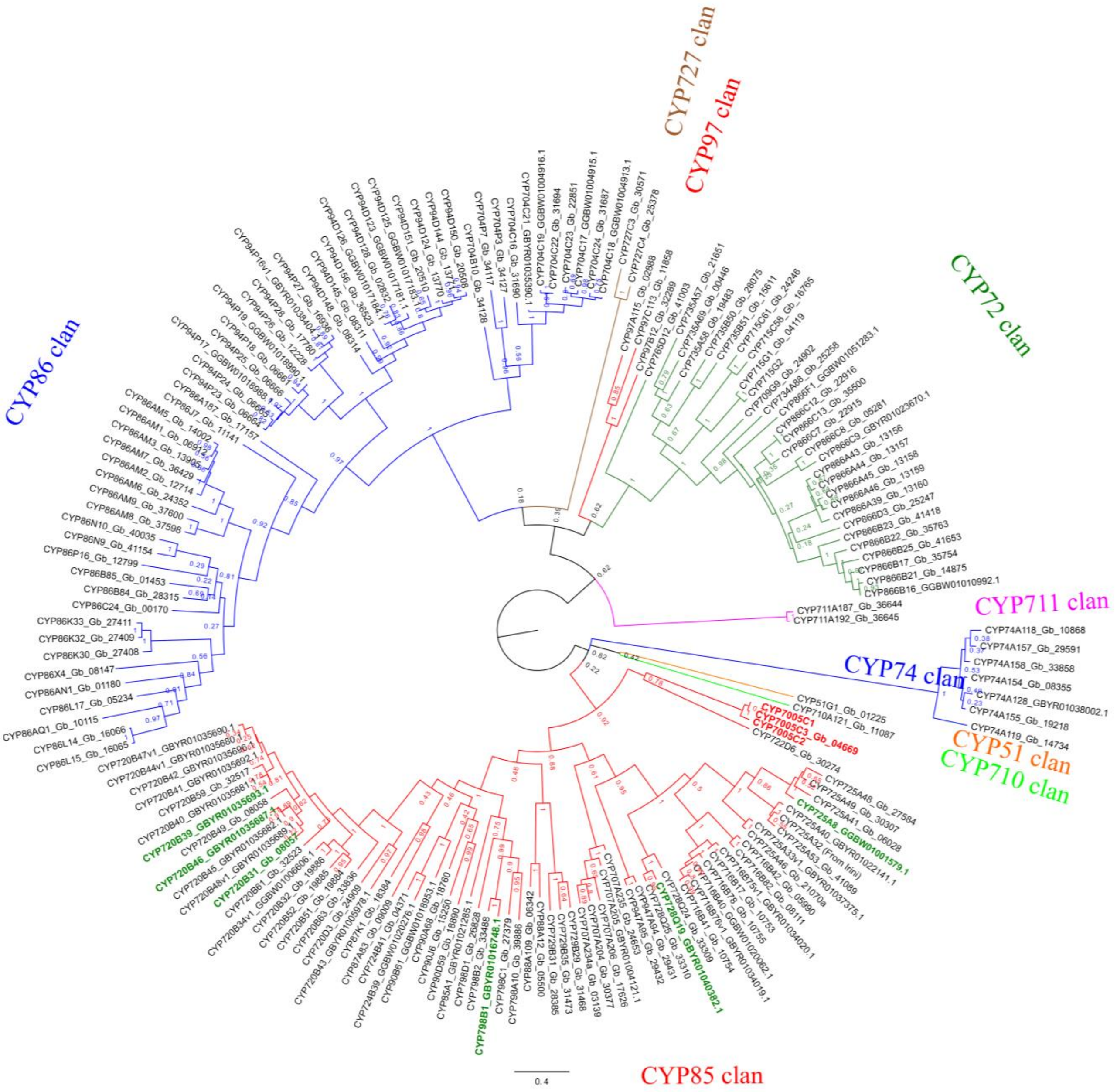

Supplementary Figure 12. Evolutionary relations of the *Ginkgo biloba* CYP enzymes with exception of those belonging to the CYP71 clan. This analysis involved 173 amino acid sequences. There was a total of 442 positions in the final dataset. The tree is drawn to scale, with branch lengths measured in the number of substitutions per site. In red-bold font are marked the CYPs found in the Biosynthetic Gene Cluster, and in green-bold font are marked the CYPs co-expressed with *Gb*LPS.

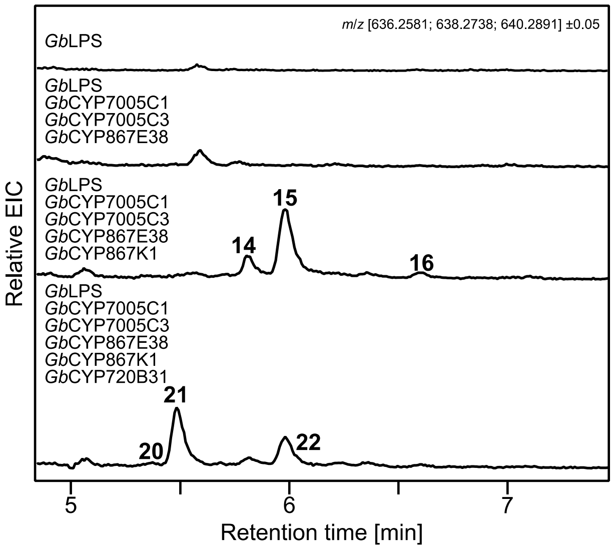

Supplementary Fig 13. Putative glutathione conjugated products 14-16 and 20-22 observed in tobacco expressing the enzymes shown on the top of the panel. a, LC-HRMS chromatograms showing products **19-24** when expressing *Gb*CYP867K1 and *Gb*CYP720B31 combinations. The chromatograms shown are EICs of *m/z* values indicated in the top right corner. EIC: Extracted Ion Chromatograms.

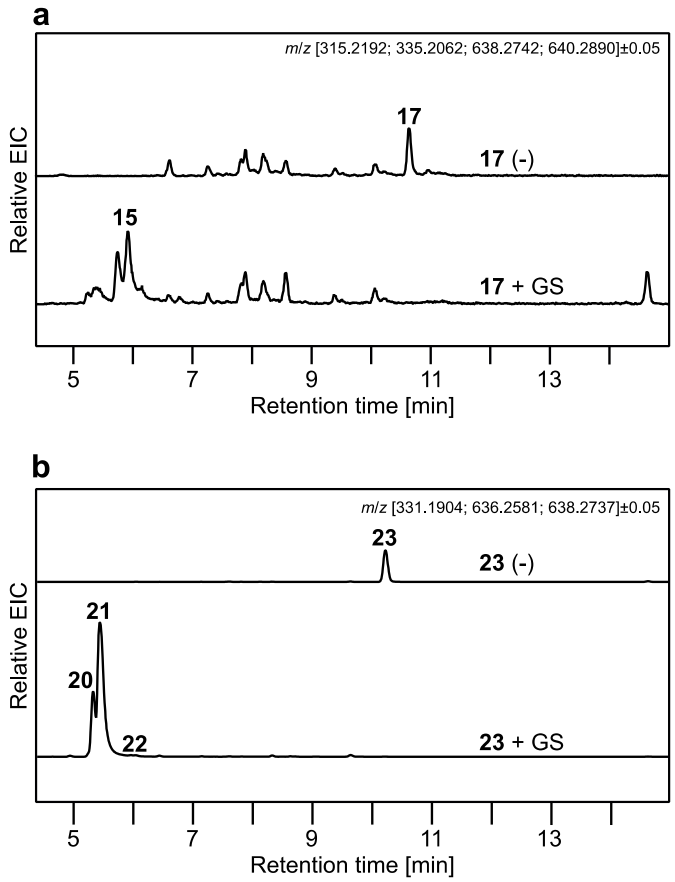

Supplementary Figure 14. *In vitro* chemical reaction of glutathione (GS) with 17 and 23. a, LC-HRMS extracted ion chromatograms (EIC) showing how reaction of **17** with glutathione leads to formation of **15** *in vitro*. **b**, LC-HRMS extracted ion chromatograms (EIC) showing how the reaction of **23** with glutathione leads to formation of **20-22**.

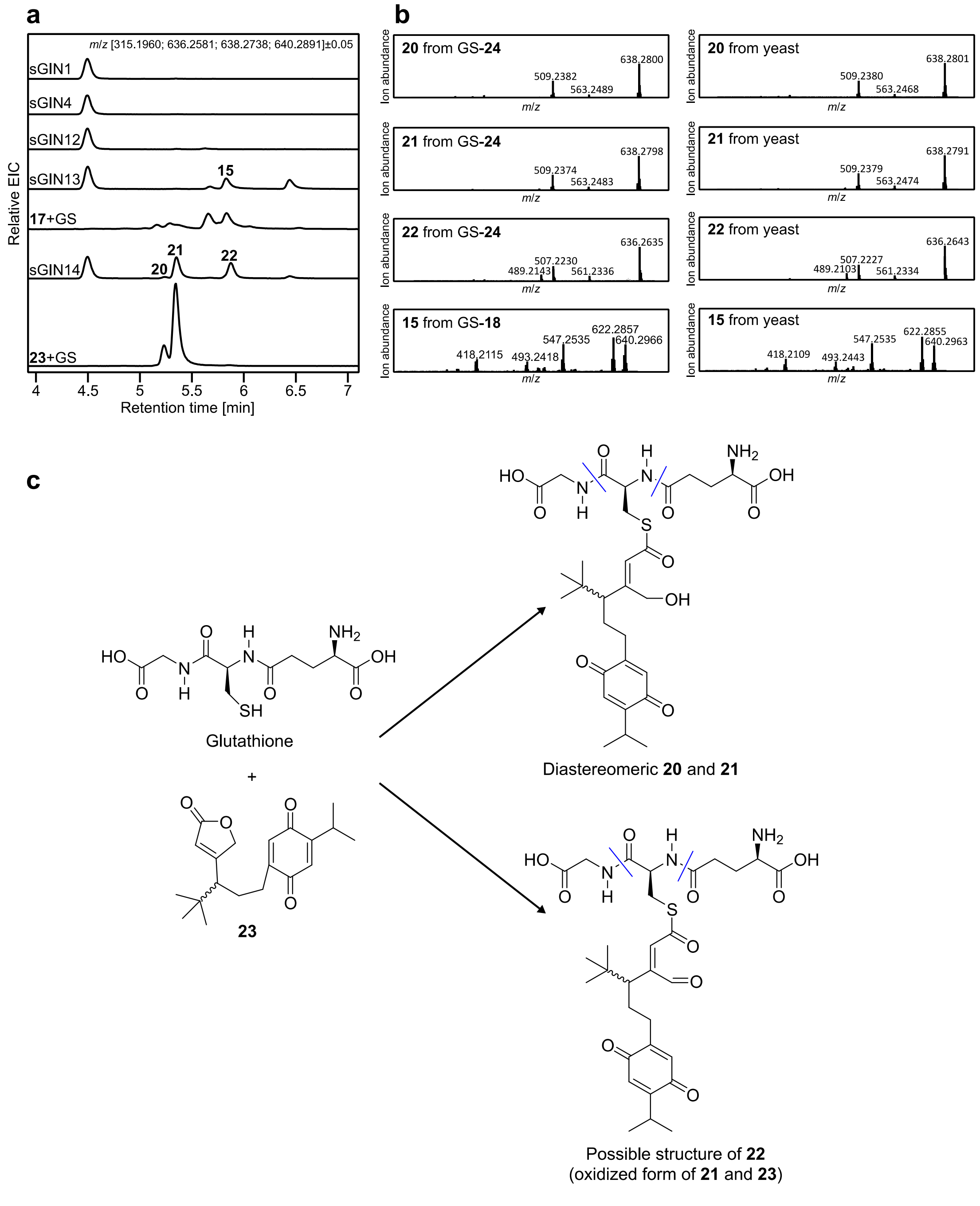

Supplementary Figure 15. Glutathione (GS) adducts produced in yeast and from reacting 17 and 23 with glutathione. a, LC-HRMS extracted ion chromatograms (EIC) showing putative glutathione molecules produced in yeast and how they overlap with **17** and **23** reaction products with glutathione. **b**, MS^2 patterns of overlapping molecules, showing that **15** and **20-22** produced in yeast are the same as **17** and **23** reacting with glutathione *in vitro*. **c**, proposed structures of **20-22** based on MS^2 patterns.

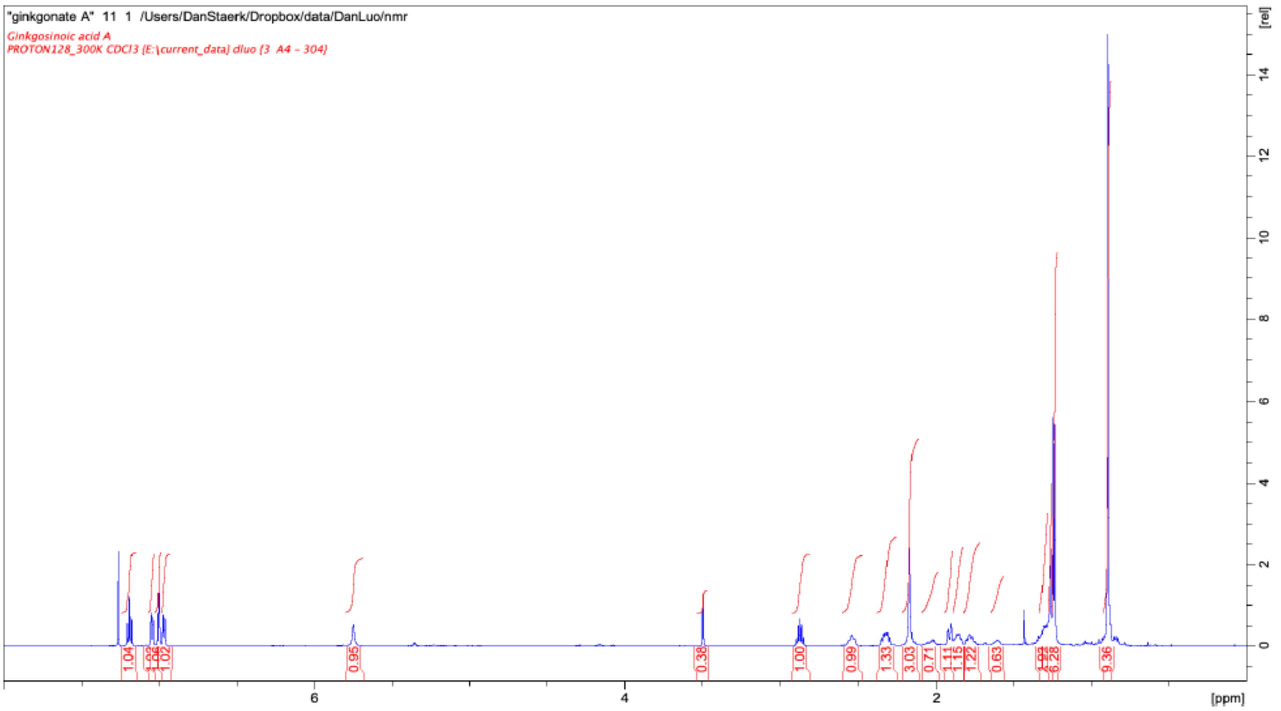

Supplementary Figure 16. 1H NMR spectrum (600 MHz) of ginkgosinoic acid A (**7**) in chloroform-*d*.

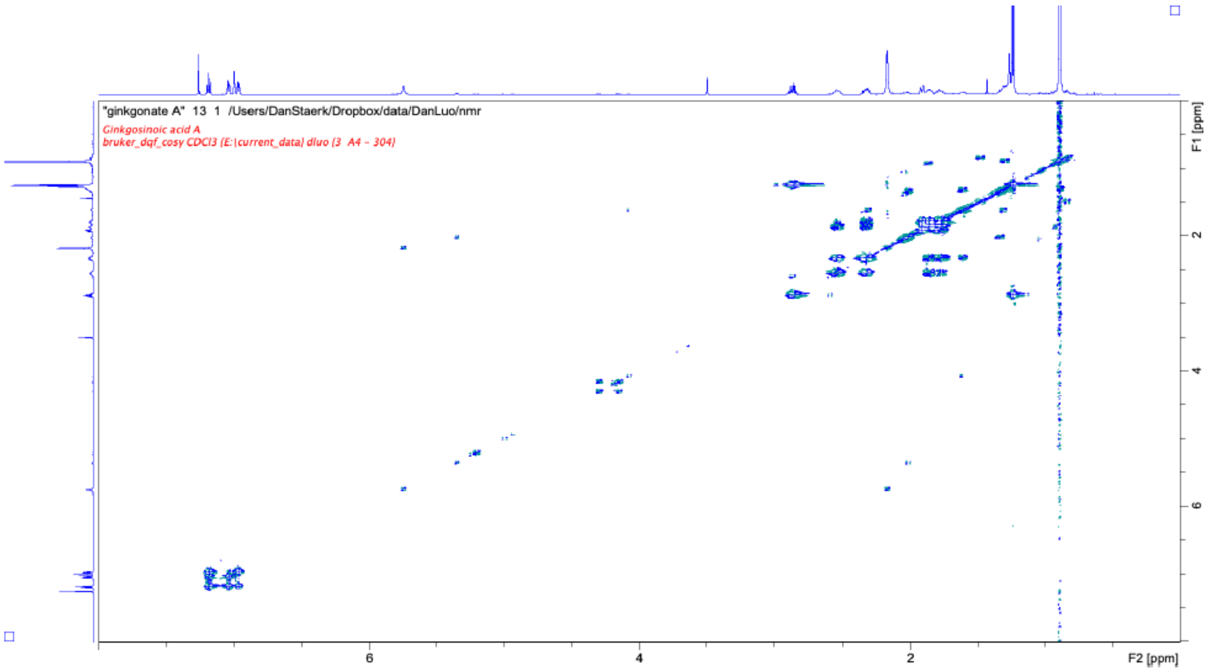

Supplementary Figure 17. COSY spectrum of ginkgosinoic acid A (**7**) in chloroform-*d*.

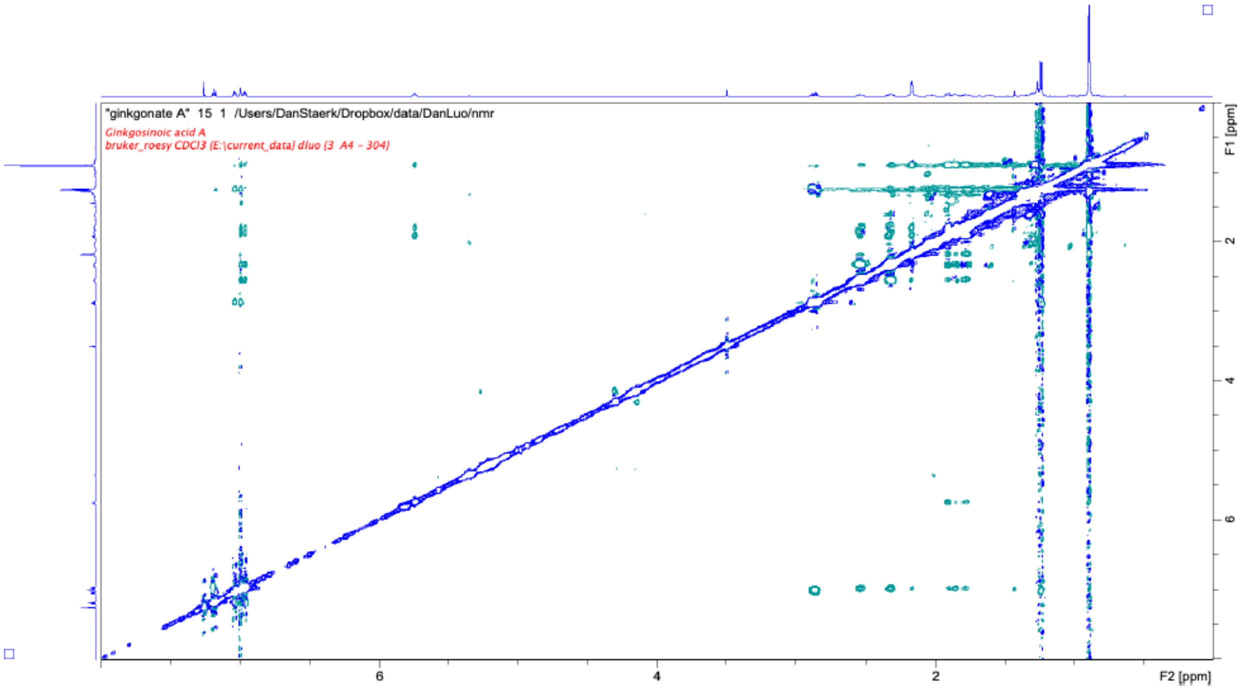

Supplementary Figure 18. ROESY spectrum of ginkgosinoic acid A (**7**) in chloroform-*d*.

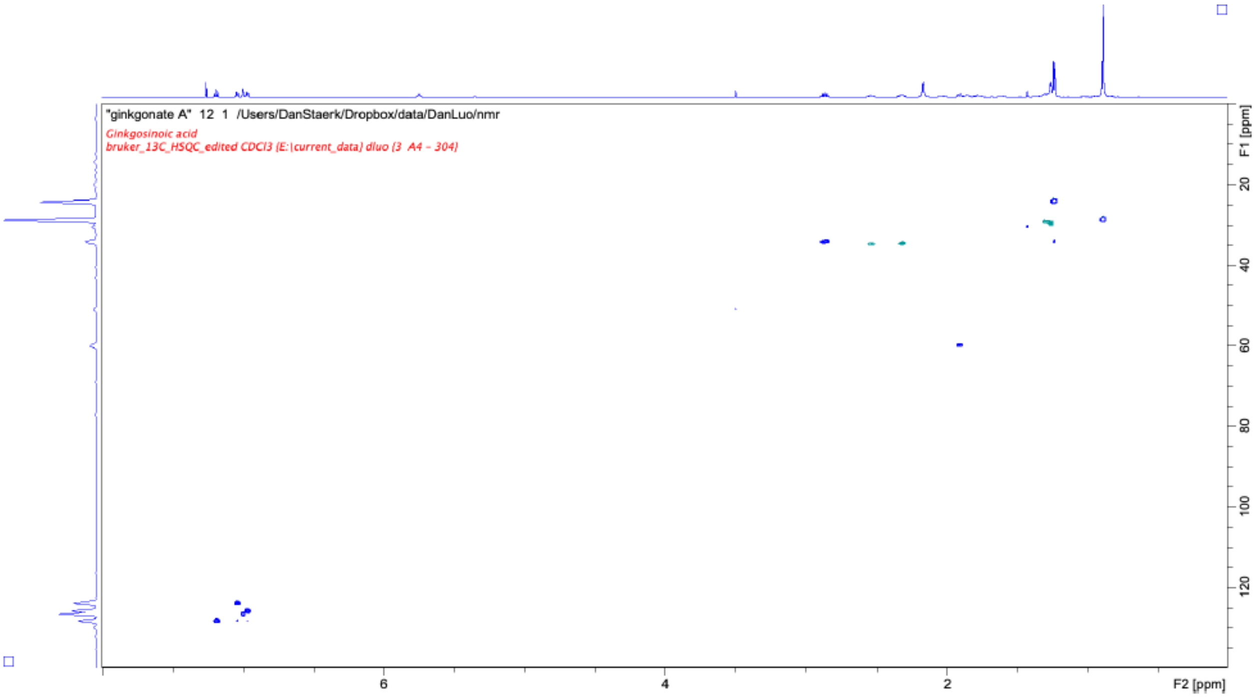

Supplementary Figure 19. Multiplicity-edited HSQC spectrum of ginkgosinoic acid A (**7**) in chloroform-*d*.

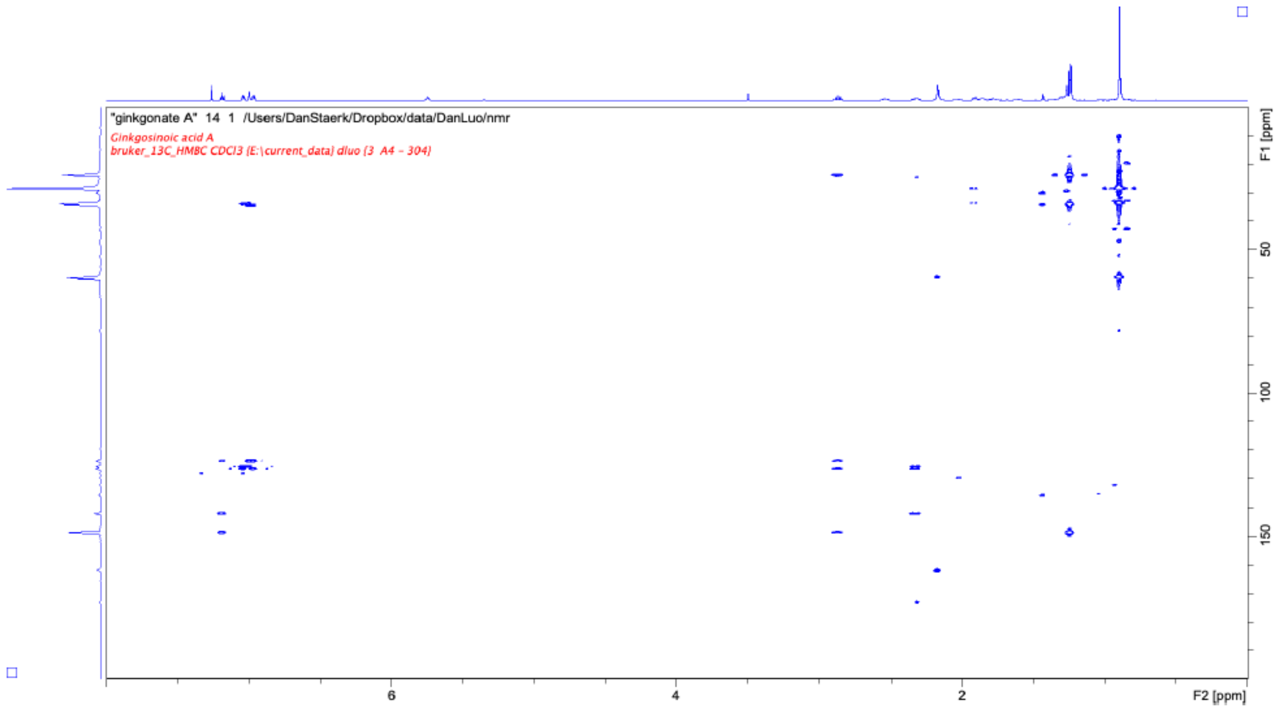

Supplementary Figure 20. HMBC spectrum of ginkgosinoic acid A (**7**) in chloroform-*d*.

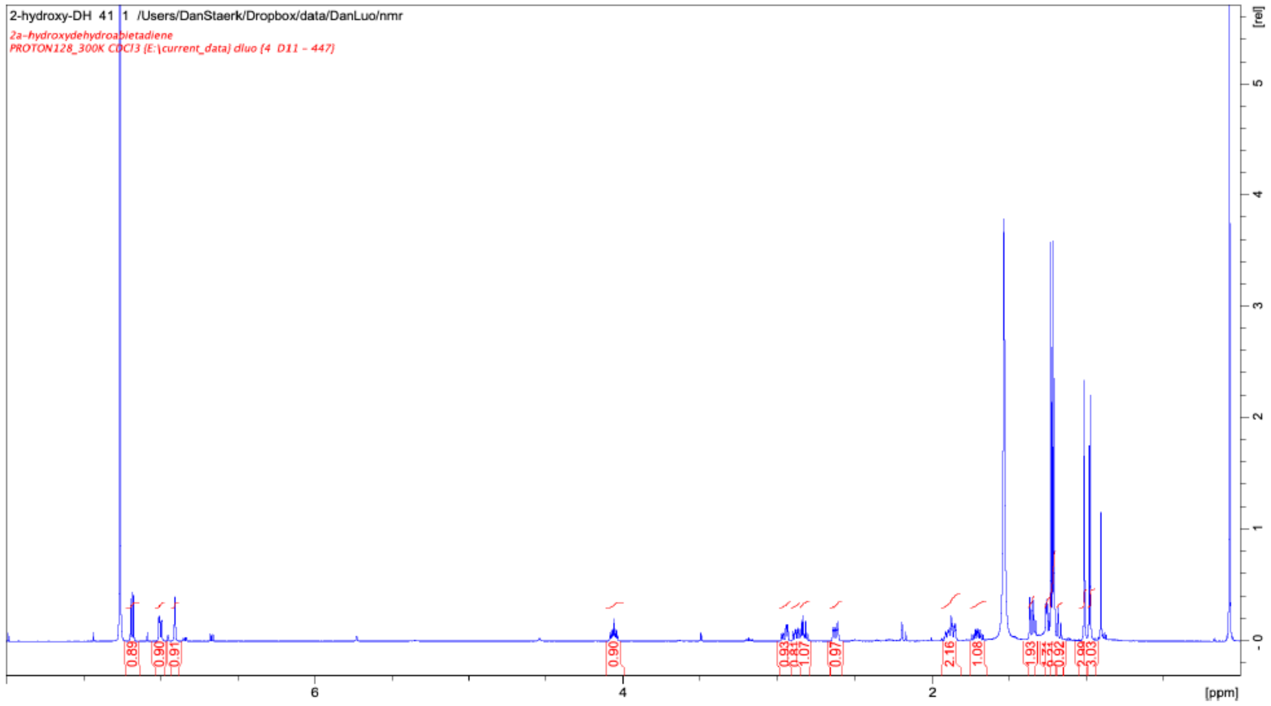

Supplementary Figure 21. 1H NMR spectrum (600 MHz) of 2a-hydroxydehydroabietadiene (**8**) in chloroform-*d*.

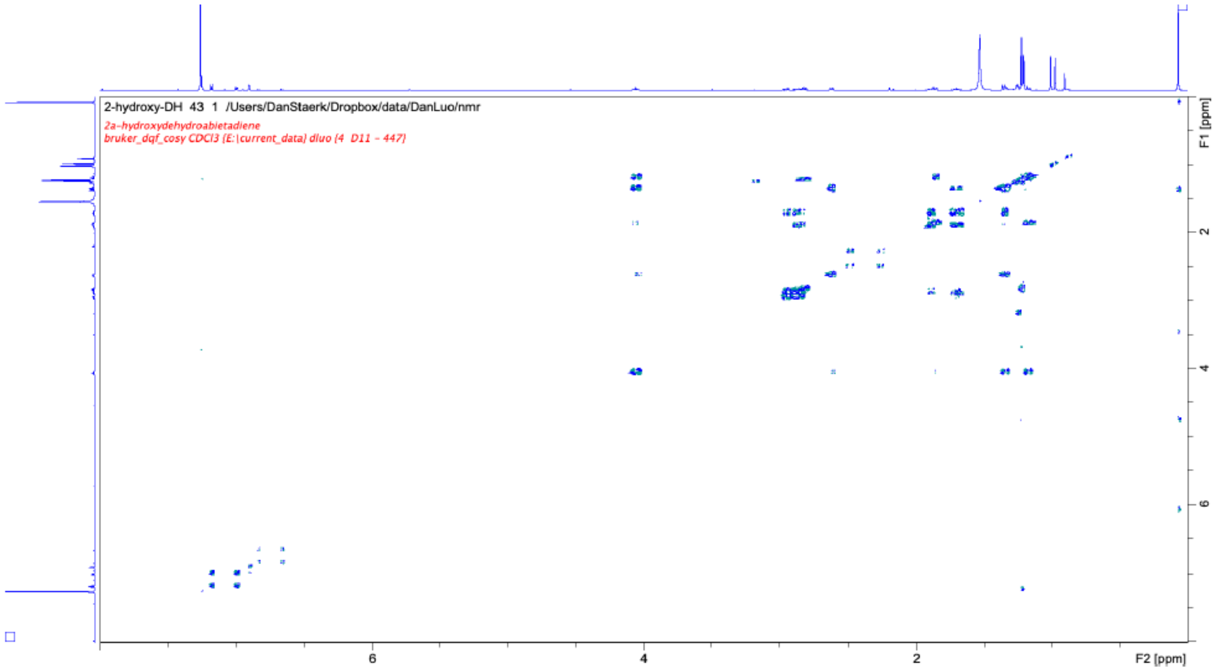

Supplementary Figure 22. COSY spectrum of 2a-hydroxydehydroabietadiene (**8**) in chloroform-*d*.

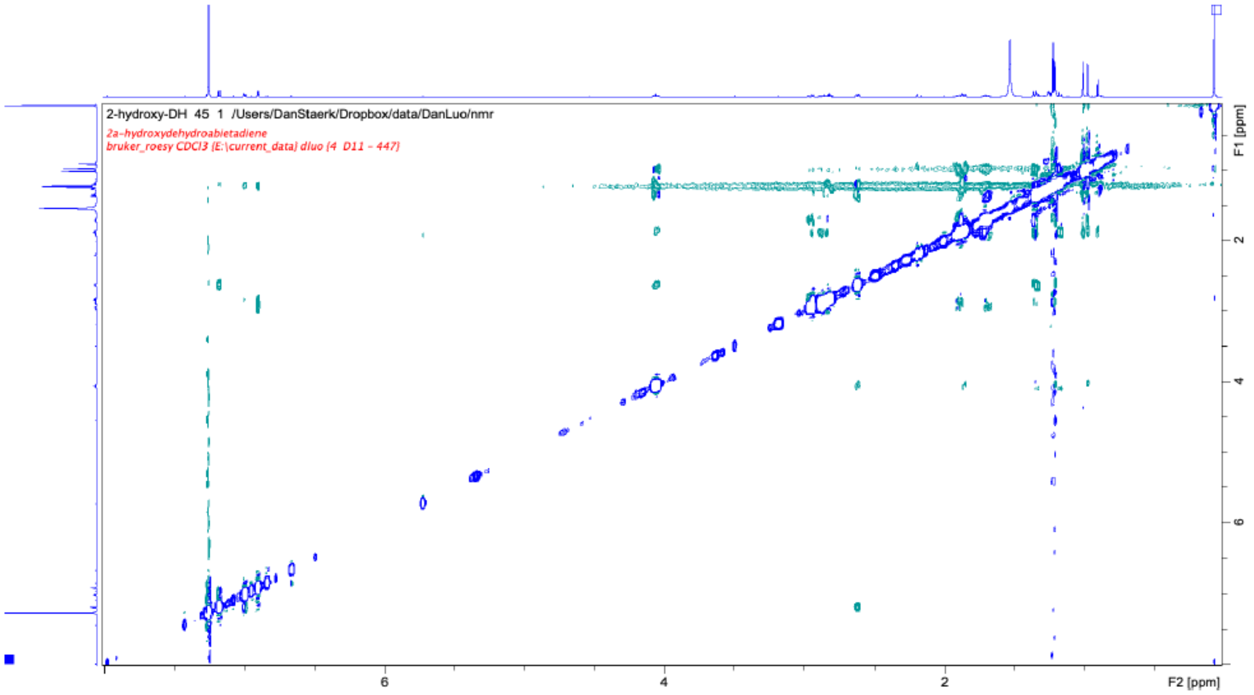

Supplementary Figure 23. ROESY spectrum of 2a-hydroxydehydroabietadiene (**8**) in chloroform-*d*.

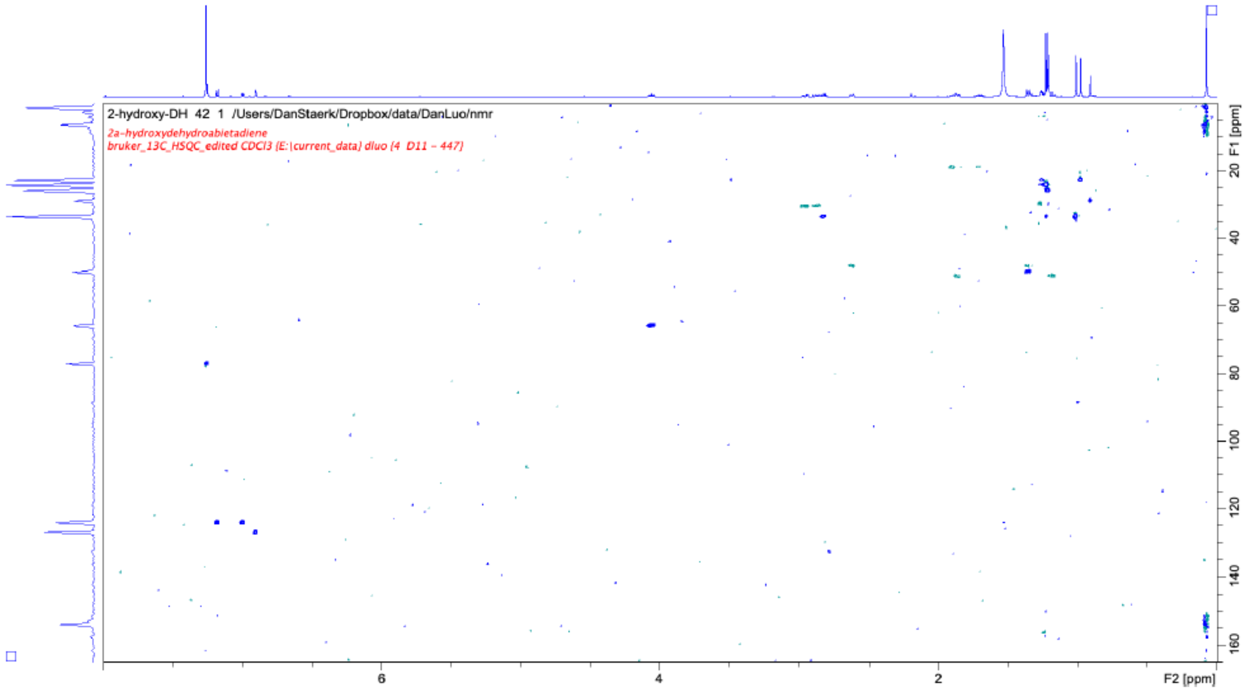

Supplementary Figure 24. Multiplicity-edited HSQC spectrum of 2a-hydroxydehydroabietadiene (**8**) in chloroform-*d*.

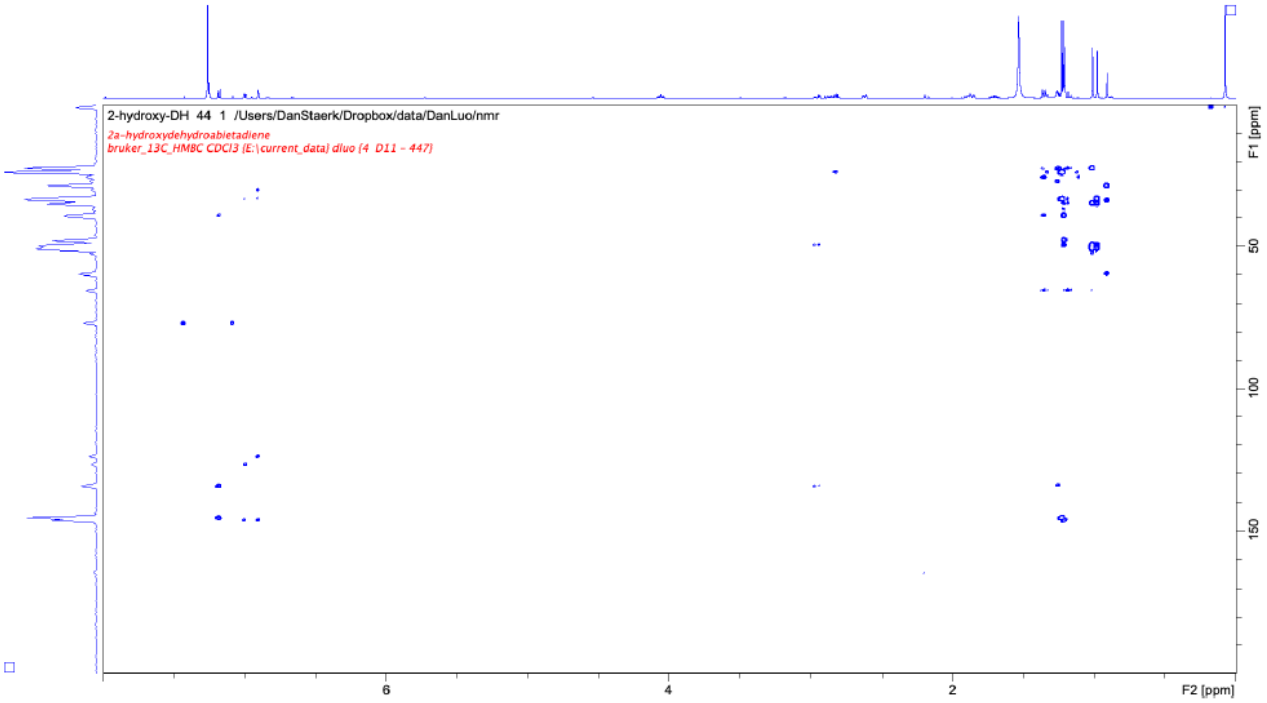

Supplementary Figure 25. HMBC spectrum of 2a-hydroxydehydroabietadiene (**8**) in chloroform-*d*.

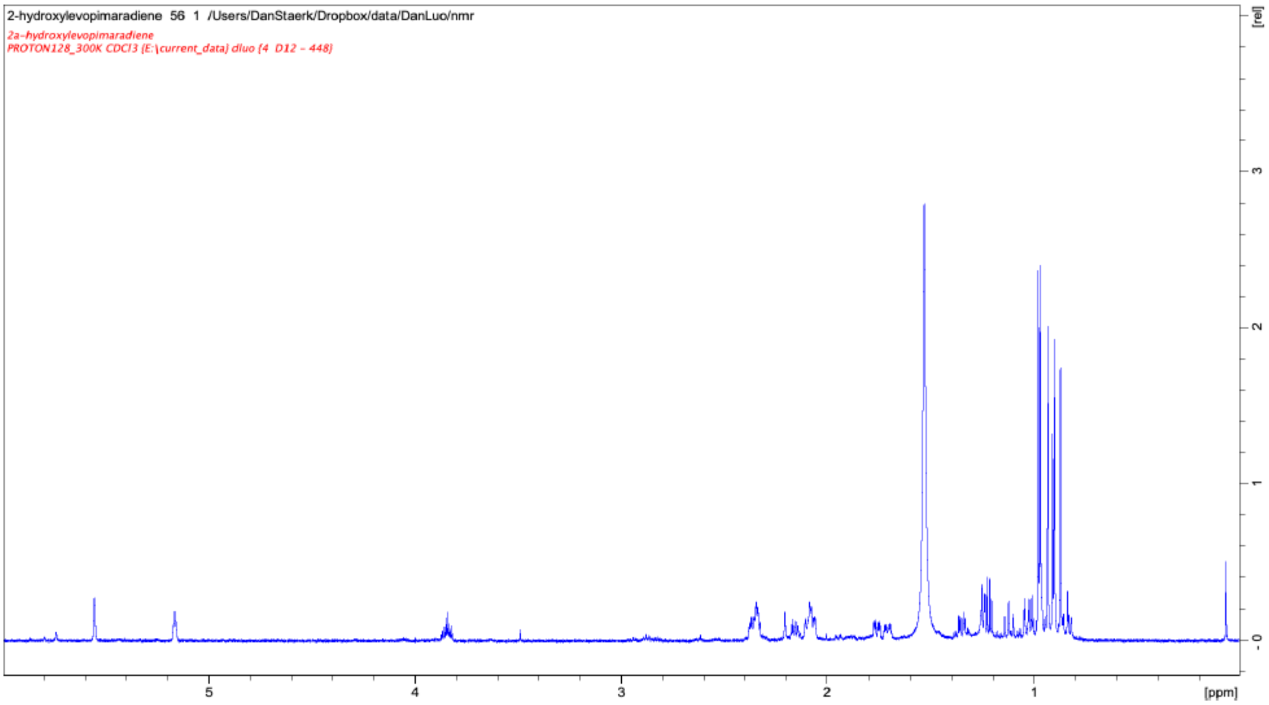

Supplementary Figure 26. 1H NMR spectrum (600 MHz) of 2a-hydroxylevopimaradiene (**9**) in chloroform-*d*.

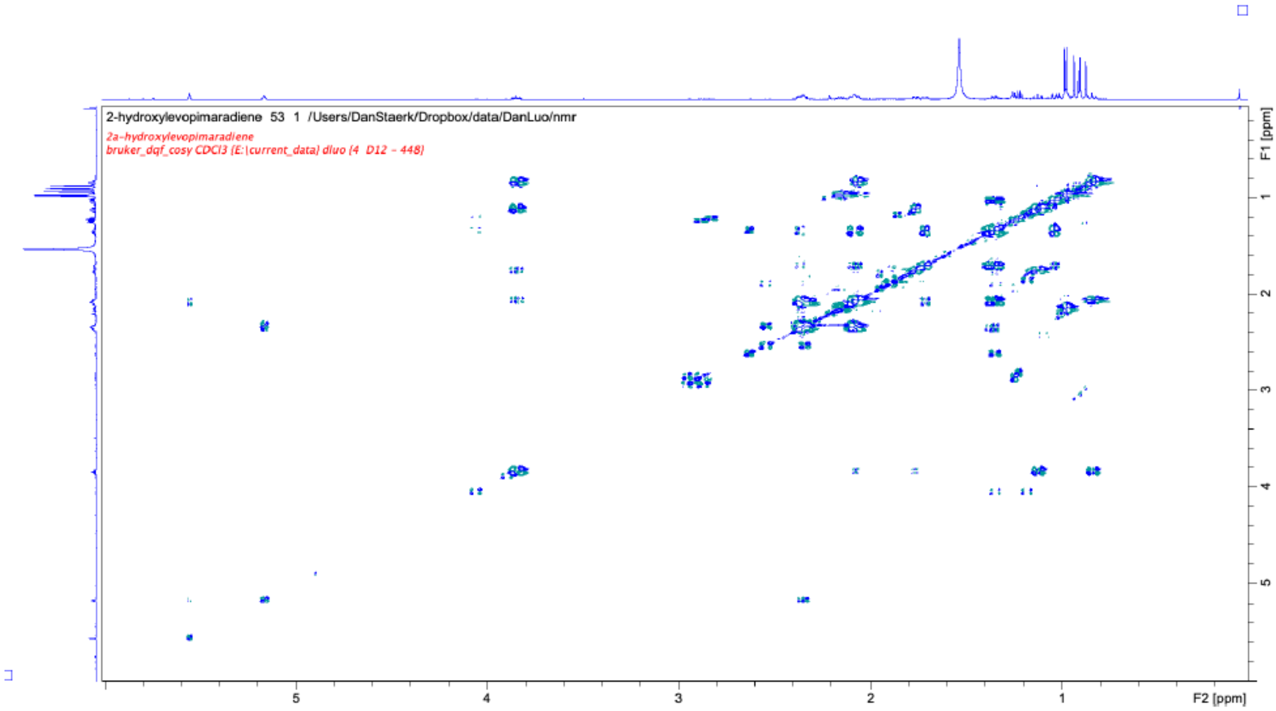

Supplementary Figure 27. COSY spectrum of 2a-hydroxylevopimaradiene (**9**) in chloroform-*d*.

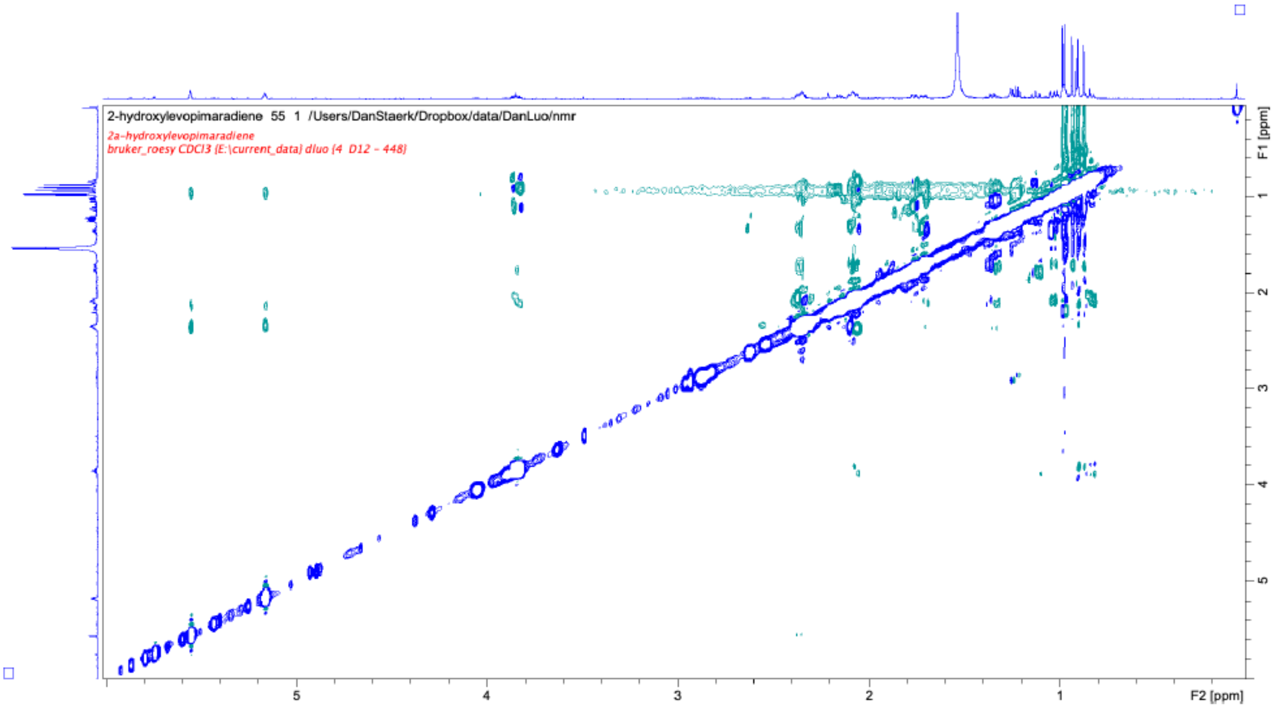

Supplementary Figure 28. ROESY spectrum of 2a-hydroxylevopimaradiene (**9**) in chloroform-*d*.

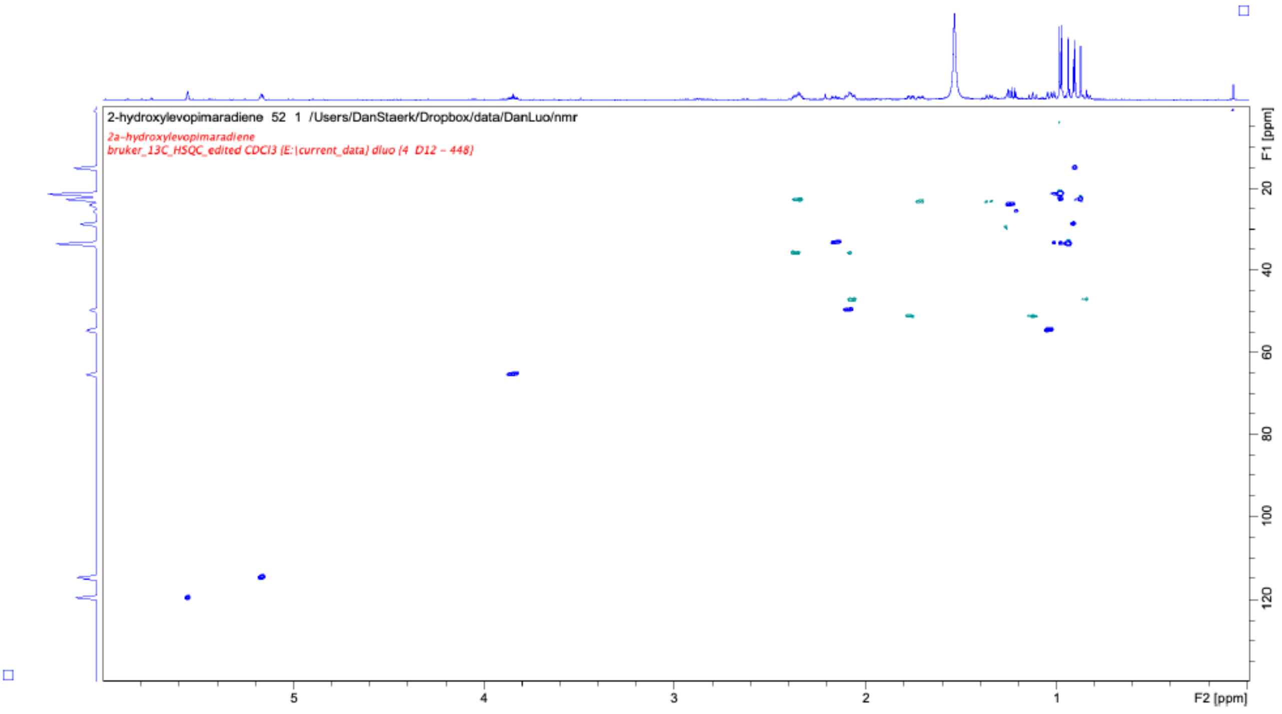

Supplementary Figure 29. Multiplicity-edited HSQC spectrum of 2a-hydroxylevopimaradiene (**9**) in chloroform-*d*.

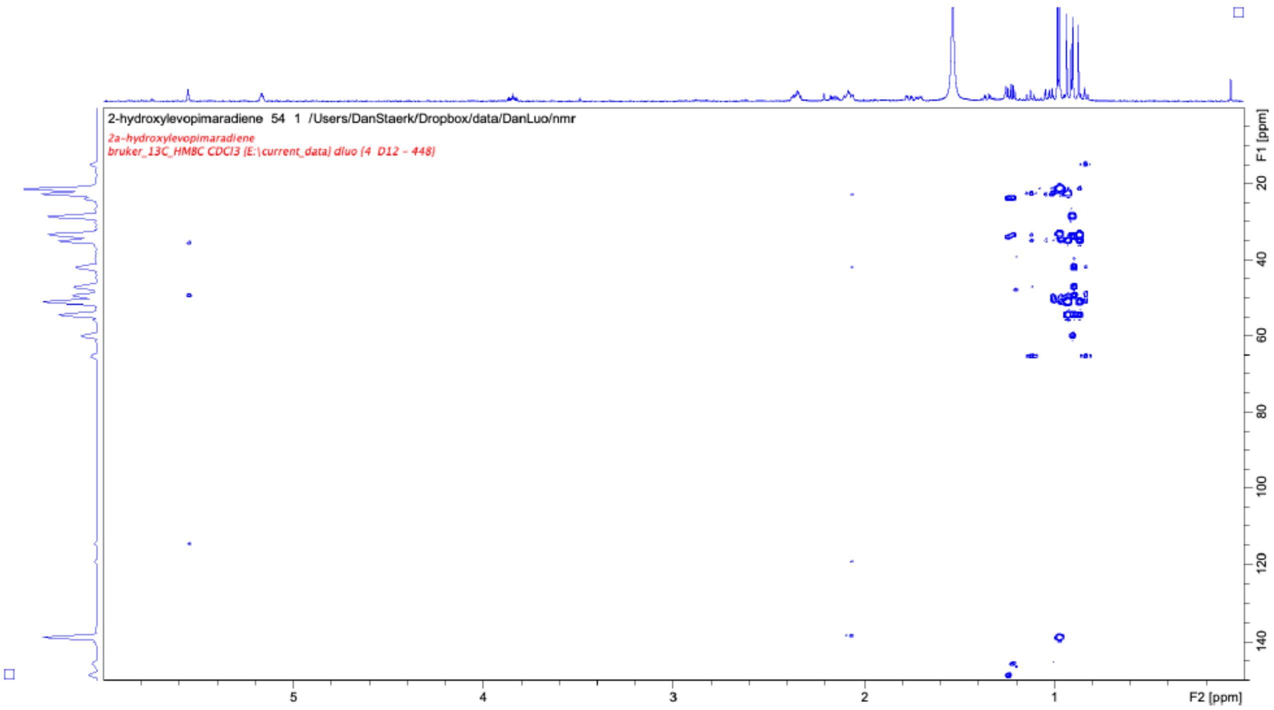

Supplementary Figure 30. HMBC spectrum of 2a-hydroxylevopimaradiene (**9**) in chloroform-*d*.

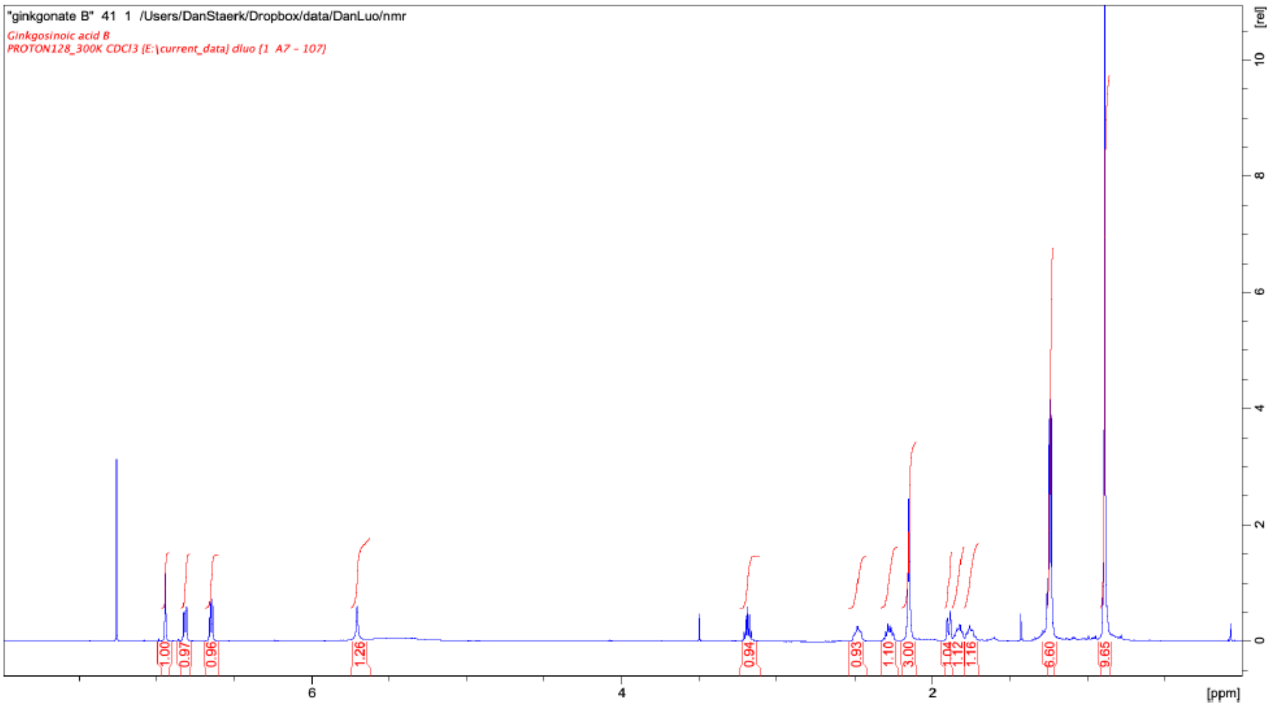

Supplementary Figure 31. 1H NMR spectrum (600 MHz) of ginkgosinoic acid B (**11**) in chloroform-*d*.

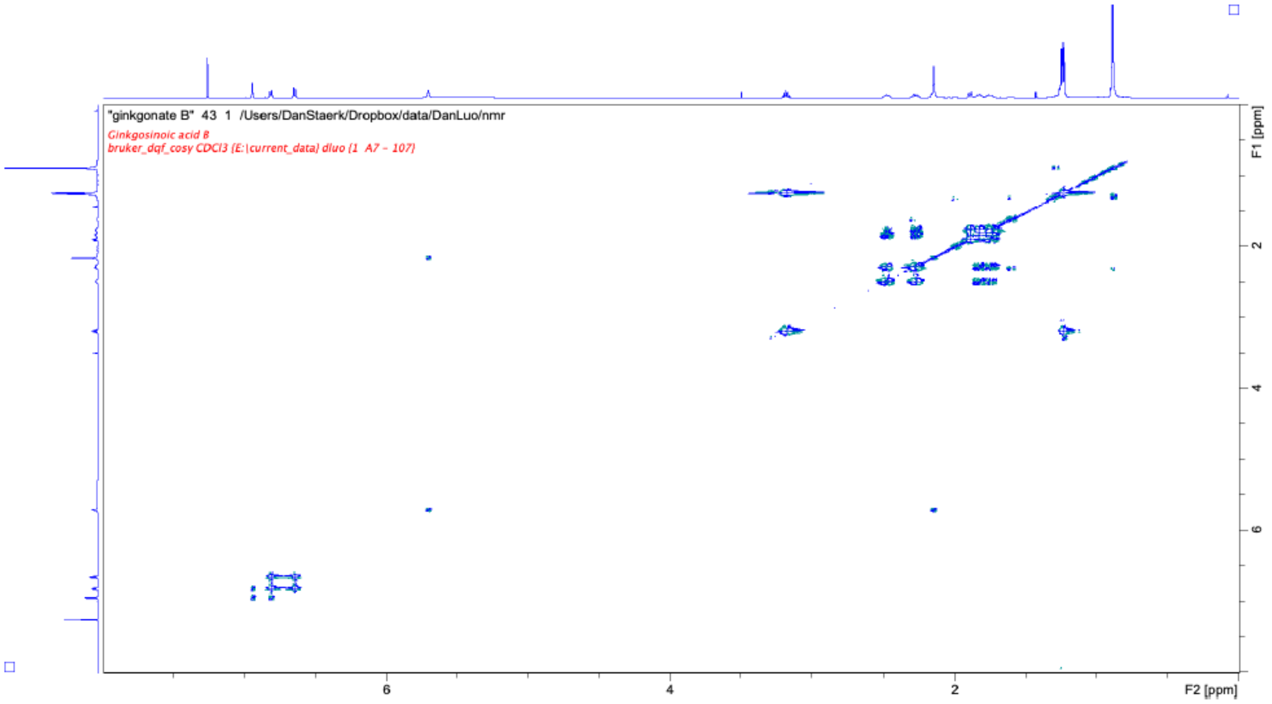

Supplementary Figure 32. COSY spectrum of ginkgosinoic acid B (**11**) in chloroform-*d*.

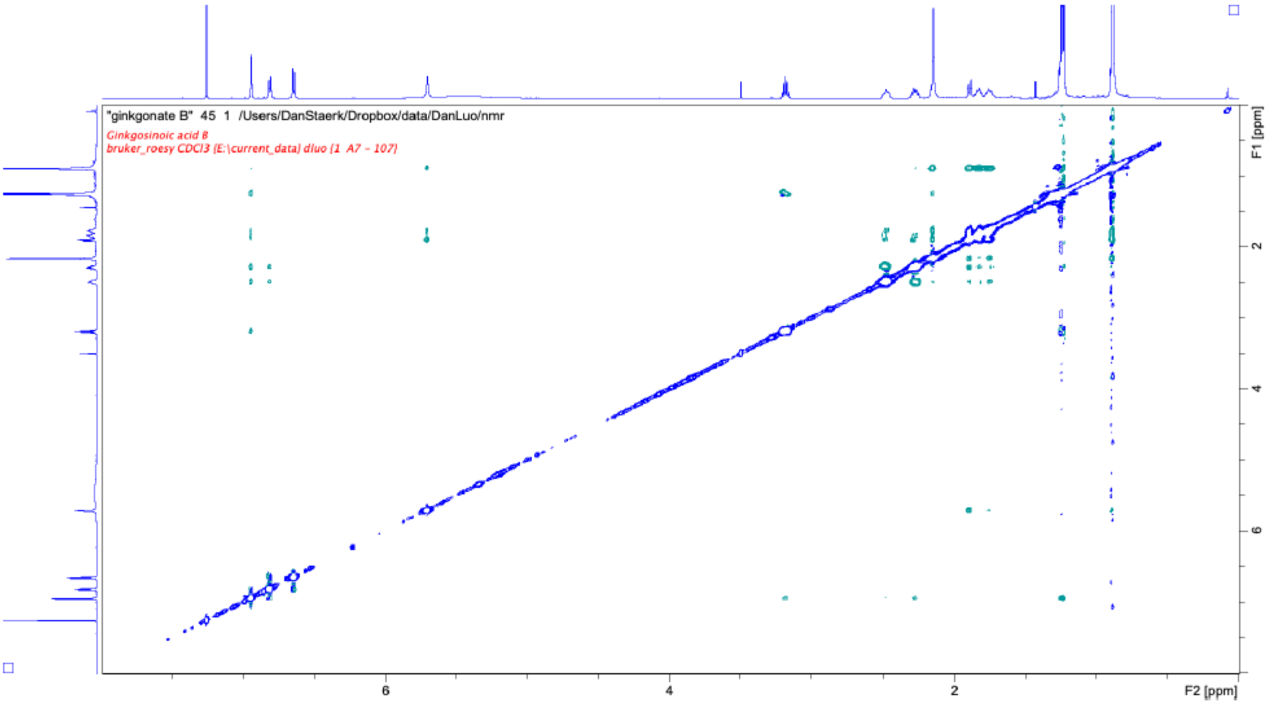

Supplementary Figure 33. ROESY spectrum of ginkgosinoic acid B (**11**) in chloroform-*d*.

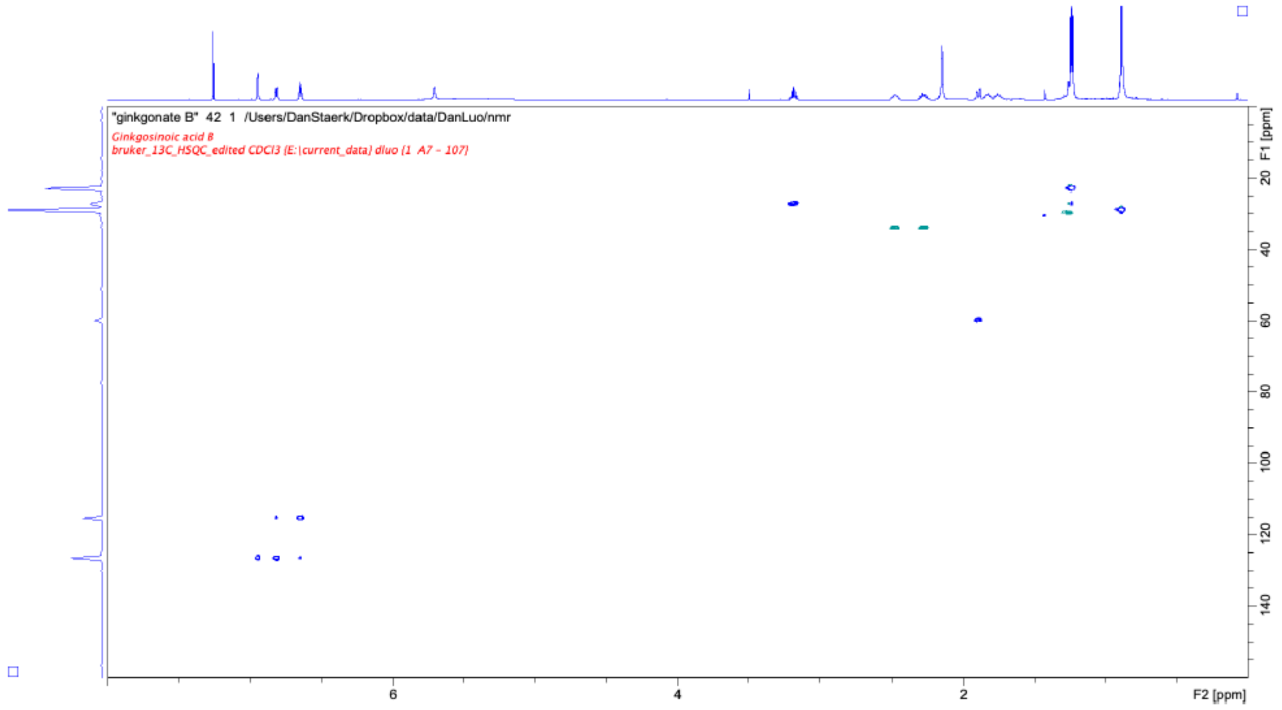

Supplementary Figure 34. Multiplicity-edited HSQC spectrum of ginkgosinoic acid B (**11**) in chloroform-*d*.

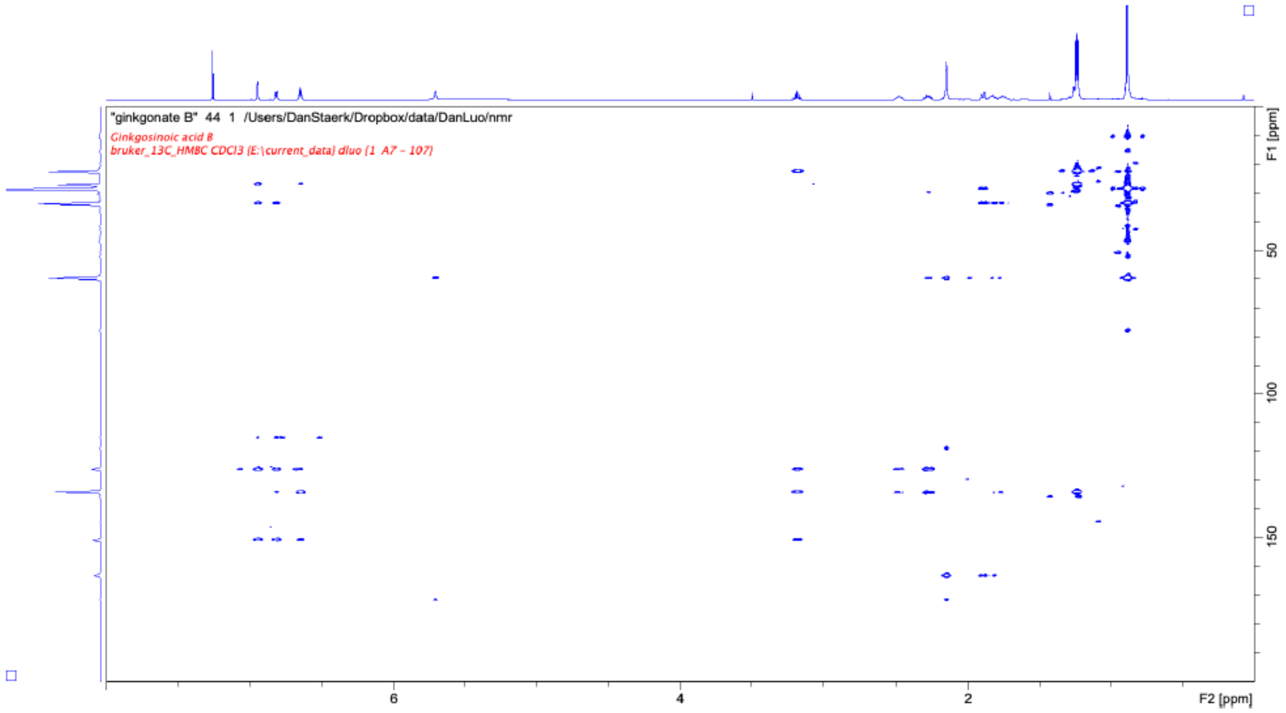

Supplementary Figure 35. HMBC spectrum of ginkgosinoic acid B (**11**) in chloroform-*d*.

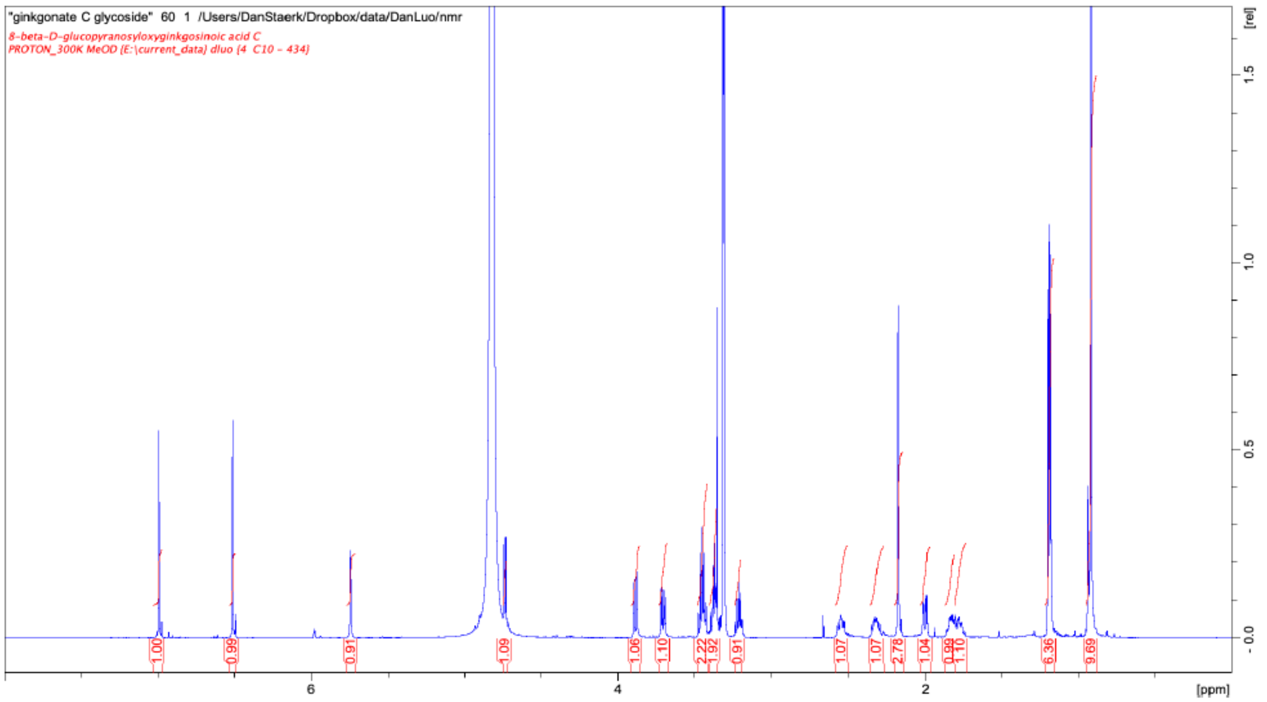

Supplementary Figure 36. ^{1}H NMR spectrum (600 MHz) of 8-b-D-glucopyranosyloxyginkgosinoic acid C (**13d**) in methanol-*d*$_4$.

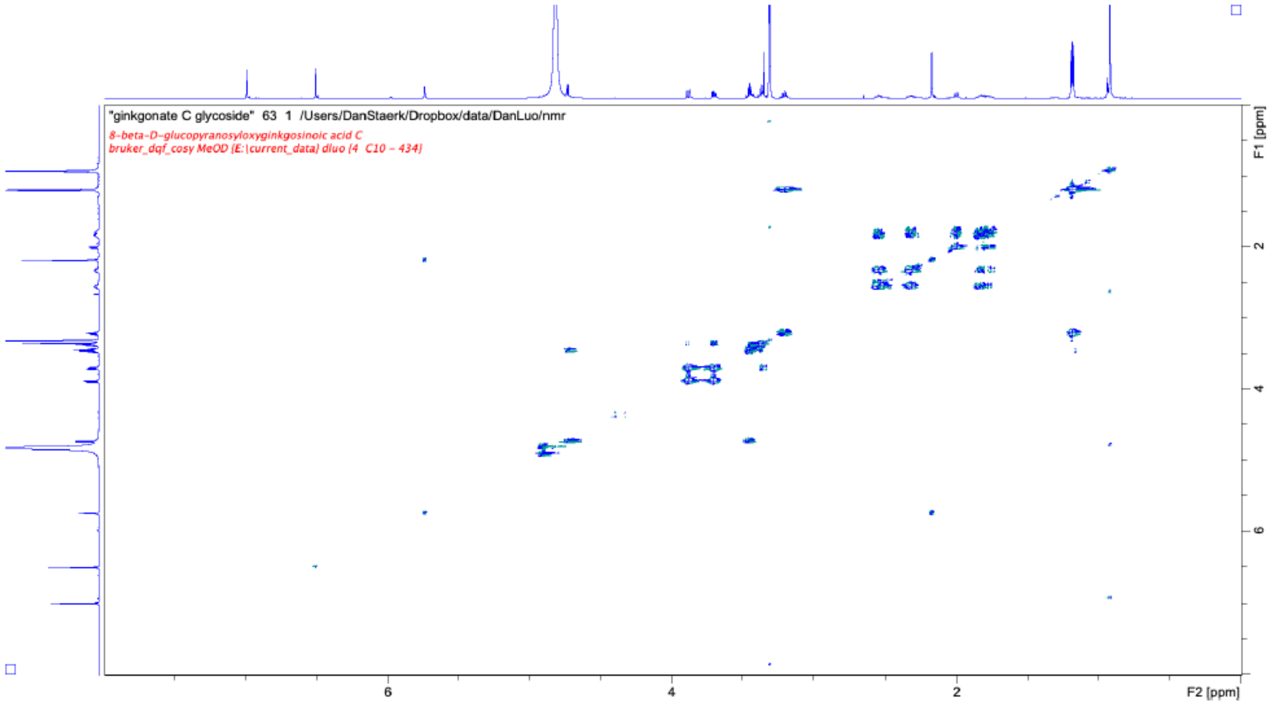

Supplementary Figure 37. COSY spectrum of 8-b-D-glucopyranosyloxyginkgosinoic acid C (**13d**) in methanol-*d*$_4$.

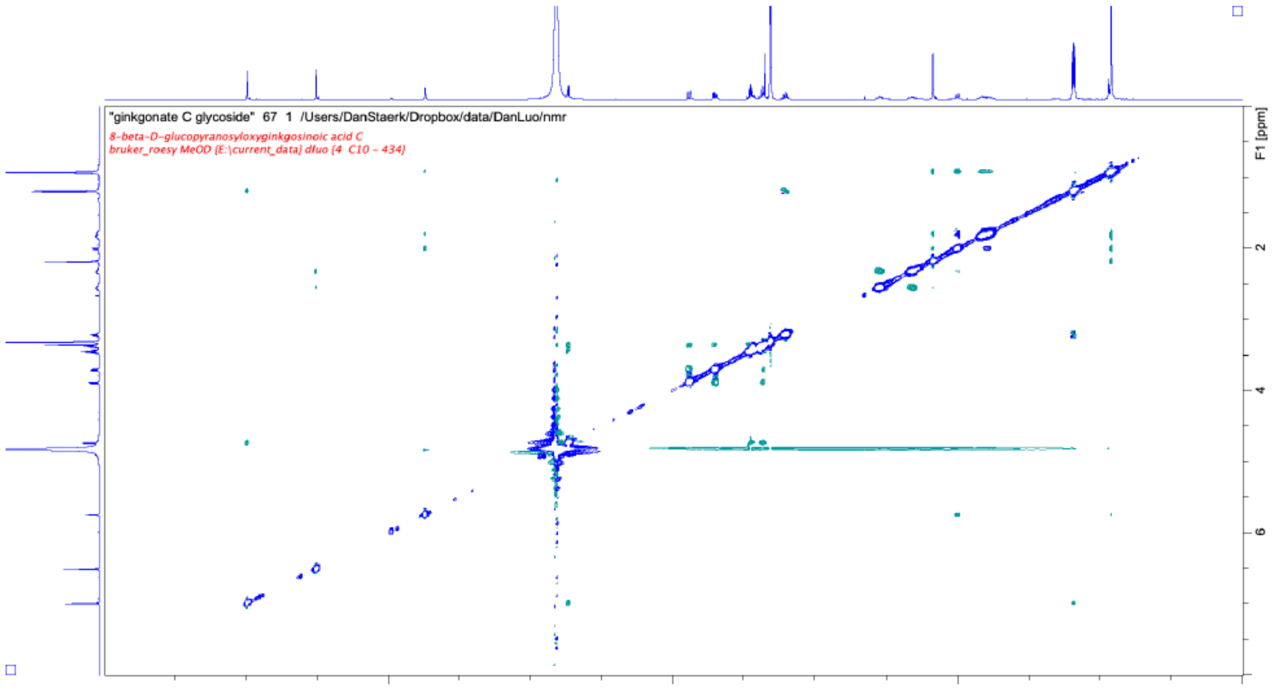

Supplementary Figure 38. ROESY spectrum of 8-b-D-glucopyranosyloxyginkgosinoic acid C (**13d**) in methanol-*d*4.

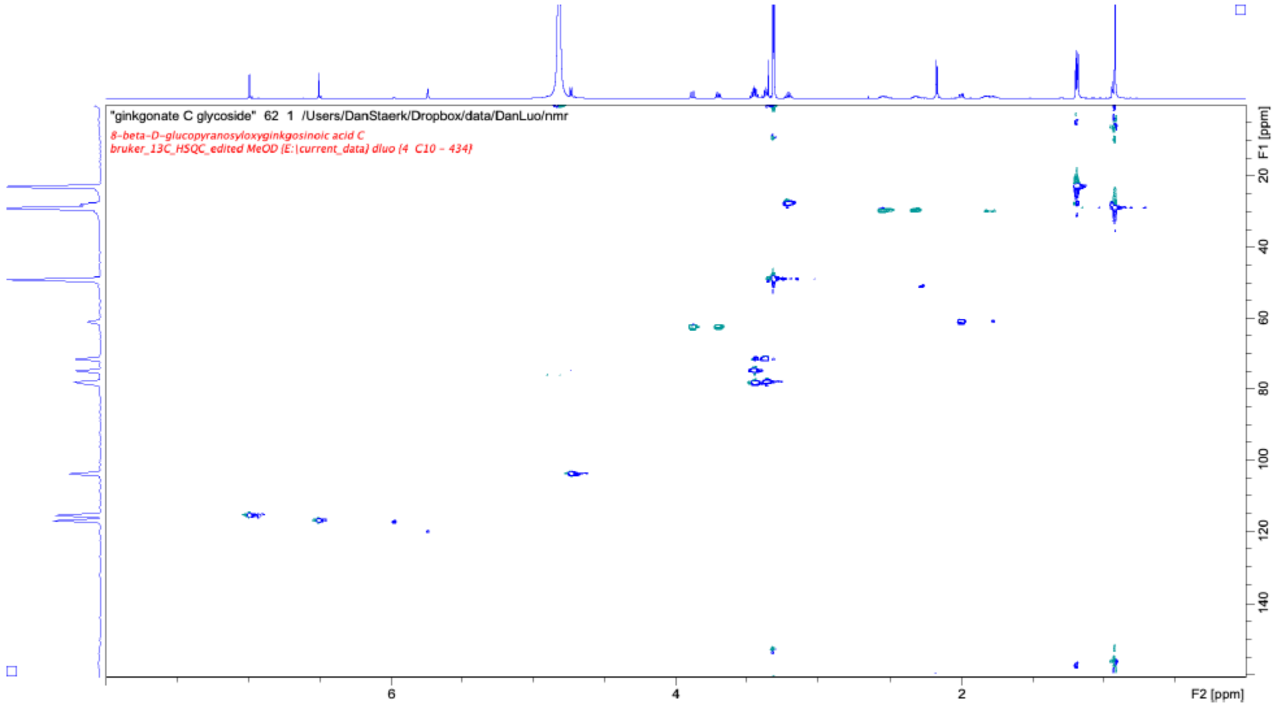

Supplementary Figure 39. Multiplicity-edited HSQC spectrum of 8-b-D-glucopyranosyloxyginkgosinoic acid C (**13d**) in methanol-*d*4.

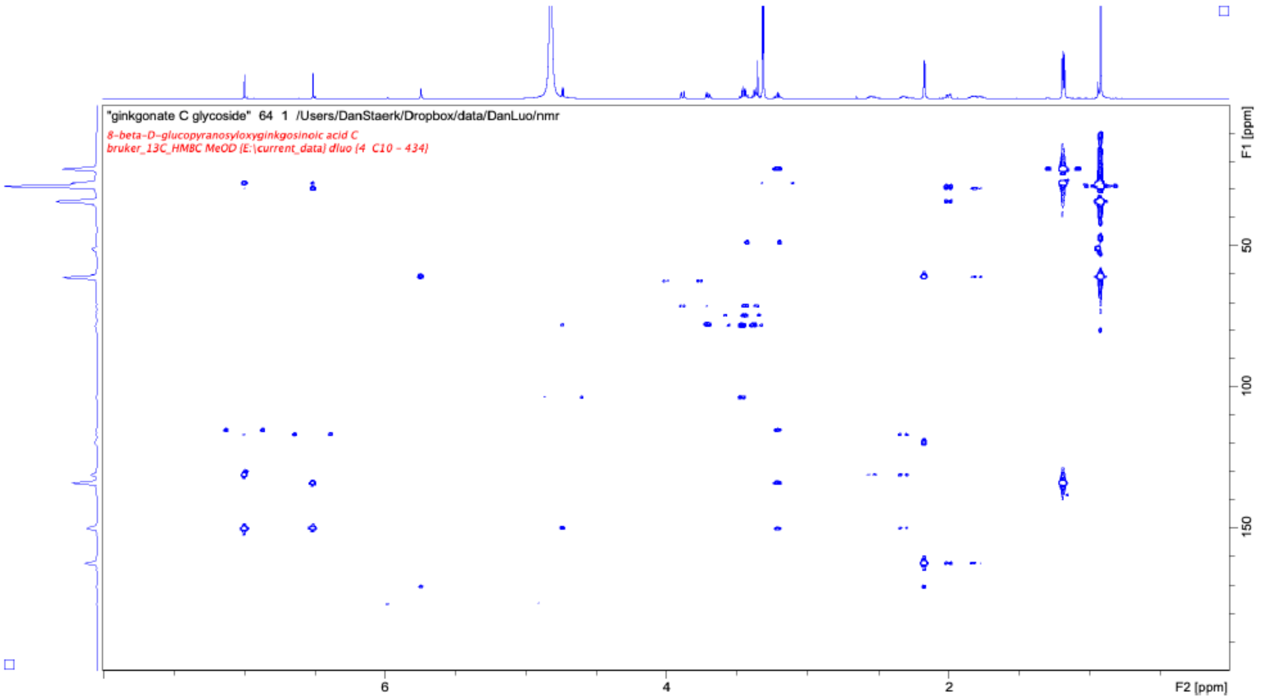

Supplementary Figure 40. HMBC spectrum of 8-b-D-glucopyranosyloxyginkgosinoic acid C (**13d**) in methanol-*d*4.

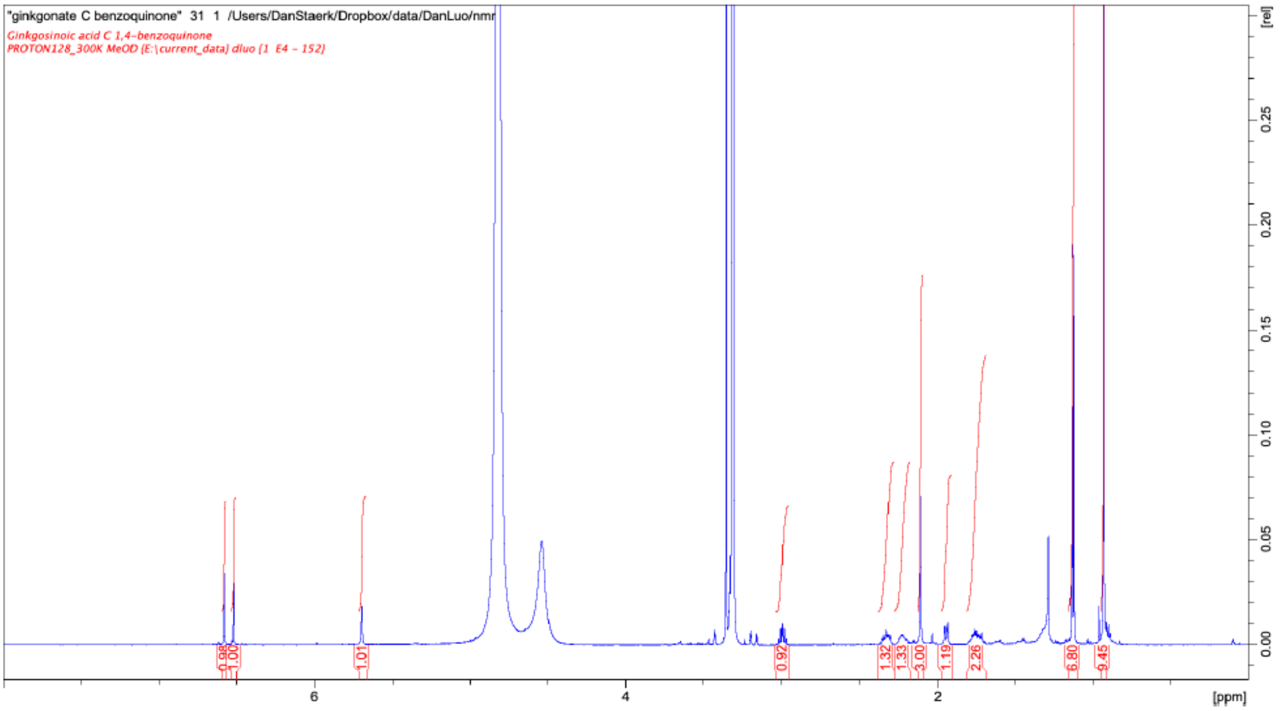

Supplementary Figure 41. 1H NMR spectrum (600 MHz) of ginkgosinoic acid C 1,4-benzoquinone (**17**) in methanol-*d*4.

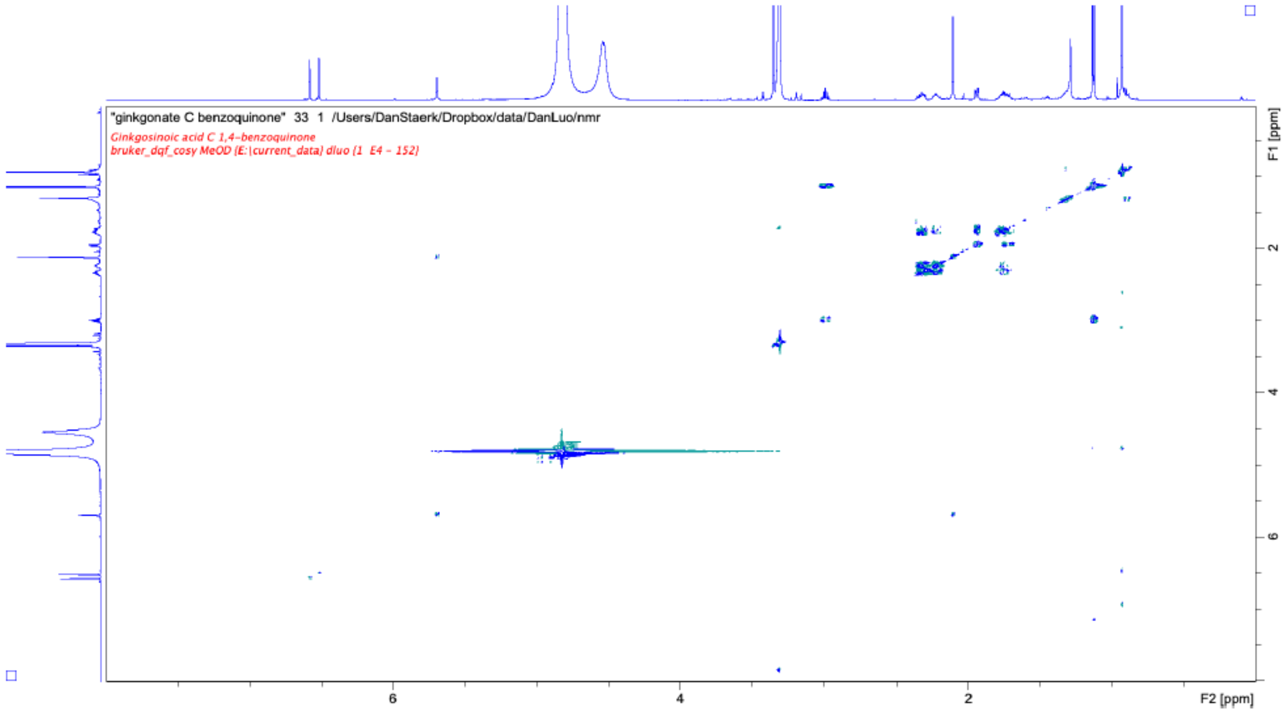

Supplementary Figure 42. COSY spectrum of ginkgosinoic acid C 1,4-benzoquinone (**17**) in methanol-d_4.

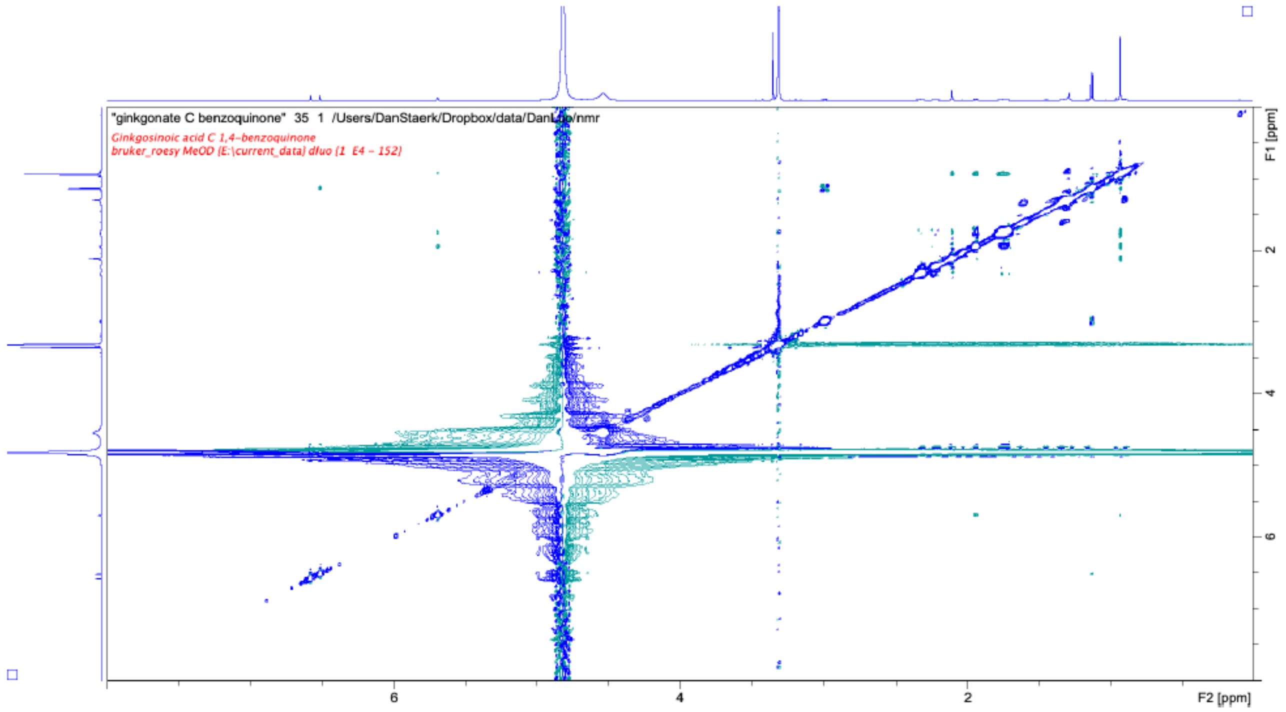

Supplementary Figure 43. ROESY spectrum of ginkgosinoic acid C 1,4-benzoquinone (**17**) in methanol-d_4.

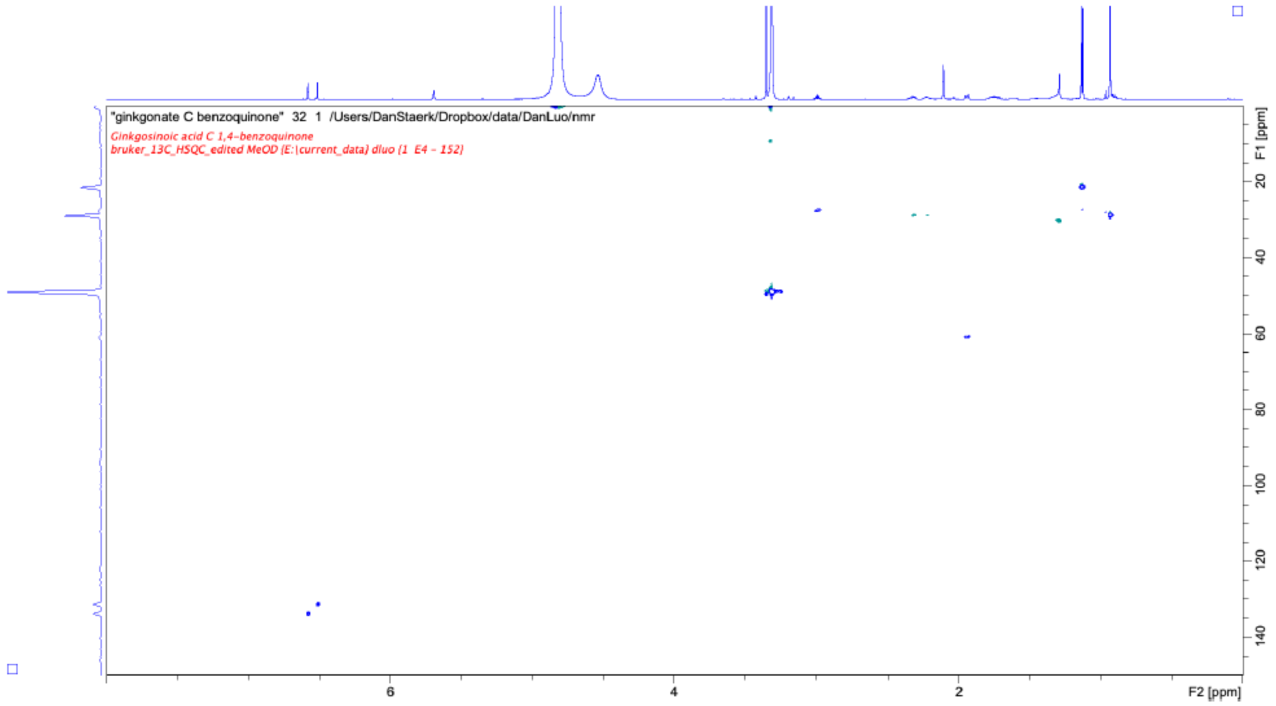

Supplementary Figure 44. Multiplicity-edited HSQC spectrum of ginkgosinoic acid C 1,4-benzoquinone (**17**) in methanol-*d*4.

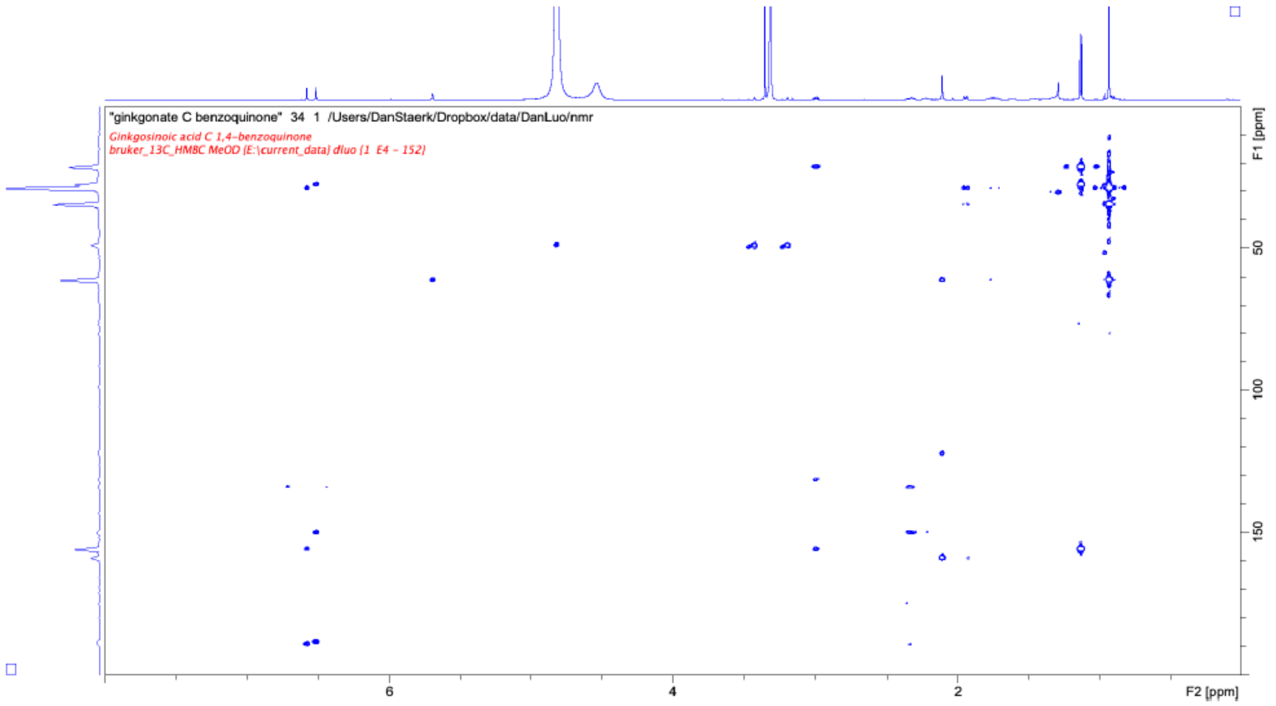

Supplementary Figure 45. HMBC spectrum of ginkgosinoic acid C 1,4-benzoquinone (**17**) in methanol-*d*4.

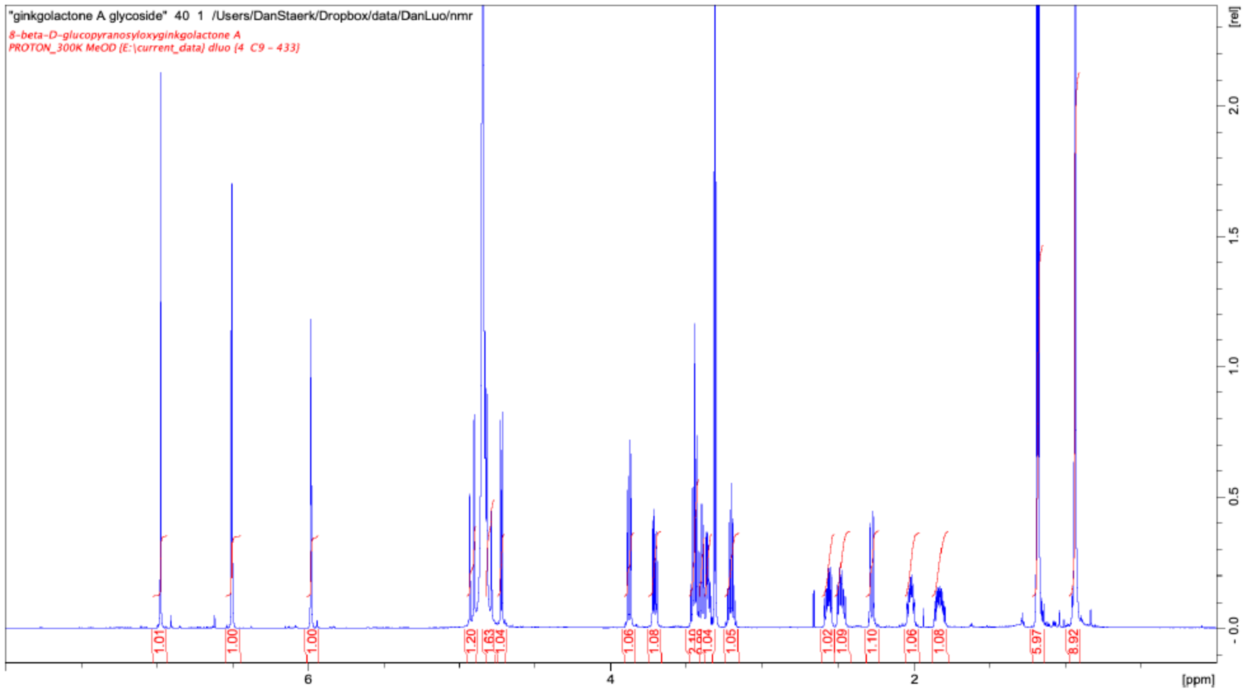

Supplementary Figure 46. ^{1}H NMR spectrum (600 MHz) of 8-b-D-glucopyranosyloxyginkgolactone A (**18c**) in methanol-d_4.

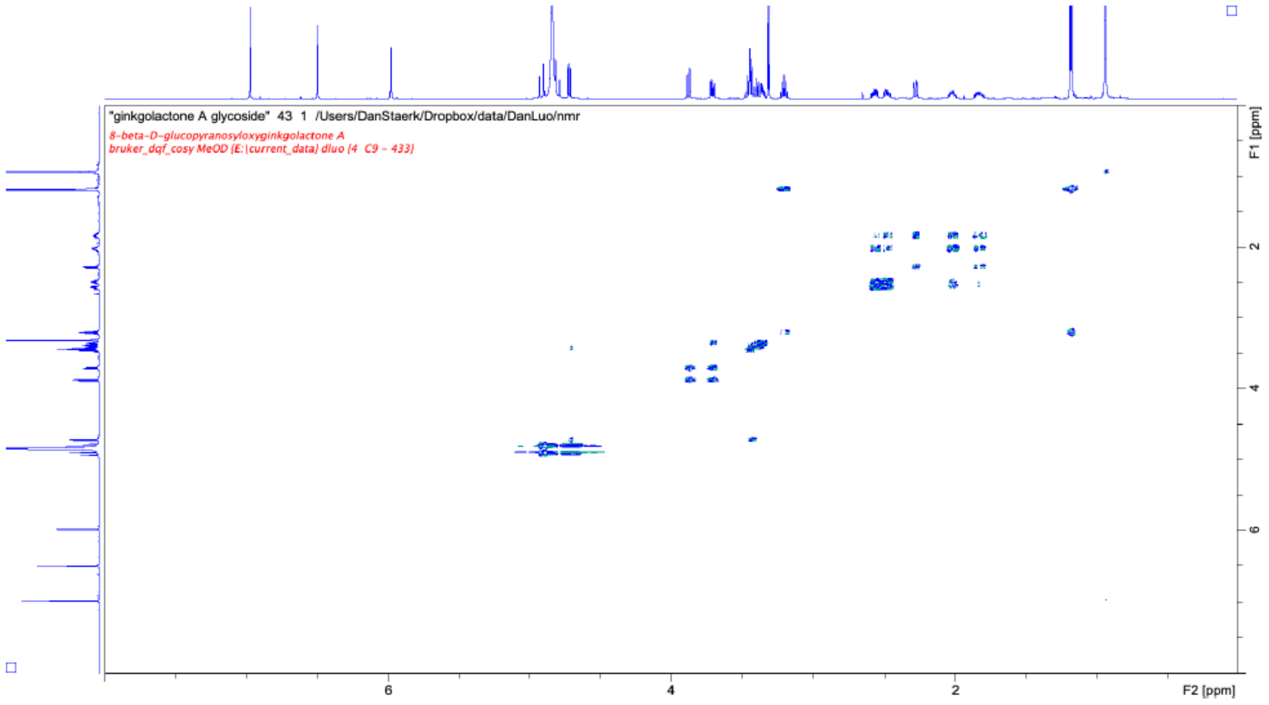

Supplementary Figure 47. COSY spectrum of 8-b-D-glucopyranosyloxyginkgolactone A (**18c**) in methanol-d_4.

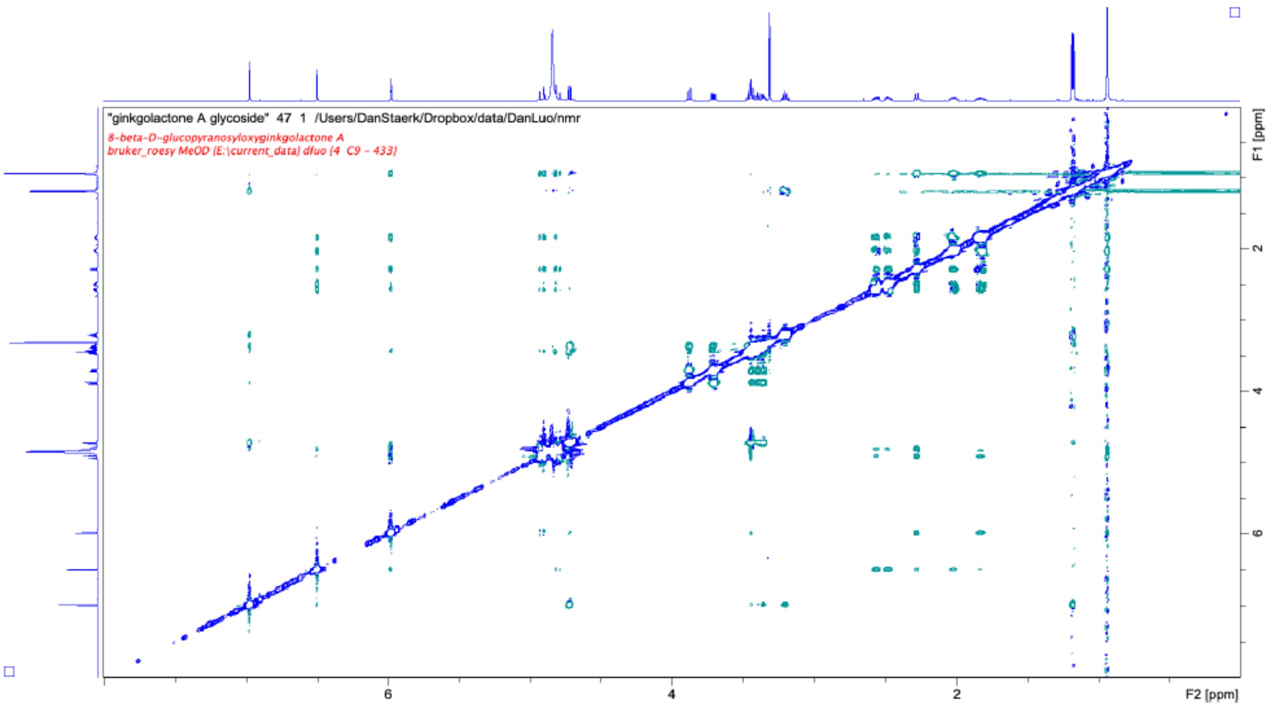

Supplementary Figure 48. ROESY spectrum of 8-b-D-glucopyranosyloxyginkgolactone A (**18c**) in methanol-*d*4.

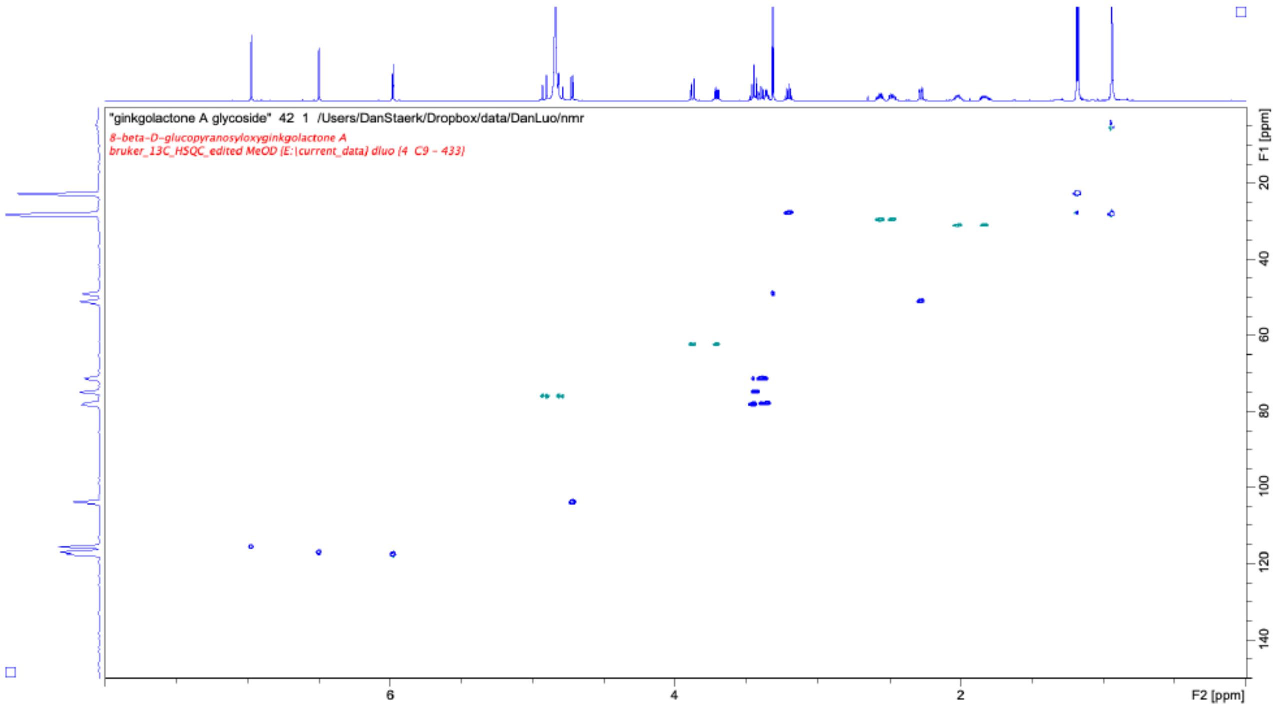

Supplementary Figure 49. Multiplicity-edited HSQC spectrum of 8-b-D-glucopyranosyloxyginkgolactone A (**18c**) in methanol-*d*4.

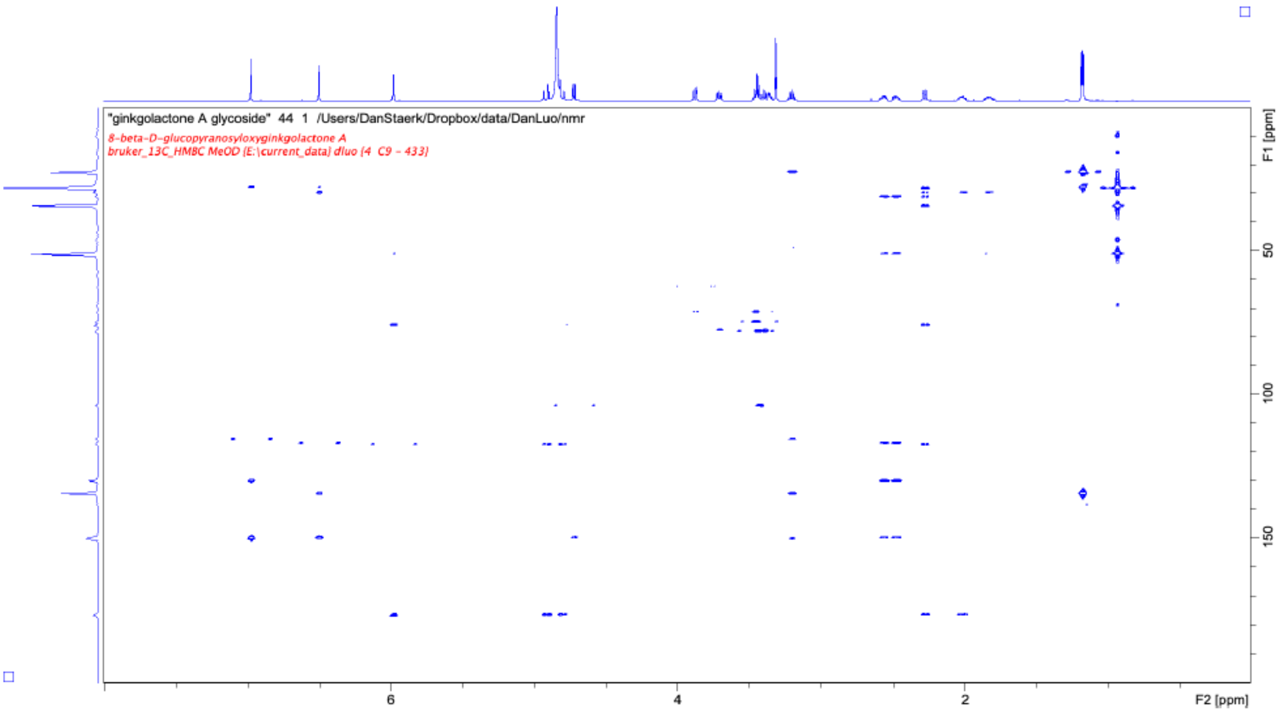

Supplementary Figure 50. HMBC spectrum of 8-b-D-glucopyranosyloxyginkgolactone A (**18c**) in methanol-d_4.

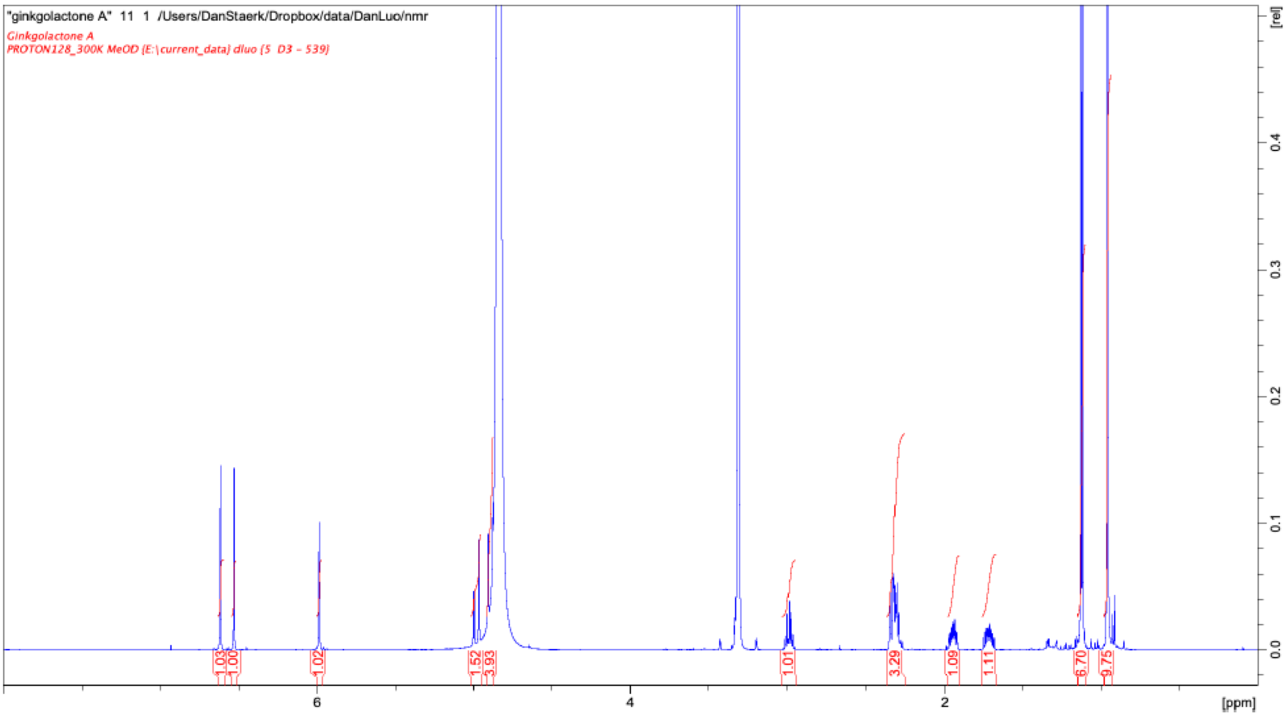

Supplementary Figure 51. ^{1}H NMR spectrum (600 MHz) of ginkgolactone A 1,4-benzoquinone (**23**) in methanol-d_4.

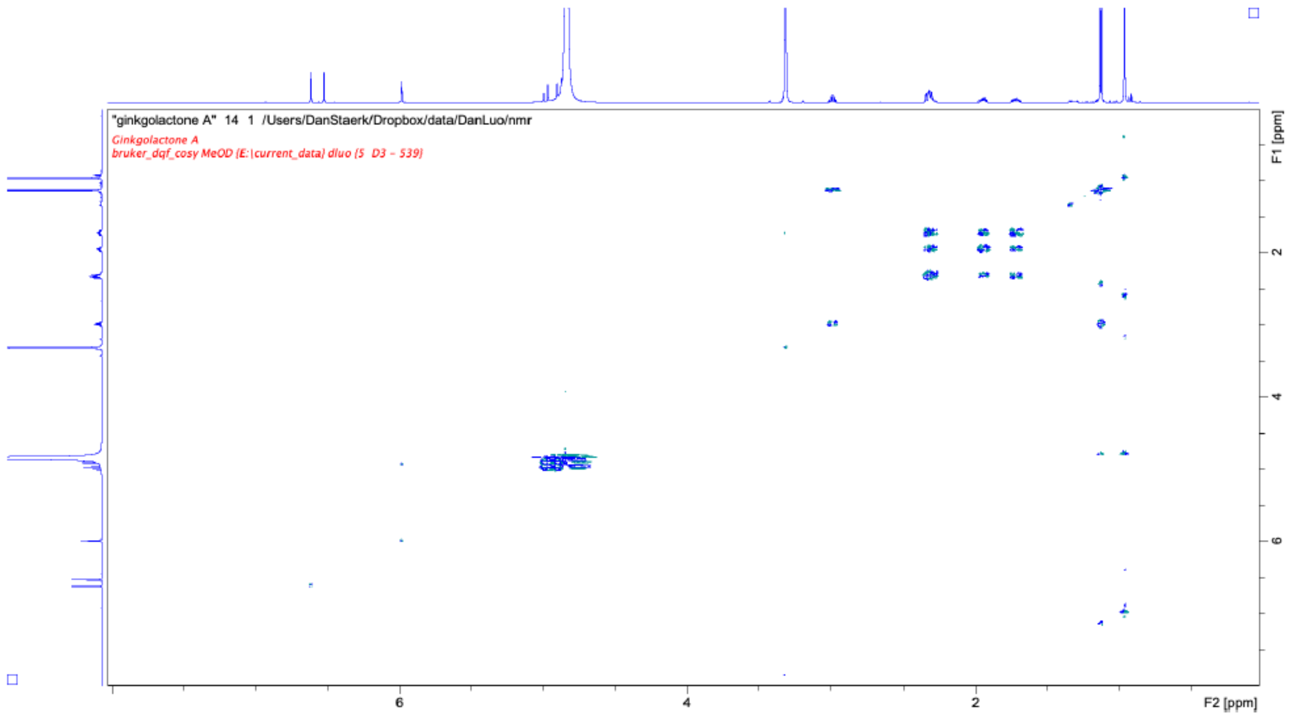

Supplementary Figure 52. COSY spectrum of ginkgolactone A 1,4-benzoquinone (**23**) in methanol-*d*4.

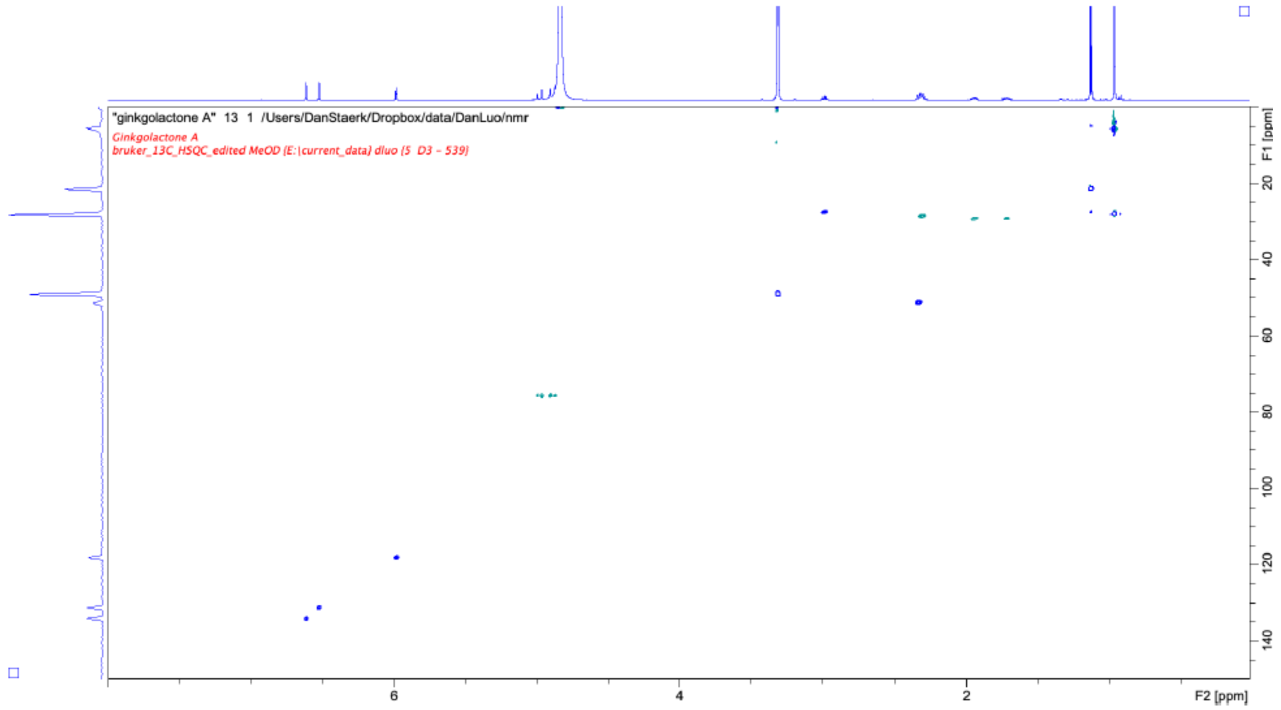

Supplementary Figure 53. Multiplicity-edited HSQC spectrum of ginkgolactone A 1,4-benzoquinone (**23**) in methanol-*d*4.

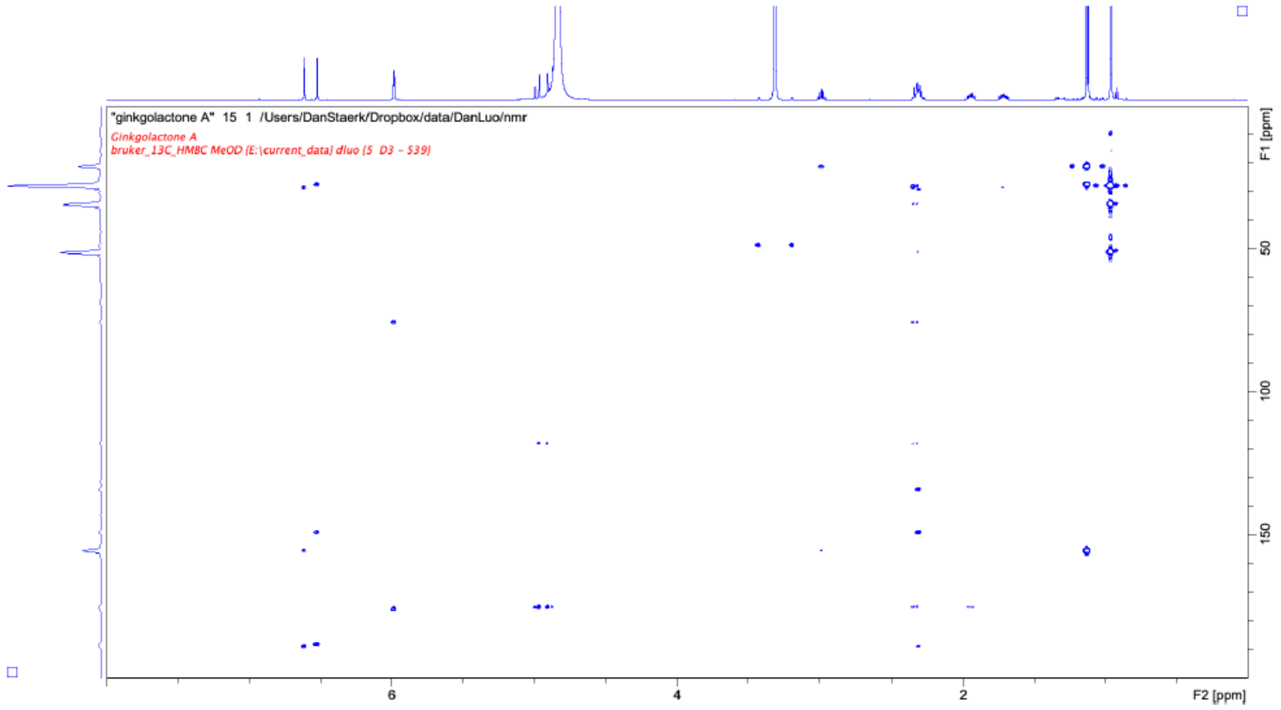

Supplementary Figure 54. HMBC spectrum of ginkgolactone A 1,4-benzoquinone (**23**) in methanol-*d*4.

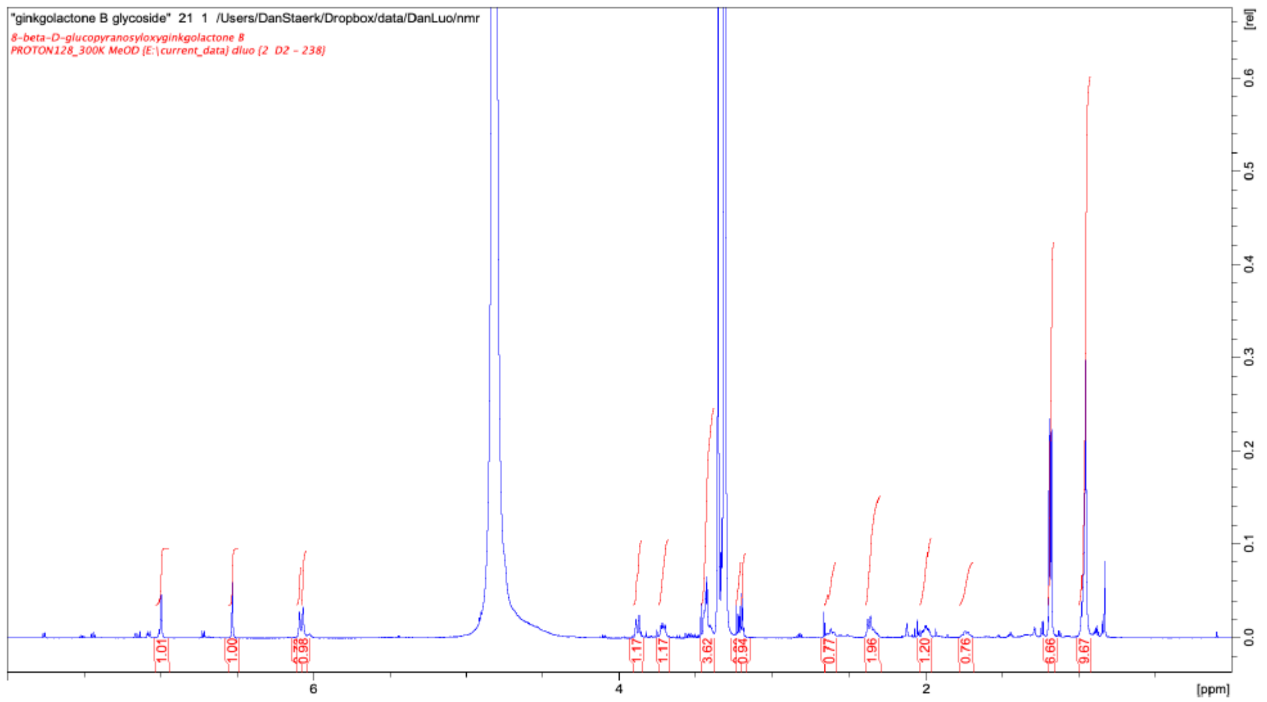

Supplementary Figure 55. 1H NMR spectrum (600 MHz) of 8-b-D-glucopyranosyloxyginkgolactone B (**19**) in methanol-*d*4.

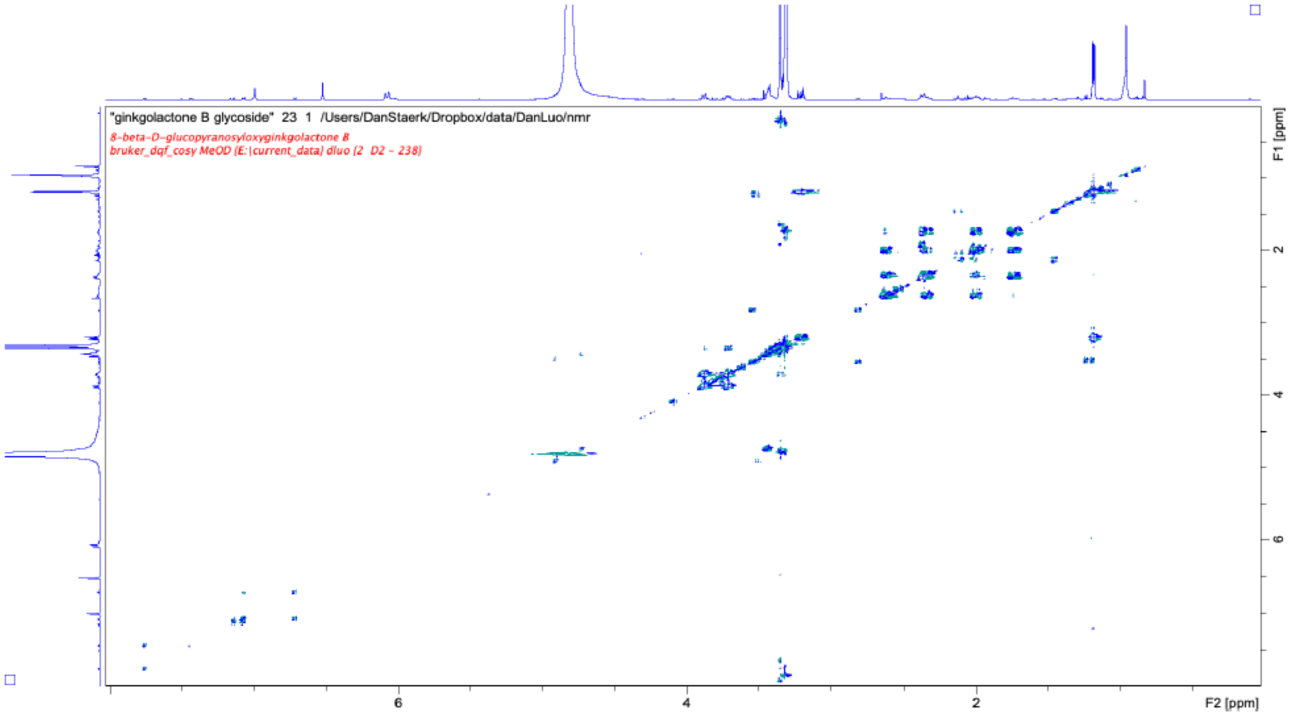

Supplementary Figure 56. COSY spectrum of 8-b-D-glucopyranosyloxyginkgolactone B (**19**) in methanol-*d*4.

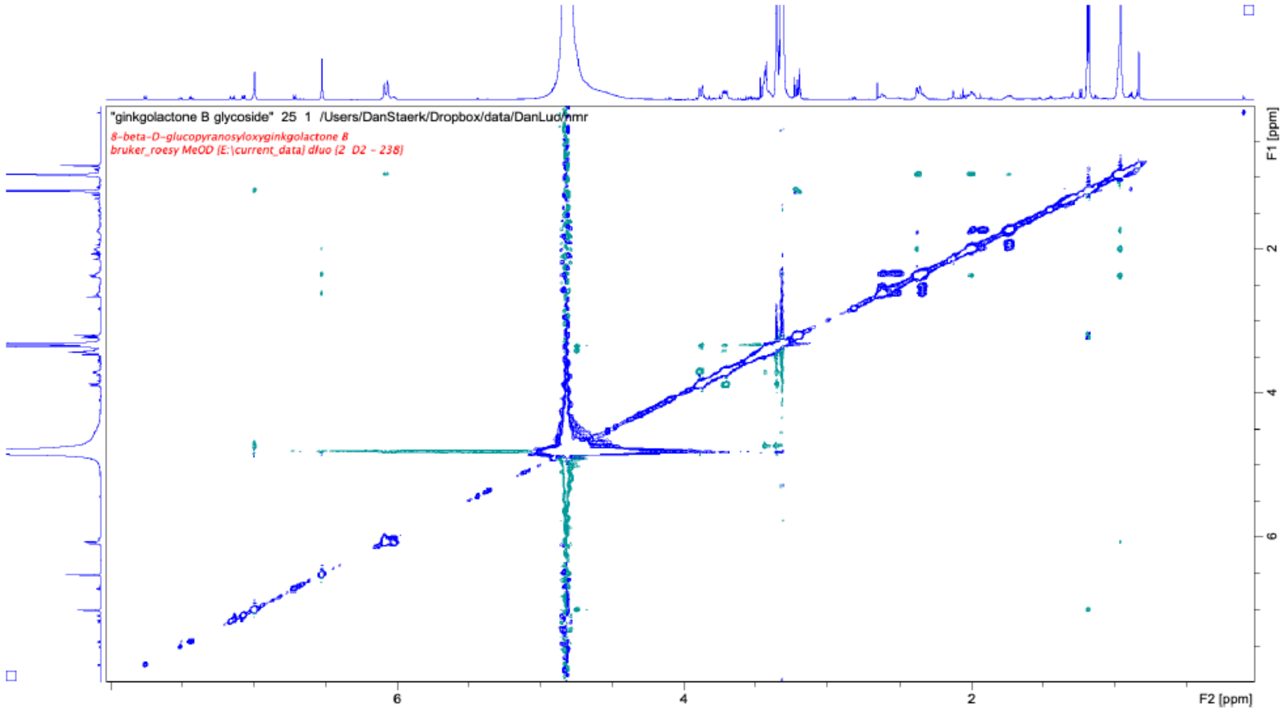

Supplementary Figure 57. ROESY spectrum of 8-b-D-glucopyranosyloxyginkgolactone B (**19**) in methanol-*d*4.

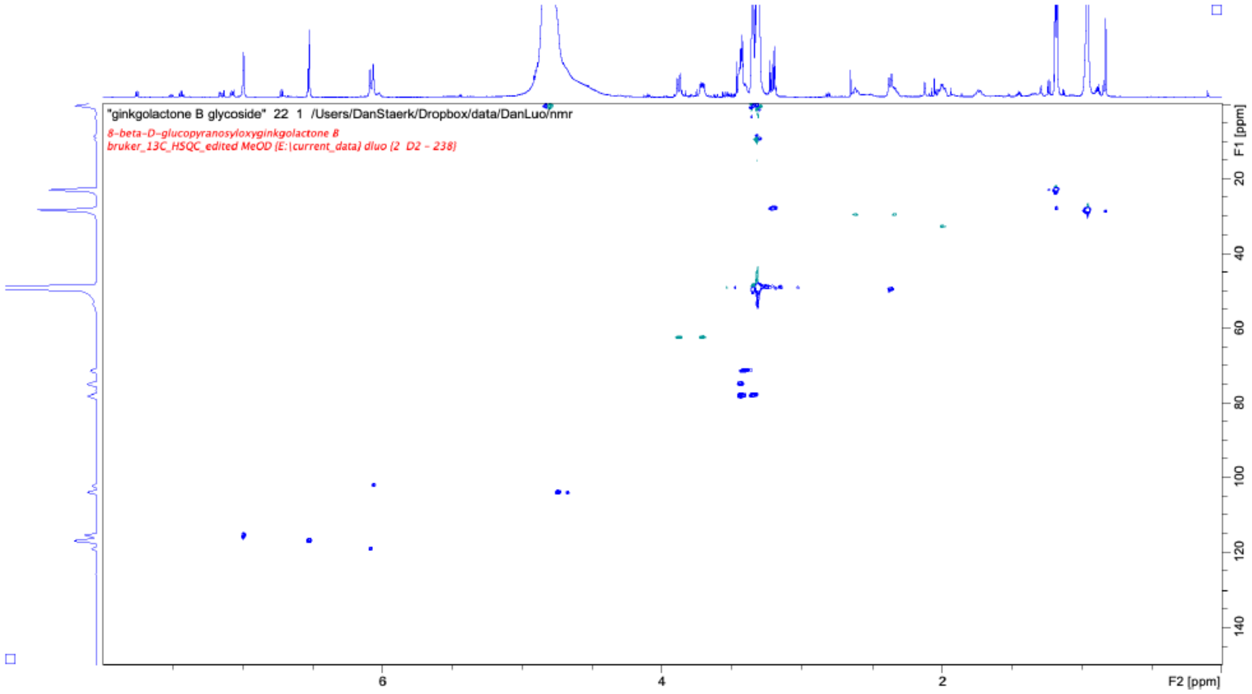

Supplementary Figure 58. Multiplicity-edited HSQC spectrum of 8-b-D-glucopyranosyloxyginkgolactone B (**19**) in methanol-d_4.

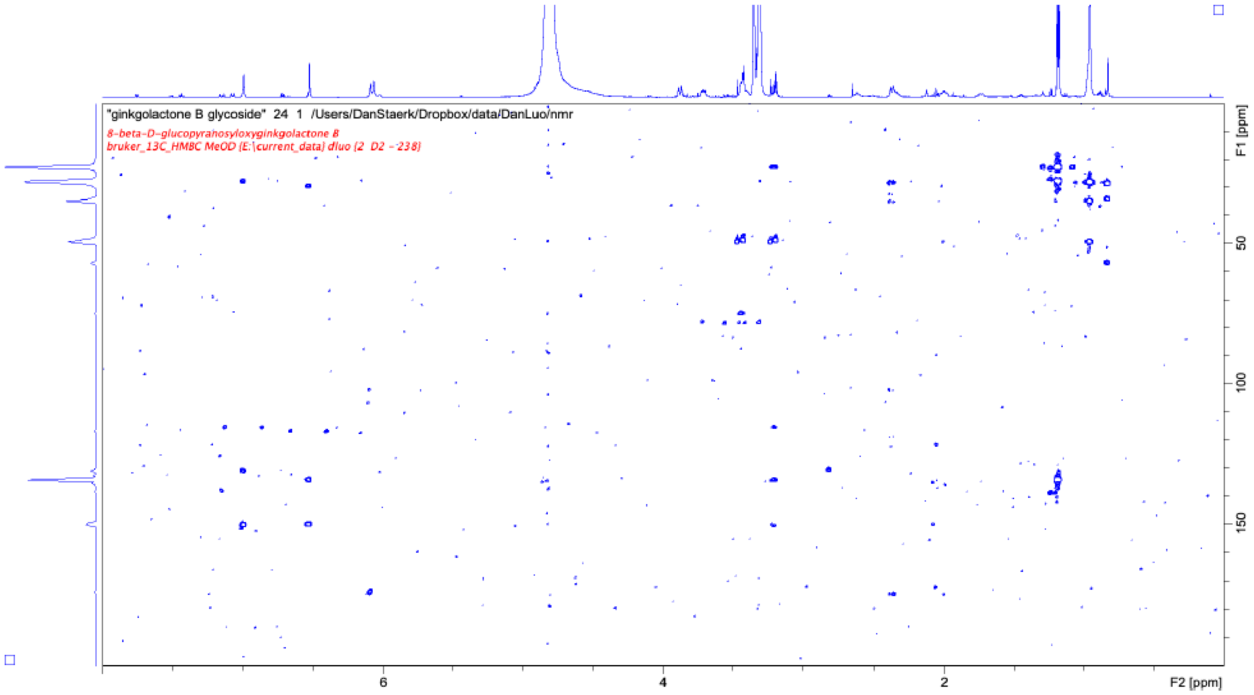

Supplementary Figure 59. HMBC spectrum of 8-b-D-glucopyranosyloxyginkgolactone B (**19**) in methanol-d_4.

Supplementary Table 1. Production titers of sandaracopimiradiene (**1**), paulastriene (**6**), levopimiradiene (**2**), dehydroabietadiene (**3**), abietadiene (**4**) and neoabietadiene (**5**) in yeast cells expressing *Gb*LPS variants. Source data are provided as a Source Data file.

Strain	**LPS version**	**1** (mg/L)	**6** (mg/L)	**2** (mg/L)	**3** (mg/L)	**4** (mg/L)	**5** (mg/L)	Total mg/L
sGIN1	None	0±0	0±0	0±0	0±0	0±0	0±0	0±0
sGIN2	FL	trace	trace	trace	trace	trace	trace	trace
sGIN3	Trunc.	3±0.8	2±0.1	11±2.8	1±0.1	38±8.8	5±1.5	59±13.6
sGIN4	Trunc./MBP	4±0.5	10±2.0	30±3.6	2±0.1	90±3.4	10±1.2	146±6.8

Supplementary Table 2. Genotypes of *Saccharomyces cerevisiae* strains used in this study.

Name	Plasmids used	Genotype
S288C	-	MATα, SUC2, gal2, mal2, mel ,flo1, flo8-1, ho, bio1, bio6,ura3Δ (NCYC3608).
sGIN1	pAS1_XI-2+pAS2+pAS3_XI-2	S288C, XI-2::[*Kl*URA3]
sGIN2	pGIN1+pAS2+pGIN2	S288C, XI-2::[pICL1-CO_*Sp*GGPPS7/pADH2-*Sc*tHMGR/pTDH3-FL_CO_*Gb*LPS/*Kl*URA3]
sGIN3	pGIN1+pAS2+pGIN3	S288C, XI-2::[pICL1-CO_*Sp*GGPPS7/pADH2-*Sc*tHMGR/pTDH3-t79_CO_*Gb*LPS/*Kl*URA3]
sGIN4	pGIN1+pAS2+pGIN5	S288C, XI-2::[pICL1-CO_*Sp*GGPPS7/pADH2-*Sc*tHMGR/pTDH3-t79_CO_*Gb*LPS_MBP/*Kl*URA3]
sGIN4	URA3 negative	S288C, XI-2::[pICL1-CO_*Sp*GGPPS7/pADH2-*Sc*tHMGR/pTDH3-t79_CO_*Gb*LPS_MBP], ura3Δ
sGIN5	pGIN6+pGIN8+pGIN9+pGIN11+pAS3_X-3	sGIN4A, X-3::[pICL1-CO_*Sp*GGPPS7/pTEF2-*Gb*POR1/pPGK1-t79_CO_*Gb*LPS_MBP/pTEF1-CO_*Gb*LPS_MBP /pTDH3-WT_*Gb*CYP7005C1/pENO2-CO_*Gb*CYP7005C1/*Kl*URA3]
sGIN6	pGIN6+pGIN8+pGIN10+pGIN12+pAS3_X-3	X-3::[pICL1-CO_*Sp*GGPPS7/pTEF2-*Gb*POR1/pPGK1-t79_CO_*Gb*LPS_MBP/pTEF1-CO_*Gb*LPS_MBP /pTDH3-WT_*Gb*CYP7005C3/pENO2-CO_*Gb*CYP7005C3/*Kl*URA3]
sGIN7	pGIN7+pGIN8+pGIN9+pGIN11+pAS3_X-3	X-3::[pICL1-CO_*Sp*GGPPS7/pTEF2-*Gb*POR2/pPGK1-t79_CO_*Gb*LPS_MBP/pTEF1-CO_*Gb*LPS_MBP /pTDH3-WT_*Gb*CYP7005C1/pENO2-CO_*Gb*CYP7005C1/*Kl*URA3]
sGIN8	pGIN7+pGIN8+pGIN10+pGIN12+pAS3_X-3	X-3::[pICL1-CO_*Sp*GGPPS7/pTEF2-*Gb*POR2/pPGK1-t79_CO_*Gb*LPS_MBP/pTEF1-CO_*Gb*LPS_MBP /pTDH3-WT_*Gb*CYP7005C3/pENO2-CO_CYP7005C3/*Kl*URA3]
sGIN9	pGIN6+pGIN17+pAS2B+pAS2C+pAS3_X-3	S288C, X-3::[pICL1-CO_*Sp*GGPPS7/pTEF2-*Gb*POR2/pPGK1-CO_t*Pb*TPS3/ pTEF1-CO_t*Pb*TPS1/*Kl*URA3]
sGIN10	pGIN6+pGIN17+pGIN9+pGIN12+pAS3_X-3	S288C, X-3::[pICL1-CO_*Sp*GGPPS7/pTEF2-*Gb*POR2/pPGK1-CO_t*Pb*TPS3/ pTEF1-CO_t*Pb*TPS1/pTDH3-WT_*Gb*CYP7005C1/pCCW12-CO_*Gb*CYP7005C3/*Kl*URA3]
sGIN11	pGIN7+pGIN8+pGIN10+pGIN11+pGIN13	X-3::[pICL1-CO_*Sp*GGPPS7/pTEF2-*Gb*POR2/pPGK1-t79_CO_*Gb*LPS_MBP/pTEF1-CO_*Gb*LPS_MBP /pTDH3-WT_*Gb*CYP7005C3/pENO2-CO_*Gb*CYP7005C1/pFBA1-WT_*Gb*CYP867E38/*Kl*URA3]
sGIN11	URA3 negative	X-3::[pICL1-CO_*Sp*GGPPS7/pTEF2-*Gb*POR2/pPGK1-t79_CO_*Gb*LPS_MBP/pTEF1-CO_*Gb*LPS_MBP /pTDH3-WT_*Gb*CYP7005C3/pENO2-CO_*Gb*CYP7005C1/pFBA1-WT_*Gb*CYP867E38], ura3Δ
sGIN12	pGIN14+pAS2+pAS3_XI-5	sGIN11, XI-5::[pTEF2- *Gb*POR2/*Kl*URA3]
sGIN13	pGIN14+pGIN15+pAS3_XI-5	sGIN11, XI-5::[pTEF2- *Gb*POR2/pCCW12-WT_*Gb*CYP867K1/pTDH3-CO_*Gb*CYP867K1/*Kl*URA3]
sGIN14	pGIN14+pGIN15+pGIN16	sGIN11, XI-5::[pTEF2- *Gb*POR2/pCCW12-WT_*Gb*CYP867K1/pTDH3-CO_*Gb*CYP867K1/pTDH3-WT_*Gb*CYP720B31/pCCW12-CO_*Gb*CYP720B31/*Kl*URA3]
sGIN15	pGIN14+pAS2A+pGIN9+pGIN11+pAS3_XI-5	S288C, XI-5::[pTEF2- *Gb*POR2/*Kl*URA3]
sGIN16	pGIN14+pAS2A+pGIN10+pGIN12+pAS3_XI-5	S288C, XI-5::[pTEF2- *Gb*POR2/pTDH3-WT_*Gb*CYP7005C1/pENO2-CO_*Gb*CYP7005C1/*Kl*URA3]
sGIN17	pGIN14+pAS2A+pGIN9+pGIN11+pAS3_XI-5	S288C, XI-5::[pTEF2- *Gb*POR2/pTDH3-WT_*Gb*CYP7005C3/pENO2-CO_*Gb*CYP7005C3/*Kl*URA3]

Supplementary Table 3. Structure and ^{1}H NMR (600 MHz) and ^{13}C NMR (150 MHz) data of ginkgosinoic acid A (**7**) in chloroform-*d*.

Structure of ginkgosinoic acid A (7) with carbons labeled

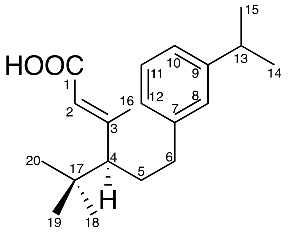

^{1}H NMR (600 MHz) and ^{13}C NMR (150 MHz) data of ginkgosinoic acid A (7) in chloroform-*d*

Pos.	δ_H (nH, multiplicity, *J* in Hz)	δ_C
1	-	173.1
2	5.75 (1H, br s)	119.0
3	-	162.1
4	1.91 (1H, dd, *J* = 11.8, 2.0 Hz)	59.9
5	A: 1.79 (1H, m)	29.7
	B: 1.86 (1H, m)	
6	A: 2.32 (1H, m)	34.6
	B: 2.54 (1H, br t, *J* ≈11.0 Hz)	
7	-	142.2
8	7.00 (1H, s)	126.5
9	-	148.9
10	7.04 (1H, d, *J* = 7.6 Hz)	123.8
11	7.19 (1H, t, *J* = 7.6 Hz)	128.3
12	6.97, 1H, (d, *J* = 7.6 Hz)	125.8
13	2.87 (1H, sept, *J* = 6.9 Hz)	34.0
14	1.24 (3H, d, *J* = 6.9 Hz)	24.1
15	1.24 (3H, d, *J* = 6.9 Hz)	24.1
16	2.17 (3H, s)	19.5
17	-	33.8
18	0.89 (3H, s)	28.6
19	0.89 (3H, s)	28.6
20	0.89 (3H, s)	28.6

Supplementary Table 4. Structure and ^{1}H NMR (600 MHz) and ^{13}C NMR (150 MHz) data of 2□-hydroxydehydroabietadiene (**8**) in chloroform-*d*.

Structure of 2α-hydroxydehydroabietadiene (8) with carbons labeled

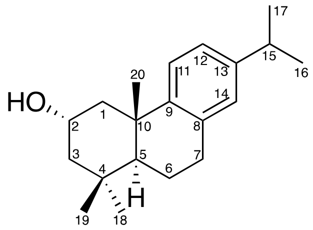

^{1}H NMR (600 MHz) and ^{13}C NMR (150 MHz) data

Pos.	δ_H (nH, multiplicity, *J* in Hz)	δ_C
1	ax: 1.35 (1H, m)	48.0
	eq: 2.65 (1H, ddd, *J* = 11.9, 4.1, 2.3 Hz)	
2	4.06 (1H, tt, *J* = 11.5, 4.1 Hz)	65.5
3	ax: 1.19 (1H, br dd, *J* = 12.3, 11.5 Hz)	50.8
	eq: 1.88 (1H, ddd, *J* = 12.3, 4.1, 2.3 Hz)	
4	-	34.9
5	1.36 (1H, dd, *J* = 12.6, 2.5 Hz)	49.7
6	ax: 1.72 (1H, dddd, *J* = 13.1, 12.6, 11.3, 7.1 Hz)	18.9
	eq: 1.89 (1H, m)	
7	ax: 2.86 (dd, *J* = 17.4, 11.3, 7.7 Hz)	30.5
	eq: 2.95 (1H, br dd, *J* = 17.4, 7.1 Hz)	
8	-	146.6
9	-	146.5
10	-	39.2
11	7.18 (1H, d, *J* = 8.2 Hz)	123.7
12	7.00 (1H, dd, *J* = 8.0, 1.5 Hz)	123.6
13	-	145.8
14	6.90 (1H, d, *J* = 1.5 Hz)	126.6
15	2.83 (1H, sept, *J* = 6.9 Hz)	33.4
16	1.22 (3H, d, *J* = 6.9 Hz)	23.8
17	1.22 (3H, d, *J* = 6.9 Hz)	23.8
18	1.01 (3H, s)	33.3
19	0.98 (3H, s)	22.3
20	1.21 (3H, br s)	25.5

Supplementary Table 5. Structure and ^{1}H NMR (600 MHz) and ^{13}C NMR (150 MHz) data of 2□-hydroxylevopimaradiene (**9**) in chloroform-*d*.

Structure of 2α-hydroxylevopimaradiene (9) with carbons labeled

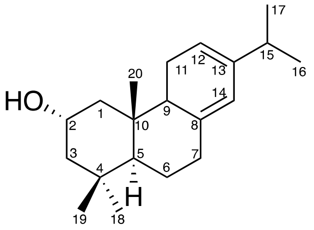

^{1}H NMR (600 MHz) and ^{13}C NMR (150 MHz) data

Pos.	δ_H (nH, multiplicity, *J* in Hz)	δ_C
1	ax: 0.84 (1H, m)	47.7
	eq: 2.06 (1H, m)	
2	3.85 (1H, tt, *J* = 11.5, 4.1 Hz)	65.4
3	ax: 1.12 (1H, br dd, *J* ≈ 12.3, 11.5 Hz)	51.7
	eq: 1.76 (1H, ddd, *J* = 12.3, 4.1, 2.5 Hz)	
4	-	35.1
5	1.04, 1H, (dd, *J* = 12.8, 2.7 Hz)	55.0
6	ax: 1.36 (1H, qd, *J* = 12.8, 4.4 Hz)	23.8
	eq: 1.72 (1H, dm, *J* = 12.8 Hz)	
7	ax: 2.08 (1H, m)	36.4
	eq: 2.35 (1H, m)	
8	-	138.5
9	2.09 (1H, m)	50.0
10	-	41.9
11	2.35 (2H, m)	23.4
12	5.17 (1H, t, *J* = 4.1 Hz)	115.0
13	-	138.9
14	5.56 (1H, br d, *J* = 1.5 Hz)	119.8
15	2.15 (1H, sept, *J* = 6.8 Hz)	33.7
16	0.98 (3H, d, *J* = 6.8 Hz)	21.4
17	0.98 (3H, d, *J* = 6.8 Hz)	21.4
18	0.94 (3H, s)	33.5
19	0.87 (3H, s)	22.7
20	0.90 (3H, s)	15.6

Supplementary Table 6. Structure and ^{1}H NMR (600 MHz) and ^{13}C NMR (150 MHz) data of ginkgosinoic acid B (**11**) in chloroform-*d*.

Structure of ginkgosinoic acid B (11) with carbons labeled

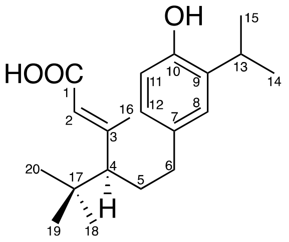

^{1}H NMR (600 MHz) and ^{13}C NMR (150 MHz) data

Pos.	δ_H (nH, multiplicity, J in Hz)	δ_C
1	-	172.0
2	5.71 (1H, br s)	119.0
3	-	163.3
4	1.89 (1H, dd, J = 11.8, 2.5 Hz)	60.0
5	A: 1.76 (1H, m)	30.2
	B: 1.83 (1H, m)	
6	A: 2.28 (1H, dt, J = 13.8, 8.4 Hz)	34.1
	B: 2.48 (1H, ddd, J = 13.8, 9.8, 4.5 Hz)	
7	-	134.4
8	6.95 (1H, d, J = 1.9 Hz)	126.2
9	-	134.2
10	-	151.0
11	6.65 (1H, d, J = 8.1 Hz)	115.5
12	6.82 (1H, dd, J = 8.1, 1.9 Hz)	126.7
13	3.18 (1H, sept, J = 6.9 Hz)	27.3
14	1.23 (3H, d, J = 6.9 Hz)[a]	22.9
15	1.24 (3H, d, J = 6.9 Hz)[a]	22.9
16	2.15 (3H, s)	19.8
17	-	34.0
18	0.88 (3H, s)	28.9
19	0.88 (3H, s)	28.9
20	0.88 (3H, s)	28.9

[a] May be interchanged

Supplementary Table 7. Structure and ^{1}H NMR (600 MHz) and ^{13}C NMR (150 MHz) data of 8-□-D-glucopyranosyloxyginkgosinoic acid C (**13d**) in methanol-d_4.

Structure of 8-β-D-glucopyranosyloxyginkgosinoic acid C (13d) with carbons labeled

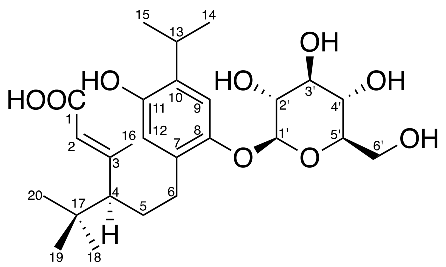

^{1}H NMR (600 MHz) and ^{13}C NMR (150 MHz) data

Pos.	δ_H (nH, multiplicity, J in Hz)	δ_C
1	-	170.4
2	5.74 (1H, br q, J = 1.1 Hz)	120.1
3	-	162.5
4	2.00 (1H, dd, J = 11.6, 3.0 Hz)	61.1
5	A: 1.77 (1H, m)	30.2
	B: 1.83 (1H, m)	
6	A: 2.32 (1H, m)	29.7
	B: 2.55 (1H, ddd, J = 13.1, 10.5, 5.0 Hz)	
7	-	131.2
8	-	150.0
9	6.99 (1H, s)	115.2
10	-	134.1
11	-	150.3
12	6.51 (1H, s)	117.1
13	3.21 (1H, sept, J = 6.9 Hz)	27.8
14	1.19 (3H, d, J = 6.9 Hz)^a	22.9
15	1.18 (3H, d, J = 6.9 Hz)^a	22.9
16	2.17 (3H, d, J = 1.1 Hz)	21.4
17	-	35.0
18	0.92 (3H, s)	28.9
19	0.92 (3H, s)	28.9
20	0.92 (3H, s)	28.9
1'	4.73 (1H, d, J = 7.5 Hz)	103.9
2'	3.44 (1H, m)	78.3
3'	3.39 (1H, m)	74.9
4'	3.44 (1H, m)	71.4
5'	3.35 (1H, m)	77.9
6'	A: 3.70 (1H, dd, J = 12.0, 5.5 Hz)	62.6
	B: 3.88 (1H, dd, J = 12.0, 2.2 Hz)	

^{a} May be interchanged

Supplementary Table 8. Structure and ^{1}H NMR (600 MHz) and ^{13}C NMR (150 MHz) data of ginkgosinoic acid C 1,4-benzoquinone (**17**) in methanol-d_4.

Structure of ginkgosinoic acid C 1,4-benzoquinone (17) with carbons labeled

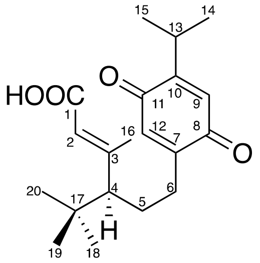

^{1}H NMR (600 MHz) and ^{13}C NMR (150 MHz) data

Pos.	δ_H (nH, multiplicity, *J* in Hz)	δ_C
1	-	171.1
2	5.69 (1H, br s)	121.9
3	-	158.9
4	1.94 (1H, dd, *J* = 11.6, 3.1 Hz)	61.0
5	1.75 (2H, m)	27.8
**6**	2.33 (1H, br ddd, *J* = 14.5, 9.8, 5.7 Hz)	28.8
7	-	149.9
8	-	189.2
9	6.52 (1H, d, *J* = 1.1 Hz)	131.3
10	-	155.6
11	-	188.5
12	6.58 (1H, t, *J* = 1.2 Hz)	133.9
13	2.99 (1H, sept d, *J* = 6.9, 1.1 Hz)	27.5
14	1.13 (3H, d, *J* = 6.9 Hz)	21.4
15	1.13 (3H, d, *J* = 6.9 Hz)	21.4
16	2.11 (3H, s)	n.d.
17	-	34.3
18	0.93 (3H, s)	28.8
19	0.93 (3H, s)	28.8
20	0.93 (3H, s)	28.8

n.d. Not detected

Supplementary Table 9. Structure and ^{1}H NMR (600 MHz) and ^{13}C NMR (150 MHz) data of 8-□-D-glucopyranosyloxyginkgolactone A (**18c**) in methanol-d_4.

Structure of 8-β-D-glucopyranosyloxyginkgolactone A (18c) with carbons labeled

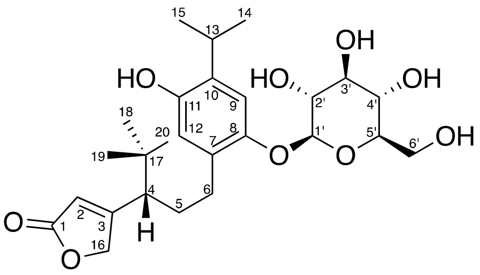

^{1}H NMR (600 MHz) and ^{13}C NMR (150 MHz) data

Pos.	δ_H (nH, multiplicity, *J* in Hz)	δ_C
1	-	177.0
2	5.98 (1H, br t, *J* = 1.7,)	117.8
3	-	176.5
4	2.28 (1H, dd, *J* = 11.7, 2.2 Hz)	51.4
5	A: 1.83 (1H, dddd, *J* = 13.5, 11.7, 8.9, 5.9 Hz)	31.5
	B: 2.02 (1H, dddd, *J* = 13.5, 9.4, 6.5, 2.2 Hz)	
6	A: 2.49 (1H, ddd, *J* = 13.5, 8.9, 6.5 Hz)	30.0
	B: 2.57 (1H, ddd, *J* = 13.5, 9.4, 5.9 Hz)	
7	-	130.5
8	-	150.3
9	6.97 (1H, s)	115.9
10	-	134.9
11	-	150.6
12	6.50 (1H, s)	117.3
13	3.20 (1H, sept, *J* = 6.9 Hz)	28.1
14	1.18 (3H, d, *J* = 6.9 Hz)	23.0
15	1.18 (3H, d, *J* = 6.9 Hz)	23.0
16	A: 4.80 (1H, dd, *J* = 18.1, 1.7 Hz)	76.3
	B: 4.91 (1H, dd, *J* = 18.1, 1.7 Hz)	
17	-	34.7
18	0.94 (3H, s)	28.4
19	0.94 (3H, s)	28.4
20	0.94 (3H, s)	28.4
1'	4.72 (1H, d, *J* = 7.1 Hz)	104.2
2'	3.44 (1H, m)	78.4
3'	3.39 (1H, m)	75.1
4'	3.44 (1H, m)	71.6
5'	3.35 (1H, ddd, *J* = 9.7, 5.6, 2.4 Hz)	78.1
6'	A: 3.71 (1H, dd, *J* = 12.0, 5.6 Hz)	62.7
	B: 3.87 (1H, dd, *J* = 12.0, 2.4 Hz)	

Supplementary Table 10. Structure and ^{1}H NMR (600 MHz) and ^{13}C NMR (150 MHz) data of 8-□-D-glucopyranosyloxyginkgolactone B (**19**) in methanol-d_4.

Structure of 8-β-D-glucopyranosyloxyginkgolactone B (19) with carbons labeled

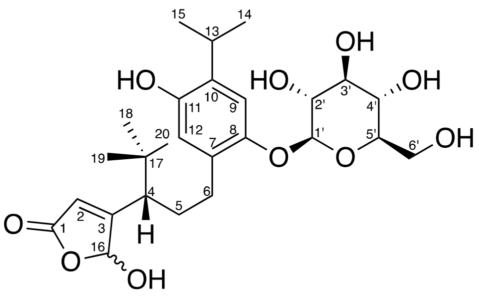

^{1}H NMR (600 MHz) and ^{13}C NMR (150 MHz) data

Pos.	δ_H (nH, multiplicity, J in Hz)	δ_C
1	-	173.9
2	6.09 (1H, s)	119.1
3	-	174.6
4	2.37 (1H, br d, J = 11.4 Hz)	49.8
5	A: 1.73 (1H, m)	32.7
	B: 2.00 (1H, m)	
6	A: 2.34 (1H, m)	29.8
	B: 2.62 (1H, m)	
7	-	131.0
8	-	150.1
9	6.99 (1H, s)	115.6
10	-	134.4
11	-	150.3
12	6.53 (1H, s)	117.0
13	3.20 (1H, sept, J = 6.9 Hz)	27.8
14	1.19 (3H, d, J = 6.9 Hz)[a]	22.9
15	1.18 (3H, d, J = 6.9 Hz)[a]	22.9
16	6.06 (1H, s)	102.2
17	-	35.2
18	0.96 (3H, s)	28.3
19	0.96 (3H, s)	28.3
20	0.96 (3H, s)	28.3
1'	4.74 (1H, d, J = 7.1 Hz)	104.0
2'	3.44 (1H, m)	78.2
3'	3.39 (1H, m)	74.9
4'	3.44 (1H, m)	71.3
5'	3.35 (1H, m)	78.0
6'	A: 3.71 (1H, dd, J = 11.7, 5.2 Hz)	62.5
	B: 3.86 (1H, dd, J = 11.7, 2.4 Hz)	

[a] May be interchanged

Supplementary Table 11. Structure and ^{1}H NMR (600 MHz) and ^{13}C NMR (150 MHz) data of ginkgolactone A 1,4-benzoquinone (**23**) in methanol-d_4.

Structure of ginkgolactone A 1,4-benzoquinone (23) with carbons labeled

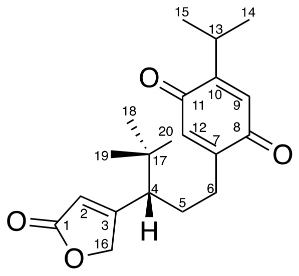

^{1}H NMR (600 MHz) and ^{13}C NMR (150 MHz) data

Pos.	δ_H (nH, multiplicity, J in Hz)	δ_C
1	-	175.9
2	5.98 (1H, t, J = 1.8 Hz)	118.0
3	-	175.1
4	2.33 (1H, dd, J = 11.7, 2.4 Hz)	51.3
5	A: 1.72 (1H, dddd, J = 13.4, 12.0, 9.2, 6.1 Hz)<br>B: 1.96 (1H, dddd, J = 13.4, 9.9, 6.5, 2.5 Hz)	29.3
6	2.31 (2H, m)	28.6
7	-	149.1
8	-	189.1
9	6.52 (1H, d, J = 1.2 Hz)	131.3
10	-	155.8
11	-	188.3
12	6.61 (1H, t, J = 1.2 Hz)	134.2
13	2.98 (1H, sept d, J = 6.9, 1.2 Hz)	27.5
14	1.13 (3H, d, J = 6.9 Hz)	21.3
15	1.13 (3H, d, J = 6.9 Hz)	21.3
16	A: 4.89 (1H, dd, J = 18.3, 1.8 Hz)	75.8
	B: 4.98 (1H, dd, J = 18.3, 1.8 Hz)	
17	-	34.3
18	0.96 (3H, s)	27.9
19	0.96 (3H, s)	27.9
20	0.96 (3H, s)	27.9

Supplementary Table 12. FPKM values of *Gb*LPS and co-expressed *Gb*CYPs in selected *G. biloba* transcriptomes.

CYP	Sterile seedling (FPKM)	Secondary stem 1 (FPKM)	Lateral roots 2 (FPKM)	Mature leaf 1 (FPKM)	Immature Fruit (FPKM)	Mature Fruit (FPKM)
***Gb*LPS**[a, c]	5	0	33	0	0	11
***Gb*CYP720B31**[b,c]	17	0	78	0	0.5	34
***Gb*CYP720B49**[b,c]	17	0	78	0	0.5	34
***Gb*CYP720B33**[a,c]	20	0,4	82	0	0.4	63
***Gb*CYP720B39**[a,d]	10	0	30	0	0	11
***Gb*CYP725A8**[b,c]	55	13	132	23	30	44
***Gb*CYP725A49**[b,c]	55	13	132	23	30	44
***Gb*CYP725A48**[b,c]	55	13	132	23	30	44
***Gb*CYP867E38**[a,c]	11	0	71	0	0.4	52
***Gb*CYP867K1**[a,c]	22	0	188	0	0.6	43
***Gb*CYP7005C1**[b,d]	60	0	398	0	2	222
***Gb*CYP7005C2**[b,d]	60	0	398	0	2	222
***Gb*CYP7005C3**[b,d]	60	0	398	0	2	222
***Gb*CYP728Q19**[a,c]	67	0	229	6	1	23
***Gb*CYP798B1**[a,c]	30	0	174	0	0.5	61
***Gb*CYP736Q2**[a,c]	19	0	69	0	0.7	36

[a] one gene per identifier, [b]multiple genes originating from one identifier, [c]full length transcript(s), [d]fragmented transcript(s).

Supplementary Table 13. Primers used to clone pJET1.2 constructs**.**

Name	Sequence	Type	Gene target	
CLN1	ATGGCTGGGGTGCTCTTTGCAAATCTGC	pJET1.2 cloning	*Gb*LPS	Fw
CLN2	CTACGCCACAGGATCGAAAAGAG	pJET1.2 cloning	*Gb*LPS	Rv
CLN3	ATGGCGAATAATACAGTAGTGTTGTTAG	pJET1.2 cloning	*Gb*CYP720B31	Fw
CLN4	TTATTCTCTCTTATACAATCGAATAGG	pJET1.2 cloning	*Gb*CYP720B31	Rv
CLN5	ATGGCGGAAAATGCATTACTGTTGTTG	pJET1.2 cloning	*Gb*CYP720B33	Fw
CLN6	TCATTCTCTACAATGTAATTGGATCG	pJET1.2 cloning	*Gb*CYP720B33	Rv
CLN7	ATGGCGGAAAATACATTGATTTCGTTG	pJET1.2 cloning	*Gb*CYP720B39	Fw
CLN8	TCATTCTTTATGATGCAATTGAATAGG	pJET1.2 cloning	*Gb*CYP720B39	Rv
CLN9	ATGGCGAATAATACAGTAGTGTTGG	pJET1.2 cloning	*Gb*CYP720B49	Fw
CLN10	TCATTCCCTATGATACAATTGAATAGG	pJET1.2 cloning	*Gb*CYP720B49	Rv
CLN11	ATGGAGTACTTGGATTTAATTCCTGC	pJET1.2 cloning	*Gb*CYP7005C1/C2/C3	Fw
CLN12	TTATATGTTTTTCTCCAAAACTCTAAAAG	pJET1.2 cloning	*Gb*CYP7005C1	Rv
CLN13	TTATATTTTCTTCTCCACAACTCTAAAG	pJET1.2 cloning	*Gb*CYP7005C2	Fw
CLN14	TTAGACGTTGAGTTCATGTAATTTCTTC	pJET1.2 cloning	*Gb*CYP7005C3	Rv
CLN15	atgttagccattattgttggaacg	pJET1.2 cloning	*Gb*CYP867K1	Fw
CLN16	ttagtagagatgtgcaggaagacg	pJET1.2 cloning	*Gb*CYP867K1	Rv
CLN17	ATGGAAATTAATTATTCTAGAGTTGC	pJET1.2 cloning	*Gb*CYP867E38	Fw
CLN18	CTAAGAGGAATTATAAAGATCAAGTG	pJET1.2 cloning	*Gb*CYP867E38	Rv
CLN19	atggacctatcgatcgcgacagttttag	pJET1.2 cloning	*Gb*CYP798B1	Fw
CLN20	ctataaatgtcgtggctcaattaag	pJET1.2 cloning	*Gb*CYP798B1	Rv
CLN21	ATGGCGTTTTATGGTATGTATGAG	pJET1.2 cloning	*Gb*CYP728Q19	Fw
CLN22	TCATTGTATCTTGACTTTAAGTTTG	pJET1.2 cloning	*Gb*CYP728Q19	Rv
CLN23	ATGGGTATTTTGTTGTGGATTAAGG	pJET1.2 cloning	*Gb*CYP725A8	Fw
CLN24	TCAGGATCTGGGAAACAATTTGATGG	pJET1.2 cloning	*Gb*CYP725A8	Rv
CLN25	ATGGGTATTTTGTTATGGGTCAAGG	pJET1.2 cloning	*Gb*CYP725A48	Fw
CLN26	TCAGGATCTCGGAAACAATATGATGG	pJET1.2 cloning	*Gb*CYP725A48	Rv
CLN27	ATGGGTATTTTGTTGTGGGGGAAGG	pJET1.2 cloning	*Gb*CYP725A49	Fw
CLN28	TCAGGATCTGGGAAATAATTTGATGGG	pJET1.2 cloning	*Gb*CYP725A49	Rv
CLN29	atgggcttgcaagagttgattcaaag	pJET1.2 cloning	*Gb*CYP736Q2	Fw
CLN30	ctaaacgacggatcctaagatgcc	pJET1.2 cloning	*Gb*CYP736Q2	Rv

Supplementary Table 14. pLIFE33 cloned constructs for transient expression in *Nicotiana benthamiana.*

Name	Backbone	Gene	Source
pNIC1	Unknown	P19	-
pNIC2	pLIFE33 (pCAMBIA1300Su)	*Cf*DXS	[14]
pNIC3	pLIFE33 (pCAMBIA1300Su)	*Cf*GGPPS	[14]
pNIC4	pLIFE33 (pCAMBIA1300Su)	*Gb*LPS	This study
pNIC5	pLIFE33 (pCAMBIA1300Su)	*Gb*CYP7005C1	This study
pNIC6	pLIFE33 (pCAMBIA1300Su)	*Gb*CYP7005C2	This study
pNIC7	pLIFE33 (pCAMBIA1300Su)	*Gb*CYP7005C3	This study
pNIC8	pLIFE33 (pCAMBIA1300Su)	*Gb*CYP867K1	This study
pNIC9	pLIFE33 (pCAMBIA1300Su)	*Gb*CYP867E38	This study
pNIC10	pLIFE33 (pCAMBIA1300Su)	*Gb*CYP720B31	This study
pNIC11	pLIFE33 (pCAMBIA1300Su)	*Gb*CYP720B33	This study
pNIC12	pLIFE33 (pCAMBIA1300Su)	*Gb*CYP720B39	This study
pNIC13	pLIFE33 (pCAMBIA1300Su)	*Gb*CYP720B49	This study
pNIC14	pLIFE33 (pCAMBIA1300Su)	*Gb*CYP798B1	This study
pNIC15	pLIFE33 (pCAMBIA1300Su)	*Gb*CYP725A8	This study
pNIC16	pLIFE33 (pCAMBIA1300Su)	*Gb*CYP736Q2	This study
pNIC17	pLIFE33 (pCAMBIA1300Su)	*Gb*CYP728Q19	This study
pNIC18	pLIFE33 (pCAMBIA1300Su)	*Gb*CYP725A48	This study
pNIC19	pLIFE33 (pCAMBIA1300Su)	*Gb*CYP725A49	This study
pNIC20	pLIFE33 (pCAMBIA1300Su)	*Sc*tHMGR	This study
pNIC21	pLIFE33 (pCAMBIA1300Su)	*Sp*GGPPS7	This study
pNIC21	pLIFE33 (pCAMBIA1300Su)	t*Gb*LPS	This study

Supplementary Table 15. Primers used to clone constructs for *Nicotiana benthamiana* transient expression.

Name	Sequence	Type	Gene target	Direction
GBA1	GGCTTAAUATGGCTGGGGTGCTCTTTGCAAATCTGC	USER cloning	*Gb*LPS	Fw
GBA2	GGTTTAAUCTACGCCACAGGATCGAAAAGAG	USER cloning	*Gb*LPS	Rv
GBA3	GGCTTAAUATGGCGAATAATACAGTAGTGTTGTTAG	USER cloning	*Gb*CYP720B31	Fw
GBA4	GGTTTAAUTTATTCTCTCTTATACAATCGAATAGG	USER cloning	*Gb*CYP720B31	Rv
GBA5	GGCTTAAUATGGCGGAAAATGCATTACTGTTGTTG	USER cloning	*Gb*CYP720B33	Fw
GBA6	GGTTTAAUTCATTCTCTACAATGTAATTGGATCG	USER cloning	*Gb*CYP720B33	Rv
GBA7	GGCTTAAUATGGCGGAAAATACATTGATTTCGTTG	USER cloning	*Gb*CYP720B39	Fw
GBA8	GGTTTAAUTCATTCTTTATGATGCAATTGAATAGG	USER cloning	*Gb*CYP720B39	Rv
GBA9	GGCTTAAUATGGCGAATAATACAGTAGTGTTGG	USER cloning	*Gb*CYP720B49	Fw
GBA10	GGTTTAAUTCATTCCCTATGATACAATTGAATAGG	USER cloning	*Gb*CYP720B49	Rv
GBA11	GGCTTAAUATGGAGTACTTGGATTTAATTCCTGC	USER cloning	*Gb*CYP7005C1/C2/C3	Fw
GBA12	GGTTTAAUTTATATGTTTTTCTCCAAAACTCTAAAAG	USER cloning	*Gb*CYP7005C1	Rv
GBA13	GGTTTAAUTTATATTTTCTTCTCCACAACTCTAAAG	USER cloning	*Gb*CYP7005C2	Rv
GBA14	GGTTTAAUTTAGACGTTGAGTTCATGTAATTTCTTC	USER cloning	*Gb*CYP7005C3	Rv
GBA15	GGCTTAAUatgttagccattattgttggaacg	USER cloning	*Gb*CYP867K1	Fw
GBA16	GGTTTAAUttagtagagatgtgcaggaagacg	USER cloning	*Gb*CYP867K1	Rv
GBA17	GGCTTAAUATGGAAATTAATTATTCTAGAGTTGC	USER cloning	*Gb*CYP867E38	Fw
GBA18	GGTTTAAUCTAAGAGGAATTATAAAGATCAAGTG	USER cloning	*Gb*CYP867E38	Rv
GBA19	GGCTTAAUatggacctatcgatcgcgacagttttag	USER cloning	*Gb*CYP798B1	Fw
GBA20	GGTTTAAUctataaatgtcgtggctcaattaag	USER cloning	*Gb*CYP798B1	Rv
GBA21	GGCTTAAUATGGCGTTTTATGGTATGTATGAG	USER cloning	*Gb*CYP728Q19	Fw
GBA22	GGTTTAAUTCATTGTATCTTGACTTTAAGTTTG	USER cloning	*Gb*CYP728Q19	Rv
GBA23	GGCTTAAUATGGGTATTTTGTTGTGGATTAAGG	USER cloning	*Gb*CYP725A8	Fw
GBA24	GGTTTAAUTCAGGATCTGGGAAACAATTTGATGG	USER cloning	*Gb*CYP725A8	Rv
GBA25	GGCTTAAUATGGGTATTTTGTTATGGGTCAAGG	USER cloning	*Gb*CYP725A48	Fw
GBA26	GGTTTAAUTCAGGATCTCGGAAACAATATGATGG	USER cloning	*Gb*CYP725A48	Rv
GBA27	GGCTTAAUATGGGTATTTTGTTGTGGGGGAAGG	USER cloning	*Gb*CYP725A49	Fw
GBA28	GGTTTAAUTCAGGATCTGGGAAATAATTTGATGGG	USER cloning	*Gb*CYP725A49	Rv
GBA29	GGCTTAAUatgggcttgcaagagttgattcaaag	USER cloning	*Gb*CYP736Q2	Fw
GBA30	GGTTTAAUctaaacgacggatcctaagatgcc	USER cloning	*Gb*CYP736Q2	Rv
GBA31	GGCTTAAUatggtcgcacaaactttcaacctgg	USER cloning	*Sp*GGPPS7	Fw
GBA32	GGTTTAAUttaatgctgacgacgtgtgatgaagtctgc	USER cloning	*Sp*GGPPS7	Rv
GBA33	GGCTTAAUATGGACCAATTGGTGAAAACTGAAGTCACCAAGAAG	USER cloning	*Sct*HMGR	Fw
GBA34	GGTTTAAUTTAGGATTTAATGCAGGTGACGGACCCATC	USER cloning	SctHMGR	Rv
GBA35	GGCTTAAUATGGCCTCAGCTGCAGAGACTCGTCCAG	USER cloning	t*Gb*LPS	Fw

Supplementary Table 16. Basic plasmids used and generated in this study for yeast.

Name	Type	Locus	NotI released cassette	Ref.
pX-2	Single integration	X-2	[DW-DR-URA3-DR-tCYC1-USER cassette – tADH1-UP]	[7]
pX-3	Single integration	X-3	[DW-DR-URA3-DR-tCYC1-USER cassette – tADH1-UP]	[7]
pX-4	Single integration	X-4	[DW-DR-URA3-DR-tCYC1-USER cassette – tADH1-UP]	[7]
pXI-2	Single integration	XI-2	[DW-DR-URA3-DR-tCYC1-USER cassette – tADH1-UP]	[7]
pXI-5	Single integration	XI-5	[DW-DR-URA3-DR-tCYC1-USER cassette – tADH1-UP]	[7]
pXII-2	Single integration	XII-2	[DW-DR-URA3-DR-tCYC1-USER cassette – tADH1-UP]	[7]
pXII-5	Single integration	XII-5	[DW-DR-URA3-DR-tCYC1-USER cassette – tADH1-UP]	[7]
pAS1_X-2	Assembler 1	X-2	[DW-DR-URA3-DR-tCYC1-USER cassette – tPGI1-tFBA1]	This study
pAS3_X-2	Assembler 3	X-2	[tENO2-tTDH2-USER cassette – tADH1-UP]	This study
pAS1_X-3	Assembler 1	X-3	[DW-DR-URA3-DR-tCYC1-USER cassette – tPGI1-tFBA1]	This study
pAS3_X-3	Assembler 3	X-3	[tENO2-tTDH2-USER cassette – tADH1-UP]	This study
pAS1_X-4	Assembler 1	X-4	[DW-DR-URA3-DR-tCYC1-USER cassette – tPGI1-tFBA1]	This study
pAS3_X-5	Assembler 3	X-4	[tENO2-tTDH2-USER cassette – tADH1-UP]	This study
pAS1_XI-2	Assembler 1	XI-2	[DW-DR-URA3-DR-tCYC1-USER cassette – tPGI1-tFBA1]	[8]
pAS3_XI-2	Assembler 3	XI-2	[tENO2-tTDH2-USER cassette – tADH1-UP]	[8]
pAS1_XI-5	Assembler 1	XI-5	[DW-DR-URA3-DR-tCYC1-USER cassette – tPGI1-tFBA1]	This study
pAS3_XI-5	Assembler 3	XI-5	[tENO2-tTDH2-USER cassette – tADH1-UP]	This study
pAS1_XII-2	Assembler 1	XII-2	[DW-DR-URA3-DR-tCYC1-USER cassette – tPGI1-tFBA1]	This study
pAS3_XII-2	Assembler 3	XII-2	[tENO2-tTDH2-USER cassette – tADH1-UP]	This study
pAS1_XII-5	Assembler 1	XII-5	[DW-DR-URA3-DR-tCYC1-USER cassette – tPGI1-tFBA1]	This study
pAS3_XII-5	Assembler 3	XII-5	[tENO2-tTDH2-USER cassette – tADH1-UP]	This study
pAS2	Assembler 2	-	[tPGI1-tFBA1-USER cassette - tENO2-tTDH2]	[8]
pAS2A	Assembler 2A	-	[tPGI1-tFBA1-USER cassette – tPRM9-tSPG5]	This study
pAS2B	Assembler 2B	-	[tPRM9-tSPG5-USER cassette – tCPS1-tPRM5]	This study
pAS2C	Assembler 2C	-	[tCPS1-tPRM5-USER cassette - tENO2-tTDH2]	This study

DR: Directed repeat
DW: Down
UP:Up

Supplementary Table 17. Cloned constructs for *Saccharomyces cerevisiae* genomic integration.

Name	Base plasmid	Promoter 1 (PGK)	Gene 1 (PGK)	Promoter 2 (TEF)	Gene 2 (TEF)
pGIN1	pAS1_XI-2	ICL1	CO_*Sp*GGPPS7	ADH2	*Sc*tHMGR
pGIN2	pAS3_XI-2			TDH3	FL_CO_*Gb*LPS
pGIN3	pAS3_XI-2			TDH3	t79_CO_*Gb*LPS
pGIN4	pAS3_XI-2			TDH3	FL_CO_*Gb*LPS_MBP
pGIN5	pAS3_XI-2			TDH3	t79_CO_*Gb*LPS_MBP
pGIN6	pAS1_X-3	ICL1	CO_*Sp*GGPPS7	TEF2	*Gb*POR1
pGIN7	pAS1_X-3	ICL1	CO_*Sp*GGPPS7	TEF2	*Gb*POR2
pGIN8	pAS2A	PGK1	CO_t79_*Gb*LPS	TEF1	CO_t79_*Gb*LPS_MBP
pGIN9	pAS2B	TDH3	WT_*Gb*CYP7005C1		
pGIN10	pAS2B	TDH3	WT_*Gb*CYP7005C3		
pGIN11	pAS2C			ENO2	CO_*Gb*CYP7005C1
pGIN12	pAS2C			ENO2	CO_*Gb*CYP7005C3
pGIN13	pAS3_X-3	FBA1	WT_*Gb*CYP867E38		
pGIN14	pAS1_XI-5			TEF2	*Gb*POR2
pGIN15	AS2	CCW12	WT_*Gb*CYP867K1	TDH3	CO_*Gb*CYP867K1
pGIN16	AS3_XI-5	TDH3	WT_*Gb*CYP720B31	CCW12	CO_*Gb*CYP720B31
pGIN17	pAS2A	PGK1	CO_t*Pb*TPS3	TEF1	CO_t*Pb*TPS1

FL: Full length
t: Truncated
CO: Codon optimized

Supplementary Table 18. Primers used to amplify DNA blocks for constructing the new Assembler plasmids.

Name	Sequence	Type	Target	Fw/Rv
VEC1	AGCGATTUGTCCGCGGCCGCAAATTTAAATAAAATG	USER cloning	Assembler 2 backbone	Fw
VEC2	AGTTAAAUCCGCGGCCGCATTTAAATCCCCACTT	USER cloning	Assembler 2 backbone	Rv
VEC3	ATTTAACUCCTTAAGTTACTTTAATGATTTAG	USER cloning	tTDH2_part_Ass2	Fw
VEC4	ATGCATGCAUGCTGCTCATGTGACAAGTATTGATGAT	USER cloning	tTDH2_part_Ass2	Rv
VEC5	ATGCATGCAUTTCCTTCCATCTTGTGATTCATGC	USER cloning	*Gb*lock_part	Fw
VEC6	ATGCATGCUCCAAAATGAGCTATCAAAAACGAT	USER cloning	*Gb*lock_part	Rv
VEC7	AGCATGCAUGCTTTGAAACTAAATTTGTATATT	USER cloning	tPGI1_part_Ass2	Fw
VEC8	AAATCGCUCTTAAATATATACCTAAAGAACAT	USER cloning	tPGI1_part_Ass2	Rv
VEC9	AACGGAAUGCGTGCGATCGCGTGCATTCATCC	USER cloning	Assembler 1 backbone	Fw
VEC10	ACCGCGGCCGCAUTTAAATCCCCACTTCAGAAGTTCC	USER cloning	Assembler 1 backbone	Rv
VEC11	ATGCGGCCGCGGUTAATTCAAATTAATTGATATAGTTTTTTAATGAG	USER cloning	tFBA1_part	Fw
VEC12	ATGCATGCUCCAAAATGAGCTATCAAAAACGAT	USER cloning	tFBA1_part	Rv
VEC13	AGCATGCAUGCTTTGAAACTAAATTTGTATATTGTTTGTC	USER cloning	tPGI1_part_Ass1	Fw
VEC14	ATTCCGTUGGGACAAATCGCTCTTAAATATATACC	USER cloning	tPGI1_part_Ass1	Rv
VEC15	AAGCACUCCGCGGCCGCAAATTTAAATAAAATG	USER cloning	Assembler 3 backbone	Fw
VEC16	ATCCGAAUGCACGCGATCGCACGCATTCCG	USER cloning	Assembler 3 backbone	Rv
VEC17	ATTCGGAUTTAACTCCTTAAGTTACTTTAATGATTTAG	USER cloning	tTDH2_part_Ass3	Fw
VEC18	ATGCATGCUCATGTGACAAGTATTGATGATAGT	USER cloning	tTDH2_part_Ass3	Rv
VEC19	AGCATGCAUGCATTTCCTTCCATCTTGTGATTC	USER cloning	tENO2_part	Fw
VEC20	AGTGCTUTTAACTAAGAATTATTAGTCTTTTCTGC	USER cloning	tENO2_part	Rv
VEC21	ATAGGCUCCGCCCCCCTGACGAGCATCA	USER cloning	To split Ass1/3 PCR	Fw
VEC22	AGCCTAUGGAAAAACGCCAGCAACGCGGCCTT	USER cloning	To split Ass1/3 PCR	Rv
VEC23	AGCGATTUGTCCGCGGCCGCAAATTTAAATAAAATG	USER cloning	Assembler2A_backbone	Fw
VEC24	ACGTCTTUGGCGGCCGCATTTAAATCCCCACTTC	USER cloning	Assembler2A_backbone	Rv
VEC25	AAAGACGUTGTTTCATCGCGCTATTACCAAGAAG	USER cloning	tSPG5_part_Ass2A	Fw
VEC26	AAATGCTUATTTTCTGCCGAATTTTCATGAAG	USER cloning	tSPG5_part_Ass2A	Rv
VEC27	AAGCATTUTCAACATCGTATTTTCCGAAGCGT	USER cloning	tPRM9_part_Ass2A	Fw
VEC28	ATTCCGTUGACAGAAGACGGGAGACACTAGCAC	USER cloning	tPRM9_part_Ass2A	Rv
VEC29	AACGGAAUGCGTGCGATCGCGTGCATTC	USER cloning	USER_part_Ass2A	Fw
VEC30	AAATCGCUCTTAAATATATACCTAAAGAACAT	USER cloning	USER_part_Ass2A	Rv
VEC31	AATCATUGCGCGCGGCCGCAAATTTAAATAAAATG	USER cloning	Assembler2C_backbone	Fw
VEC32	AGTTAAAUCCGCGGCCGCATTTAAATCCCCACTT	USER cloning	Assembler2C_backbone	Rv
VEC33	ATTTAACUCCTTAAGTTACTTTAATGATTTAGTTTT	USER cloning	USER_part_Ass2C	Fw
VEC34	AGTTTGAAUGCACGCGATCGCACGCATTCC	USER cloning	USER_part_Ass2C	Rv
VEC35	ATTCAAACUTTTATGATATTTTGCAATATTTTTTTTAAGCAGTGG	USER cloning	tPRM5_part_Ass2C	Fw
VEC36	AGTGTCAAAUATAGAACCCAAAAAGAGAGACTAA	USER cloning	tPRM5_part_Ass2C	Rv
VEC37	ATTTGACACUTGATTTGACACTTCTTTTTTTTTT	USER cloning	tCPS1_part_Ass2C	Fw
VEC38	AATGATUGAATAGTCAAAGATTTTTTTTTTT	USER cloning	tCPS1_part_Ass2C	Rv
VEC39	AATTTAAAUAAAATGAAGTGAAGTTCCTATACTTTCTAGAG	USER cloning	Assembler2B_backbone	Fw
VEC40	AAAGTTUGCGGCCGCATTTAAATCCCCACTT	USER cloning	Assembler2B_backbone	Rv
VEC41	AAACTTUTATGATATTTTGCAATATTTTTTTTAAGC	USER cloning	Ass2C_term_USER_Site	Fw
VEC42	ATCGCACGCAUTCCGTTGGCGCAATGATTGAATAGTCAAAGA	USER cloning	Ass2C_term_USER_Site	Rv
VEC43	ATGCGTGCGAUCGCGTGCATTCCAAAGACGTTGTTTCATCGCGCT	USER cloning	Ass2A_term_USER_Site	Fw
VEC44	ATTTAAATUTGCGGCCGCACAGAAGACGGGAGACACTAGCAC	USER cloning	Ass2A_term_USER_Site	Rv

Supplementary Table 19. Primers used to amplify genes for *Saccharomyces cerevisiae* constructs.

Name	Sequence	Type	Gene target	Fw/Rv
GBA101	AGCGATACGUAAAATGGTGACCAGCAAAGAGGATCTCACCTCC	USER TEF	*Gb*POR1_TEF	Fw
GBA102	CACGCGAUTCACCATACATCTCGCAAGTACCTTCC	USER TEF	*Gb*POR1_TEF	Rv
GBA103	AGCGATACGUAAAATGGGCAACATGTTGTCTTTCGTTTCC	USER TEF	*Gb*POR2_TEF	Fw
GBA104	CACGCGAUTCACCATACATCTCTCAAGTATCGACC	USER TEF	*Gb*POR2_TEF	Rv
GBA208	AGCGATACGUAAAATGGCTGGTGTTTTGTTTGCTAACTTGC	USER TEF	CO_*Gb*LPS_TEF	Fw
GBA209	CACGCGAUTTAGGCAACTGGATCAAACAAAGTTCTAG	USER TEF	CO_*Gb*LPS_TEF	Rv
GBA213	ACCGGCAACUGGATCAAACAAAGTTCTAGAAACG	USER fusion	CO_*Gb*LPS_fus_MBP	Rv
GBA214	AGTTGCCGGUGGTGGTGGCGGTGGTTCTAAAATT	USER fusion	CO_MBP_GA_TEF	Fw
GBA215	CACGCGAUTTACTTGGTGATTCTAGTTTGAGCATC	USER fusion	CO_MBP_GA_TEF	Rv
GBA227	ATCAACGGGUAAAATGTTGGCCATTATCGTTGGTACTACC	USER PGK	CO_CYP867K1_PGK	Fw
GBA228	CGTGCGAUTTAGTACAAGTGAGCTGGCAATCTAGG	USER PGK	CO_CYP867K1_PGK	Rv
GBA229	AGCGATACGUAAAATGTTGGCCATTATCGTTGGTACTACC	USER TEF	CO_CYP867K1_TEF	Fw
GBA230	CACGCGAUTTAGTACAAGTGAGCTGGCAATCTAGG	USER TEF	CO_CYP867K1_TEF	Rv
GBA247	AGCGATACGUAAAATGTTGAACGCTGATTATCATCCAGCTG	USER TEF	CO_t79*Gb*LPS_TEF	Fw
GBA265	ATCAACGGGUAAAATGGCTAACAACACCGTTGTTTTGTTGG	USER PGK	CO_CYP720B31_PGK	Fw
GBA266	CGTGCGAUTCATTCCCTCTTGTACAACCTAATTGG	USER PGK	CO_CYP720B31_PGK	Rv
GBA267	AGCGATACGUAAAATGGCTAACAACACCGTTGTTTTGTTGG	USER TEF	CO_CYP720B31_TEF	Fw
GBA268	CACGCGAUTCATTCCCTCTTGTACAACCTAATTGG	USER TEF	CO_CYP720B31_TEF	Rv
GBA275	ATCAACGGGUAAAATGGAAATTAATTATTCTAGAGTTGCTGTG	USER PGK	*Gb*CYP867E38_PGK	Fw
GBA276	CGTGCGAUCTAAGAGGAATTATAAAGATCAAGTGAGAG	USER PGK	*Gb*CYP867E38_PGK	Rv
GBA297	ATCAACGGGUAAAATGGAATACTTGGATTTGATTCCAGCTAC	USER PGK	CO_CYP7005C1_PGK	Fw
GBA298	CGTGCGAUTTAGATGTTCTTCTCCAAAACTCTAAAAGG	USER PGK	CO_CYP7005C1_PGK	Rv
GBA299	AGCGATACGUAAAATGGAATACTTGGATTTGATTCCAGCTAC	USER TEF	CO_CYP7005C1_TEF	Fw
GBA300	CACGCGAUTTAGATGTTCTTCTCCAAAACTCTAAAAGG	USER TEF	CO_CYP7005C1_TEF	Rv
GBA301	ATCAACGGGUAAAATGGAATATTTGGATTTAATTCCAGCAAC	USER PGK	CO_CYP7005C3_PGK	Fw
GBA302	CGTGCGAUTTAAACGTTCAATTCGTGCAACTTTTTTTC	USER PGK	CO_CYP7005C3_PGK	Rv
GBA303	AGCGATACGUAAAATGGAATATTTGGATTTAATTCCAGCAAC	USER TEF	CO_CYP7005C3_TEF	Fw
GBA304	CACGCGAUTTAAACGTTCAATTCGTGCAACTTTTTTTC	USER TEF	CO_CYP7005C3_TEF	Rv

Supplementary references

1. Ni, J. *et al.* Comprehensive transcriptome analysis and flavonoid profiling of Ginkgo leaves reveals flavonoid content alterations in day–night cycles. *PLOS One* **13**, e0193897 (2018).
2. Han, S.M., Wu, Z.J., Jin, Y., Yang, W.N. & Shi, H.Z. RNA-Seq analysis for transcriptome assembly, gene identification, and SSR mining in ginkgo (*Ginkgo biloba* L.). *Tree Genetics & Genomes* **11** (2015).
3. Guan, R. *et al.* Draft genome of the living fossil *Ginkgo biloba*. *GigaScience* **5**, 49 (2016).
4. Jones, D.T., Taylor, W.R. & Thornton, J.M. The rapid generation of mutation data matrices from protein sequences. *Comput Appl Biosci* **8**, 275-282 (1992).
5. Kumar, S., Stecher, G., Li, M., Knyaz, C. & Tamura, K. MEGA X: Molecular evolutionary genetics analysis across computing platforms. *Mol Biol Evol* **35**, 1547-1549 (2018).
6. Nour-Eldin, H.H., Hansen, B.G., Norholm, M.H.H., Jensen, J.K. & Halkier, B.A. Advancing uracil-excision based cloning towards an ideal technique for cloning PCR fragments. *Nucleic Acids Res* **34** (2006).
7. Mikkelsen, M.D. *et al.* Microbial production of indolylglucosinolate through engineering of a multi-gene pathway in a versatile yeast expression platform. *Metabolic Engineering* **14**, 104-111 (2012).
8. Vanegas, K.G., Lehka, B.J. & Mortensen, U.H. SWITCH: a dynamic CRISPR tool for genome engineering and metabolic pathway control for cell factory construction in *Saccharomyces cerevisiae*. *Microbial Cell Factories* **16**, 25 (2017).
9. Gulck, T. *et al.* Synthetic biology of cannabinoids and cannabinoid glucosides in *Nicotiana benthamiana* and *Saccharomyces cerevisiae*. *Journal of Natural Products* **83**, 2877-2893 (2020).
10. Jensen, N.B. *et al.* EasyClone: method for iterative chromosomal integration of multiple genes *Saccharomyces cerevisiae*. *FEMS Yeast Research* **14**, 238-248 (2014).
11. Geu-Flores, F., Nour-Eldin, H.H., Nielsen, M.T. & Halkier, B.A. USER fusion: a rapid and efficient method for simultaneous fusion and cloning of multiple PCR products. *Nucleic acids research* **35**, e55-e55 (2007).
12. Genee, H.J. *et al.* Software-supported USER cloning strategies for site-directed mutagenesis and DNA assembly. *ACS Synthetic Biology* **4**, 342-349 (2015).
13. Liu, T., Zhang, C. & Lu, W. Heterologous production of levopimaric acid in *Saccharomyces cerevisiae*. *Microbial Cell Factories* **17**, 114 (2018).
14. Andersen-Ranberg, J. *et al.* Expanding the landscape of diterpene structural diversity by stereochemically controlled combinatorial biosynthesis. *Angewandte Chemie-International Edition* **55**, 2142-2146 (2016).
