# Supplementary material for: A gene cluster in Ginkgo biloba encodes unique multifunctional cytochrome P450s that initiate ginkgolide biosynthesis
Source: Nat Commun. 2022 Sep 1;13:5143. doi: 10.1038/s41467-022-32879-9 (PMC9436924; doi:10.1038/s41467-022-32879-9)
Supplement: Supplementary file 3 — Description of Additional Supplementary Files [file 41467_2022_32879_MOESM3_ESM.pdf]

Description of Additional Supplementary Files

File Name: Supplementary Data 1
Description: GbCYPs transiently co-expressed with GbLPS in Nicotiana benthamiana

File Name: Supplementary Data 2
Description: Primers used to amplify promoter sequences and additional primers used for Saccharomyces cerevisiae

File Name: Supplementary Data 3
Description: Codon optimized Ginkgo biloba sequences for yeast expression
