# Supplementary material for: A gene cluster in Ginkgo biloba encodes unique multifunctional cytochrome P450s that initiate ginkgolide biosynthesis
Source: Nat Commun. 2022 Sep 1;13:5143. doi: 10.1038/s41467-022-32879-9 (PMC9436924; doi:10.1038/s41467-022-32879-9)
Supplement: Supplementary file 7 — Reporting Summary [file 41467_2022_32879_MOESM7_ESM.pdf]

nature portfolio

Corresponding author(s): Irini Pateraki, PhD

Last updated by author(s): Jul 23, 2022

Reporting Summary

Nature Portfolio wishes to improve the reproducibility of the work that we publish. This form provides structure for consistency and transparency in reporting. For further information on Nature Portfolio policies, see our Editorial Policies and the Editorial Policy Checklist.

Statistics

For all statistical analyses, confirm that the following items are present in the figure legend, table legend, main text, or Methods section.

n/a	Confirmed	
☐	☒	The exact sample size (*n*) for each experimental group/condition, given as a discrete number and unit of measurement
☐	☒	A statement on whether measurements were taken from distinct samples or whether the same sample was measured repeatedly
☒	☐	The statistical test(s) used AND whether they are one- or two-sided<br>*Only common tests should be described solely by name; describe more complex techniques in the Methods section.*
☒	☐	A description of all covariates tested
☒	☐	A description of any assumptions or corrections, such as tests of normality and adjustment for multiple comparisons
☐	☒	A full description of the statistical parameters including central tendency (e.g. means) or other basic estimates (e.g. regression coefficient) AND variation (e.g. standard deviation) or associated estimates of uncertainty (e.g. confidence intervals)
☒	☐	For null hypothesis testing, the test statistic (e.g. *F*, *t*, *r*) with confidence intervals, effect sizes, degrees of freedom and *P* value noted<br>*Give P values as exact values whenever suitable.*
☒	☐	For Bayesian analysis, information on the choice of priors and Markov chain Monte Carlo settings
☒	☐	For hierarchical and complex designs, identification of the appropriate level for tests and full reporting of outcomes
☒	☐	Estimates of effect sizes (e.g. Cohen's *d*, Pearson's *r*), indicating how they were calculated

Our web collection on statistics for biologists contains articles on many of the points above.

Software and code

Policy information about availability of computer code

Data collection	No custom computer codes or algorithms have been used in this work.<br><br>For the collection of data provided in this manuscript the following commercial software was used:<br><br>For GC-MS data: Data from the Shimadzu GCMS-QP2010 Ultra system was collected using GCMSsolution 4.20.<br><br>For LC-MS data: Data from the Dionex UltiMate® 3000 Quaternary Rapid Separation UHPLC focused system and Bruker Daltonics Compact qTOF mass spectrometer were collected using Thermo Fisher Chromeleon 6.80 software and Bruker Hystar 3.2.<br><br>Data from the Buchi Pure Chromatography Systems was collected using the provided Pure Navigator Software (PuriFlash® Preparative Purification Systems model: PF-5.250).<br><br>For the HPLC: Data from the Agilent 1200 series instrument was collected using the Agilent ChemStation version B.03.02 software.<br><br>For NMR data: The Bruker Avance III NMR system was controlled by the Bruker IconNMR ver.4.2 software whereas Bruker Topspin ver. 3.5 was used for data acquisition.
Data analysis	The following software was used for the analysis of data provided in this manuscript:<br><br>For the phylogenetic analysis of Ginkgo biloba CYP enzymes: the open source MEGA-X software<br>PlantSMASH software was used to analyze chromosome 5 of Ginkgo biloba for additional BGCs (http://plantismash.secondarymetabolites.org/).

For GC-MS analysis: GCMSsolution 4.20
For LC-MS analysis: Bruker DataAnalysis 4.2
For dataplotting: SigmaPlot 14
Creating figures: Inkscape 1.1
Genome analysis: CLC Main Workbench 20.0.4
NMR Analysis: Bruker Topspin ver. 3.5
Drawing Chemical structures: ChemDraw 19.1
For GC-MS analysis: GCMSsolution 4.20
For LC-MS analysis: Bruker DataAnalysis 4.2
For dataplotting: SigmaPlot 14
Creating figures: Inkscape 1.1
Genome analysis: CLC Main Workbench 20.0.4
Phylogenetic CYPome analysis: MEGA X
NMR Analysis: Bruker Topspin ver. 3.5
Drawing Chemical structures: ChemDraw 19.1

For manuscripts utilizing custom algorithms or software that are central to the research but not yet described in published literature, software must be made available to editors and reviewers. We strongly encourage code deposition in a community repository (e.g. GitHub). See the Nature Portfolio guidelines for submitting code & software for further information.

Data

Policy information about availability of data

All manuscripts must include a data availability statement. This statement should provide the following information, where applicable:
- Accession codes, unique identifiers, or web links for publicly available datasets
- A description of any restrictions on data availability
- For clinical datasets or third party data, please ensure that the statement adheres to our policy

The present manuscript did not generate new genomic DNA sequencing data but instead, for the acquisition of the results reported here we have used the Ginkgo biloba genomic DNA sequence available at doi: https://doi.org/10.1101/517946.

Additionally we used the Ginkgo biloba transcriptomes found at "Medicinal Plant Genomics Resource" (http://mpgr.uga.edu/).

The yeast codon optimized cDNA sequences used in this work are submitted at the supplementary material.

Ginkgo biloba cDNAs cloned and sequenced in this work have been deposited in GenBank under the accession numbers: GbCYP7005C1: ON759313; GbCYP7005C2: ON759314; GbCYP7005C3: ON759315; GbCYP867K1: ON759316; GbCYP867E38: ON759317; GbCYP720B31: ON759318; GbCYP720B33: ON759319; GbCYP720B39: ON759320; GbCYP720B46: ON759321; GbCYP725A8: ON759322; GbCYP725A48: ON759323; GbCYP725A49: ON759324; GbCYP728Q19: ON759325; GbCYP798B1: ON759326; GbCYP736Q2: ON759327; GbPOR1: ON759328; GbPOR2: ON759329.

The yeast strains generated in this work, as well as the codon optimized cDNA sequences related to the findings of this study are available on request from the corresponding author.

Field-specific reporting

Please select the one below that is the best fit for your research. If you are not sure, read the appropriate sections before making your selection.

- [x] Life sciences
- [ ] Behavioural & social sciences
- [ ] Ecological, evolutionary & environmental sciences

For a reference copy of the document with all sections, see nature.com/documents/nr-reporting-summary-flat.pdf

Life sciences study design

All studies must disclose on these points even when the disclosure is negative.

Sample size	For the Nicotiana benthamiana (tobacco) transient heterologous expression, all experiments were carried out in biological triplicates (three separate plants). Every experiment was repeated independently multiple times (minimum 3 times each) to verify that the results are not affected by tobacco growth conditions or by experimental errors.<br>For Saccharomyces cerevisiae (yeast) heterologous expression, yeast strains were generated in biological replicates (three independent yeast transformants) and data was obtained again through multiple independent experiments (minimum three times each), to verify that results were not affected by individual yeast growth conditions or experimental errors.
Data exclusions	No data was excluded from the analyses presented in this article
Replication	For the tobacco transiently heterologous expression we executed the experiments multiple times, independently, with biological triplicates in

every individual experiment (3 different plants were infiltrated with the specific enzyme-combination), in an effort to confirm the validity of the results obtained.

For yeast heterologous expression, we were generating 3 independent transformants for every yeast strain mentioned in this manuscript (stable genomic transformation). The 3 independent yeast transformants were cultivated independently at different time periods, to verify the accuracy of the generated products.

In both cases, all results obtained have been included in the analyses.

Randomization

Randomization was not relevant in this study as we cloned targeted cDNAs and functionally characterized the corresponded enzymes. Chemical analyses of the compounds generated by the corresponding enzymes was on specific samples, generated by specific enzyme combinations, therefore randomization was not relevant in these analyses.

Blinding

Blinding was not relevant to this work as we analyzed targeted enzymes and in our analyses we included all data generated. In our analyses, instead of blinding we used negative controls, meaning samples that did not include the relevant enzyme candidates.

Reporting for specific materials, systems and methods

We require information from authors about some types of materials, experimental systems and methods used in many studies. Here, indicate whether each material, system or method listed is relevant to your study. If you are not sure if a list item applies to your research, read the appropriate section before selecting a response.

Materials & experimental systems

n/a	Involved in the study
☒	☐ Antibodies
☒	☐ Eukaryotic cell lines
☒	☐ Palaeontology and archaeology
☒	☐ Animals and other organisms
☒	☐ Human research participants
☒	☐ Clinical data
☒	☐ Dual use research of concern

Methods

n/a	Involved in the study
☒	☐ ChIP-seq
☒	☐ Flow cytometry
☒	☐ MRI-based neuroimaging
